# Supplementary material for: Patients’ experiences of acupuncture and counselling for depression and comorbid pain: a qualitative study nested within a randomised controlled trial
Source: BMJ Open. 2014 Jun 5;4(6):e005144. doi: 10.1136/bmjopen-2014-005144 (PMC4054660; doi:10.1136/bmjopen-2014-005144)
Supplement: Author's manuscript [file bmjopen-2014-005144.draft_revisions.pdf]

Patient's experiences of acupuncture and counselling for depression and comorbid pain: a qualitative study nested within a randomised controlled trial.

Journal:	*BMJ Open*
Manuscript ID:	bmjopen-2014-005144
Article Type:	Research
Date Submitted by the Author:	27-Feb-2014
Complete List of Authors:	Hopton, Ann; Universiy of York, Health Sciences<br>MacPherson, Hugh; University of York, Health Sciences<br>Eldred, Janet; University of York, Health Sciences
**Primary Subject Heading**:	Complementary medicine
Secondary Subject Heading:	Qualitative research, Patient-centred medicine, Mental health
Keywords:	Depression, Pain management < ANAESTHETICS, PRIMARY CARE

SCHOLARONE™
Manuscripts

For peer review only

For peer review only - http://bmjopen.bmj.com/site/about/guidelines.xhtml

TITLE: Patient's experiences of acupuncture and counselling for depression and comorbid pain: a qualitative study nested within a randomised controlled trial.

Ann Hopton[1], Hugh MacPherson[1], Janet Eldred[1]

[1] *Department of Health Sciences, University of York, UK*

For peer review only

Keywords; depression, comorbid pain, primary care, acupuncture, humanistic counselling

Address for correspondence:

Ann Hopton
Research Fellow
Department of Health Sciences
University of York
Heslington
York YO10 5DD
United Kingdom
Tel: 01904 321384
E-mail: ann.hopton@york.ac.uk

Words 6,737

ABSTRACT

Introduction

Depression and pain frequently occur together, yet we have insufficient knowledge of how this comorbidity impacts on outcomes of treatment for depression from the patient's perspective. This study aimed to explore patients' experiences of depression, the processes of change within acupuncture and counselling, and the elements that contributed to longer-term change.

Methods

In a sub-study nested within a randomised controlled trial of acupuncture or counselling compared to usual care alone for depression, semi-structured interviews of 52 purposively sampled participants were conducted and analysed using thematic analysis.

Results

Differences were reported by participants regarding their experience of depression with comorbid pain compared to depression alone. Along with physical symptoms often related to fatigue and sleep, participants with depression and comorbid pain generally had fewer internal and external resources available to manage their depression effectively. Those who had physical symptoms and were receiving acupuncture commonly reported that these were addressed as part of the treatment. For those receiving counselling, there was less emphasis on physical symptoms and more on help with gaining an understanding of themselves and their situation. Over the course of treatment, most participants in both groups reported receiving support to cope with depression and pain independently of treatment, with a focus on relevant lifestyle and behaviour changes. The establishment of a therapeutic relationship and their active engagement as participants were identified as important components of treatment.

Conclusion

Participants with and without comorbid pain received acupuncture or counselling for depression, and reported specific identifiable treatment effects. The therapeutic relationship and participants' active engagement in recovery may play distinct roles in driving long term change. This study has implications for policy makers and providers of care for primary care when considering referral of patients with depression and comorbid pain.

(Words=290)

Strengths

- The 52 telephone interviews were obtained from a wide range of participants in socially diverse settings and provide rich data on the participants' experiences of depression and the treatment received in the ACUDep trial.
- The thematic analysis was conducted using a bottom-up process to allow the themes to develop directly from the participants' own words, and we present the positive and negative experiences of each form of treatment, whether treatment was beneficial or not
- Our findings identify mechanisms within the processes of change that are specific to acupuncture and counselling that facilitate reduction in the symptoms of depression

Limitations

- Participants' may have attributed changes directly to treatment rather than concurrent, coincidental contextual changes.
- There is a possibility of recall bias; however, it is likely that the participants recalled the aspects of treatment that were most salient to them.
- Telephone interviews prevented the interviewer gathering non-verbal contextual information, although this form of interview was more acceptable to participants than a face-to-face interview.

For peer review only

INTRODUCTION

Chronic pain is commonplace in half to two-thirds of participants with major depressive disorder.[2,3] The association between pain and depression becomes stronger as the severity of either increases,[4,5] and the impact of both problems on each other plays an important role in the development and maintenance of chronicity in health problems.[6] Participants with depression and comorbid pain are difficult to diagnose, feel an increased burden of disease, tend to rely heavily on health care services and are more difficult to treat.[7] Identifying and managing the pain symptoms that commonly occur alongside symptoms of depression may be important in improving depression response and remission rates.[8]

There is a growing evidence base in support of the effectiveness of acupuncture for a range of musculoskeletal conditions[9,10] however, despite its widespread use by participants[11], there has been limited evidence for acupuncture as an effective treatment option for depression.[12] The evidence for counselling as a treatment for depression is also limited[13] despite widespread utilisation in primary care in the UK, with around 90% of general practices providing on site counselling services.[14] To address this evidence gap, a randomised controlled trial (ACUDep) compared acupuncture or counselling to usual care as treatments for primary care participants with ongoing depression in primary care.[15] The results showed that both acupuncture and counselling were clinically effective in reducing depression in the short to medium term.

In a quantitative sub-study nested within this trial, which focused on the effect of comorbid pain on the outcome of treatment for depression, it was found that approximately 50% of the ACUDep participants had co-morbid pain with depression and that the participants' pain scores at baseline predicted the outcome of treatment for depression at the three-month follow up point.[16]

Not enough is known about how experiences of depression, both with and without comorbid pain, change as a result of receiving the treatments of acupuncture, counselling and usual care. The aim of this qualitative study, which is nested within the ACUDep trial, is to explore these experiences. A secondary aim is to report the aspects of treatment that patients' report might have had a positive influence on long- term change.

METHODS

Setting

This research comprised a qualitative sub-study nested within the trial of acupuncture or counselling provided as an adjunct to usual care compared to usual care alone. [15]

Participants

Within the ACUDep trial,[15] 755 participants aged 18 and over with a history of on-going depression and score of 20 or more on the Beck Depression Inventory (BDI-II) were recruited by general practitioners at 27 primary care practices across Yorkshire, Durham County and Northumberland. Participants were allocated remotely by the York Trials Unit, with the allocation code concealed from the recruiting researcher, to three groups in the proportions of 2:2:1 to acupuncture, counselling and usual care alone respectively. Within the acupuncture arm, 23 acupuncturists registered with the British Acupuncture Council for a minimum of three years delivered up to 12 acupuncture treatments per patient, usually weekly. Treatments were tailored to individual participants' needs within a trial protocol [17], which included treating symptoms according to traditional Chinese medicine theory and the integration of relevant life-style support into the treatment strategy based on acupuncture-specific advice if considered appropriate by the acupuncturists. Within the counselling arm, 37 counsellors registered with the British Association of Counselling and Psychotherapy provided up to 12 sessions of humanistic counselling delivered

For peer review only

according to a trial protocol [18] which stated; “Counsellors will use empathy and advanced listening skills to help clients express feelings, clarify thoughts, and reframe difficulties, but they will not give advice or set homework. Deviations from the trial protocol were permissible if considered necessary and recorded in the participants' log book. Usual care comprised the treatment that patients typically receive in primary care along with over-the-counter medication. Common prescribed medication included anti-depressants and analgesics. Participants were followed up over a period of 12 months after randomisation by postal questionnaire at 3, 6, 9 and 12 months.

In this nested qualitative sub-study, a purposive sampling frame was prepared to recruit interviewees in the same 2:2:1 proportion of the main trial, to include 50% males and females in each arm and to balance whether in pain at baseline or not. On receipt of their final 12 month postal questionnaire and based on a sampling frame (see Supplement 1), a sample of participants who had previously consented to be contacted for an interview were invited to engage in a telephone interview of approximately 30 minutes duration. Altogether 52 people consented to a one-to-one, audio-recorded, semi-structured telephone interview for this study.

Interviews

A researcher (AH) interviewed all 52 participants. The researcher was unknown to the participants prior to the interview, therefore the interview opened with an introduction designed to set the participant at ease, to reveal the context for their depression, and to draw out the participants' account of treatment received as part of the trial. Prompts from a prepared topic guide were used to elicit the participants' experiences of depression and treatment. (See Supplement 2) The topic guide had been piloted previously.

On average the interviews lasted approximately 25 minutes (range 11-46 minutes). To encourage participants to relax, each participant was asked to introduce themselves by speaking about things they like to do or hoped to do and then how depression had entered their lives, before moving on to the research-related questions within the topic guide. Interviews were audio-taped, transcribed verbatim and checked for accuracy. All recordings were of sufficient clarity and content that no repeat interviews were necessary. Each transcription was checked to remove any names and assigned a participant identification number.

Analytical methods

An inductive thematic analysis [19] was used to search across the dataset of 52 transcribed interviews. Using a constructivist approach to grounded theory,[20] data was analysed initially by AH by reading each transcript several times, then annotating ideas to generate a list of potential inductive codes developed from the dataset to capture and summarise the participants' experiences. Coding was performed sequentially on each transcript, initially without software, working systematically throughout the entire dataset. As codes were identified, they were recorded and organised on an Excel spread-sheet; sections of text that demonstrated that code were then added and collated moving back and forth across the data set in an iterative process.

Coding and extractions were checked by JE to verify that the participants' experiences were reflected and summarised accurately. As coding progressed, comparisons were made between codes and phrases and those with similar context or concepts were grouped together. This process was conducted within each interview and across interviews resulting in a codebook of 33 codes, seven subthemes and two themes associated with the experience of depression, (Table 1) and a codebook of 35 codes and four themes associated with the experience of the treatment; (Table2). The coding and identification of themes were discussed and developed throughout between AH, HM and JE.

For peer review only

Table 1. Coding tree; A summary of the relationships between codes, subthemes and themes for the experience of depression		
**CODES**	**SUBTHEMES**	**THEMES**
Muscular pain		
Headaches		
Gastro-intestinal pain	Pain	
Disability		
Search for diagnosis		
Tired all the time		
Exhausted- worn out		
Debilitated	Fatigue	
No energy		Reported symptoms of depression
Withdrawal		
Insomnia		
Early wakening		
Nightmares	Sleep disorders	
Too much sleep		
Low self esteem		
What other people think		
Guilt and self-blame	Negative schema	
Black and white thinking		
Family life		
Motherhood		
Caring	Home life	
Bereavement		
Isolation		
Shift pattern		
Work overload	Work strain	Contextual factors
Drive, motivation		
High bar, aiming high		
Crime		
Bullied/ threat/ physical abuse	Victimhood	
Self-harm		
Childhood		

To understand the participants' individual experiences of depression and the treatments received, the codes and themes were developed into a diagram (see Supplement 3) and populated with participants' identity numbers. This enabled the researcher (AH) to trace each participant and their journey throughout the process, and to make distinctions between those with depression and comorbid pain, and those with depression alone.

Throughout this paper the themes are illustrated with quotes that capture and embody the participants' experiences embedded within the analytical narrative as suggested by Braun and Clarke. [19] Additional illustrative quotes are set out in Supplements 4-8

For peer review only

Table 2. Coding tree; A summary of the relationships between codes and themes for the positive and negative experience of treatment; factors that influence long term change and mediating factors		
**CODES**		**THEME**
Beliefs and attitudes	Pre-treatment factors	
Previous experience of treatment		Mediating factors
Participant engagement	In treatment factors	
Therapeutic relationship		
Change of perspective	(common to all arms)	
Reduced medication		
Relaxation	(acupuncture)	
Complaints & symptoms treated		
Self-understanding	(counselling)	Positive experiences
Acceptance of situation		
Empowerment		
Effective medication	(usual care)	
Referral to NHS mental health services		
Adverse events	(acupuncture)	
Terminology		
Time consuming		
Fear of needles		
Difficulty opening up	(counselling)	
Clichéd phrases		Negative experiences
Opened a can of worms		
Expense		
Side effects of medication		
Long wait after referral	(usual care)	
Lack of general practitioner time and continuity		
Ageism		
Exercise advice		
Dietary advice		
Reduce alcohol intake	Lifestyle strategies	
Take time out		
Day to day structure		Long term behavioural change
Distraction		
Thinking strategies learned		
Self- talk & Stoicism	Cognitive strategies	
Social contact		
Seeking support		

RESULTS

Participants recruited

For peer review only

Of the 755 participants randomised in the ACUDep trial, 518 had consented to be interviewed and 674 completed their 12-month follow up. Of these, 464 (69%) were potentially eligible to join the study. Of the 61 participants invited, four declined participation and three did not respond. A total of 52 participants, comprising 24 men, and 28 women with an age range of 22 to 89 years (mean 46 years, SD 13.8) were interviewed. At baseline, 26 of these participants had reported having moderate or extreme pain or discomfort on the EQ5D questionnaire; these people formed the pain group, the remainder formed the no-pain comparator group. As part of the ACUDep trial, 22 of the 52 had been randomised to receive acupuncture, 20 to counselling and 10 received usual-care alone. A summary is presented in the sampling frame in Supplement 1. On average, those allocated to acupuncture attended 11 sessions (range 4-12) and those allocated to counselling attended 10 sessions (range 6-12).

Symptoms experienced with depression and contextual factors

Participants reported a range of symptoms they experienced concurrently with depression: predominantly, pain, fatigue and sleep disorders (see Supplements 3-4). Most participants with depression and comorbid pain suffered from either persistent headaches or moderate to extreme muscular skeletal pain that predated the onset of depression and compromised their mobility, to the extent that four men had considered suicide, and one man had attempted suicide. Almost half of those in pain experienced sleep disturbances or an overwhelming loss of energy to the point where much of their time was spent in bed, withdrawing from social and day-to-day activities. (See Supplement 3)

> *"There are times when things like headaches and neck aches prevent sleep... I sort of drift 48 hours without sleeping... I have in the past been, if you like, down for a prolonged period of time...just sleep and sleep and sleep and wake up and do something for half an hour and then go back to sleep again. I can just about function with pain killers... The only time I did try to top myself that led to even more depression because I couldn't even do that right! I'd taken a boat load of the diazepam I was on, to try and calm me down. I'd sort of stock piled some of that…washed it all down with Glenfiddich, and instead of, you know, just shuffling off quietly, all I did was end up waking up feeling absolutely dreadful in a puddle of my own vomit, and it was one of those things where, you know, it took me weeks afterwards, thinking well, I can't even kill myself properly."*(p25,M,coun)

With regards to contextual factors, for the majority of the pain group, the pain they experienced had compromised their ability to work. Very few had social support, and for some, their being at home meant that they were the family member available to take on a caring role for a relative, which incurred further stress, ill health and isolation. (See supplement 5)

> *"Because I was off and not working I was able to have the time to look after my elderly mother and aunt who were both in their 80s. So I was their carer for 4 years and unfortunately I lost both of them and my father 4 years ago, within 6 months of each other...My IBS is certainly related to depression yeah...When I was looking after the old dears, as I call them, I was offered an operation twice and I turned it down because I didn't want to be, you know, incapacitated and not able to look after them. Once they passed on, I was then able to address my own health problems."* (p26,M,coun)

In summary, for the participants with depression and comorbid pain, the symptoms experienced impacted on the most basic level of physiological needs, and reduced their ability to engage in social activity, whilst the contextual factors compromised their security through reduced income. Together

For peer review only

these factors suggest that this group of people have few internal and external resources remaining to effectively manage their depression.

Of the participants who were pain-free at baseline, several people complained of tension headaches or gastro-intestinal symptoms they experienced at times of heightened stress and anxiety. Several others identified distinct patterns of disordered sleep; either difficulty in settling to sleep or a pattern of early wakening. Similar to the pain group, a few tended to withdraw at times when they felt particularly low in mood.

> *"I get a lot of stomach problems actually when I feel depressed...And of course, really tired as well – very, very tired when I'm feeling down…Sometimes I find it really hard to cope with people. I can possibly be a bit grumpy sometimes, or really quiet. Because I can't really face talking to anybody on certain days...I just can't bear it."* (p57,F,uc)

Regarding contextual factors, the majority in the no-pain comparator group were in full or part-time employment or were relatively affluent retired professional people. For many, their experience of depression concerned feelings of low self-esteem brought about by high expectations of themselves within their working life, or hectic social schedules. Others experienced low self-esteem and threats to their security from bullying at work or as a victim from domestic violence.

> *"We're also dance teachers, which is supposed to be a hobby but has somehow involved taking over our lives... we're teaching three nights a week, so it's a bit of a commitment for a hobby... Because when we started teaching last May time... that did give me more of an impetus to actually make the effort, because once you've got teaching you've got to go, because you're going to let people down. And I'm always glad that I do. If I'm having low days now, I'm always glad that I've been, because I'm concentrating on stuff that's completely outside of me or completely outside of my normal life... One of the things that's come through is that I hate letting people down. I'm very hard on myself."* (p45,F,coun)

> *"I tend to keep my things bottled up... And it really gets me down. My work suffers. My home life suffers. And everything suffers if I'm really bogged down with something….following a particular incident, or a series of incidents... I chose to or had to be the brunt for a lot of the aggression and violent behaviour that this man displayed at the time. So if you like, there was a particular traumatic... there was a starting point for it."* (p38,M,coun)

In general, the no-pain comparator group experienced fewer demands physiologically. Most had their basic security and social needs met, this group had larger reserves of both internal and external resources available to them to cope with their depression.

Processes of change reported by those receiving acupuncture: positive and negative experiences

The processes of change identified within the data formed three stages: primarily, developing a therapeutic relationship; secondly, the individual diagnosis and treatment of symptoms; and finally, engendering changes in health behaviours. Within the pain group and the no-pain comparator group, the positive experiences tended to facilitate the process of change at each stage, whilst negative experiences contributed to the barriers to change. Most participants welcomed the opportunity to try something different for their depression. The acupuncturists' understanding of their symptoms and explanations of how acupuncture might help their particular problems initiated the development of a therapeutic relationship. In contrast, two people described their acupuncturist as brisk, efficient and professional, yet lacking in bedside manner.

For peer review only

> *"She was very positive about things...I think you have more of an intimate relationship with the person doing that rather than just a person in an office somewhere. You're physically involved." (*p11,M,acu)

> *"They could do with a course in empathy."* (p22,F,acu).

Within the second stage of treatment, for most participants, the therapeutic relationship was further fostered through the acupuncturist listening to the participants' concerns, and treating the symptoms of depression depending on what was diagnosed to be of most importance to the participant at that point. Within the pain group several people experienced relief from musculoskeletal pain which tended to last for a few hours or days after the session and improvement commonly built up over several sessions. Several also reported feeling deeply relaxed during the sessions and an uplifting sense of well-being afterwards.

> *"I thought it was quite a strange sort of feeling, but I sort of felt better quite quickly. And then I went to the second and the third, it was… it completely lifted my mood and it made me feel more motivated…It was almost as if a weight had been lifted off my head and all of a sudden I felt like some energy had come back."* (p3,M,acu)

With regards to negative experiences, one woman reported included extreme tiredness after the acupuncture session, a problem that was addressed by the acupuncturist by adjusting treatment during the next session, and one man with extreme back pain attributed needling pain to his damaged nerves from a previous injury. Both of these participants concluded that the treatment went well, however they also reported, *"It was not for me."* (p4,M, acu; p7,F,acu)

> *"she'd actually hit at the root for a problem that I have with my pain, because I think at one time she put a needle in me and I kicked her. Without wanting to I involuntarily kicked her. And she'd obviously hit upon -- I think there was one time when I kicked out and more than a few occasions where she'd twitched a nerve that obviously."* (p4,M,acu)

In contrast, within the no-pain group two participants who were worried about the potential pain of needling prior to starting the treatment later attributed the needling sensations to the healing process. Three men thought they would have been equally relaxed if they had just rested or gone for a massage, and two other participants found the sessions too time consuming.

> *"A little bit sceptical as to whether the treatments (acupuncture) work anyway. So it was for me like, if I go and get a sport massage, it was like the equivalent of that..."* (p13,M,acu)

Factors that influenced long-term change reported by those receiving acupuncture.

As treatment progressed, many participants reported that their acupuncturist began guiding them to make changes to their lifestyle in order to engender beneficial long-term outcomes. For most people with pain, fear of pain and potential injury posed a barrier to engaging in physical activity. The majority of the pain group reported being encouraged to take up gentle exercise for their overall health and they also distracted themselves during periods of low mood.

> *"The things, they seem so small, but they are important. Things like, getting out and going for a walk and getting some fresh air. And just opening your eyes in the mornings and trying to cope with life."* (p7,F,acu)

> *"He started about exercise, you know, how that can make you feel more up…"* (p8,acu)

For peer review only

"I read a lot and try to keep my mind off it. Really." (p9,F,acu)

One man developed his own technique based on how he felt during the acupuncture to help him manage low moods, whilst another relied on monthly acupuncture treatments alone to stay well; another considered further treatments but found the cost prohibitive.

"I sort of developed this technique and I don't know, it was like… The way I was feeling during the acupuncture... I sort of clung on to this feeling that, or this technique of gaining that feeling, so I remember on a couple of occasions where I was out and about walking, and thinking about things that would normally would start leading me to start feeling a bit down, but it was like I'd been given this tool in my head and I just sort of – it just sort of went onto auto-pilot. It was like pulling those feelings away and just sort of throwing them away....Well, it lasted for a while but it started subsiding." (p1,M,acu)

"I go for acupuncture now once a month and I find that any more than a month and I can feel myself sort of slipping and feeling really, you know, starting to get worse again. And then I go and I feel much, much better…" (p3,M,acu)

The advice given to the no-pain comparator group was qualitatively different; Acupuncturists advised on dietary change, the reduction of caffeine and alcohol, and relaxation, which varied with the presenting symptoms, and by gender. Those participants with least rapport also tended to be those who were less willing to make behavioural changes.

"She also helped with giving me other things that I can do. Suggesting different foods for me to eat to make me feel more energetic.... I was cold all the time and that made me feel more lethargic as well, because all I wanted to do was stay in and go to bed and stay warm. So she was suggesting that I literally ate warmer foods, and she gave me a list of sort of Chinese medicine sort of foods that they had." (p20,F,acu)

In general, the process of change evolved in three stages. The therapeutic relationship and active engagement in recovery acted as mediators of the outcome throughout each stage of the process. In the short term, acupuncture often relieved physiological symptoms of depression and of comorbid pain. Longer-term improvement in depression was developed through the participants' active engagement in health promoting behaviours, supported by a positive therapeutic relationship. Several participants with comorbid pain had less physical ability to engage in lifestyle changes and tended to be the passive recipients of care. These participants often had fewer external resources in the form of finance and social contact to manage their depression and comorbid symptoms in the longer-term. Additional quotes are presented in Supplement 6.

Processes of change reported by those receiving counselling: positive and negative experiences

Based on their previous experiences of counselling the majority of participants spoke of their low expectations of counselling. However when engaged with the counselling process within the trial, most reported being relieved to have someone to talk to in confidence. For both the pain and the no-pain groups, the process of change followed a common pathway: beginning with the participants' disclosure of personal information and being listened to.

"I found that process to be very valuable…I found X was very much listening and empathising, but maybe offered interpretation a bit more than the National Health person." (p37,M,coun)

For peer review only

For most participants this two-way active engagement appeared to nurture a therapeutic relationship between the participant and counsellor. Four male participants welcomed the opportunity to speak to a male counsellor, a choice which put them at their ease, and facilitated the process. Three others found difficulty engaging with their counsellor and attributed this problem to a personality clash. This presented an early barrier to the process of change.

> *"A lot of it does depend on who the counsellor is…I'm saying probably same sex works better. They probably have a clearer understanding of the male mind... I found him particularly sympathetic and, you know, very constructive. I think that was...I was very pleased with the way it went..."* (p40,M,coun)

> *"Every single counselling cliché that you have about, oh it's parenting issues – she kind of wheeled them all out one after the other and they were already things that I'd thought about, considered and looked at and examined to the nth degree and then thought, no that's not the problem... It felt like she was reading a script almost – like a guidebook to deal with this kind of disorder...it was almost the complete opposite of what I felt like I needed."* (p28,M,coun)

A second stage of the process of change was often identified as occurring around midway within the course of treatment. The iterative process of participants' disclosure continued, with deeper exploration of their past, which helped to clarify the participant's understanding of themselves and their situation.

> *"At the end of it, it actually for me opened up a can of worms really, and I think it did me more harm than good. A lot of my problems, especially with low self-esteem, come from the way I was brought up by my parents and my father especially. And its stuff that I'd never addressed and it brought it all out, actually. And I actually felt worse at the end of it... I don't feel so bad about it now, because I recognise why I am like I am and some of the problems I have, where they come from... Although I say they'd opened up a can of worms, and brought some upsetting experiences back... I think it was good to do that. Because those sort of things had been bottled up for many, many years... it's actually made me address them."* (p26,M,coun)

> *"It made me realize that I just held everything in. From being a little girl, everything that had ever bothered me it was never talked about. You know, I'm quite lucky that I've never had any real abuse or anything like that. It's just that I've got memories of being a child and things were said and it hurt. And I just locked it away. And I did that for years."* (p41,F,coun)

Several realized what factors triggered and perpetuated negative thoughts, some of which were unfounded. For one man with chronic pain this meant going through a grieving process for the loss of his former way of life before setting in place new ways of thinking and coping.

> *"You know, I think that was the big thing that I got from it, you know – that I could see myself more positively after having the time with him. And understand that some of the negative thoughts that were coming to my mind were not reality, if you like. To let them sail past and focus on the good things that I've done in the past."* (p38,M,coun)

> *"Everything that defined what I was has now gone. And it took an awful lot of grief, if you like, to come round to the fact that it was worth trying again".* (p25,M,coun)

For peer review only

The use of metaphors was particularly useful for de-cluttering unnecessary thoughts about their past; regaining perspective; setting their problems into context, and focussing on what was important.

> *" the discussions were much more free than I'd kind of anticipated they might have been, was using metaphors and analogies and stuff like that, to be able to describe things and move through things. And the pictures were just coming to me in my head, like. I had one which was sort of like a circuit board and it felt like some of the wires were not quite wired up properly and they weren't working and stuff like that. And I can kind of track the metaphors throughout the whole process and it feels like it was much more of a – like it all opened up."* (p38,M,coun)

Factors that influenced long term change reported by those receiving counselling

The final stage in the process of change was directed towards enabling the participants to maintain progress independently. Gender differences became apparent in the coping strategies adopted; the majority of women took up health and well-being strategies. Compared to the women in the pain group, the women in the no-pain comparator group were able to use a wider range of resources to cope with, and engage in social activities more easily. One woman recalls being given cognitive behavioural homework to overcome a particular anxiety.

> *"She gave me sort of little exercises. I found it very difficult to walk down to a friend of mine. She lives in quite a built up area... People were sitting out in their gardens and I found it very intimidating. I didn't like it. I'd become really sweaty, short of breath walking down through her estate to go and see hear... basically she just taught me to get a grip on myself really, by pointing out, you know, that everything was going to be all right... short sharp steps really. And that I'd got the coping mechanisms and I could do it."* (p32,F,coun)

Many male participants appear to have continued to practise the cognitive strategies learned within the earlier sessions, and applied them to their life outside the sessions. However, male participants with depression and comorbid pain found greater difficulty sustaining these strategies and returned to their general practitioner for further help.

In summary, the majority of participants had had previous experience of counselling, however their initial low expectations of success receded as the course of treatment progressed. A few counsellors practiced a more directive intervention than humanistic counselling, according to the need of the participant. The process of change comprised three stages, each mediated by the quality of the relationship and the participant's active engagement. Additional quotes are presented in Supplement 7.

Processes of change reported by those receiving usual care

Participants in all three arms received usual care throughout the trial. The process of change within usual care was less evident. Differences in the appraisal of general practitioner care were apparent: three older participants with depression and comorbid pain who were allocated to usual care alone complained of a lack of understanding and continuity of general practitioner and they felt abandoned without hope. One 89-year-old lady reported:

> *"I would never go to a doctor again. I am, because I suffer a lot of pain that I needn't have done if he'd been different…if he'd have listened to me instead of just pooh-poohing it off and saying, oh no, it's not that. If he'd have really listened to what I was saying, he could have done more for me...he's ignored what I've told him. Well, in fact he's very often just ignored it all together. Pretended I hadn't said it... You see, at my age you can't really change*

For peer review only

> *doctors. There's not many doctors want to take somebody on that's 90 years old, do they? When I'm having a really bad day... you know, and I feel I can't turn even to the doctors, you know, then yeah, I do get depressed."* (p51,F,uc)

In contrast, the majority of participants who were pain free pointed to a relationship based on trust as being a component of their steady improvement over time. For most people a regular monthly 10 minute consultation was helpful and constructive. A few felt that their general practitioner had made additional time for them when they were most in need.

> *"He sees me every month. I have monthly meetings with him just to have a general chat about how things are... I get on fine with him. As I say, I can talk about just about anything with him, so I suppose in a way, he's sort of, if anything he's been maybe a counsellor for me, because, you know, I can sit and talk to him about stuff..."* (p58,M,uc)

All participants at some time had been prescribed antidepressant medication. The majority of participants on long term antidepressant medication raised concerns about the side effects; participants in the pain group were particularly concerned about the potential effects of mixing medication for their other medical problems with their antidepressants. A few acknowledged that they needed antidepressants to maintain long term stability.

> *"Staying on medication, it has transformed my life and made everybody else's life around me better as well. And I wish I'd have done it sooner... It must be four or five years now and yes, it's been life transforming... When I started on my medication and I could realize the difference – the two people I was, almost."* (p7,F,acu)

In addition to medication, during the period of the trial general practitioners referred participants to a range of secondary services: two young women received three sessions with a mental health link worker, an intervention which had enabled them regain control of their lives; three others had been advised to try online cognitive behavioural therapy which two found to be easily accessible and effective; one man at risk of suicide and was referred for urgent psychiatric help.

> *"I was referred rapidly to A&E and was assessed by X the psychiatrist. And I was put on to intensive home treatment. Which was invaluable. As a condition of not being sectioned..."* (p59,M,uc)

Referrals for younger patients had been beneficial although the waiting times were long and not always found to be acceptable, leaving most patients without adequate support in at a time of crisis, and without sufficient money to pay for private care.

> *"I go to the doctors and I have to wait a matter of I don't know how many months before I can get, you know, onto the counselling and you know, it's just like the moment's gone, sort of thing... Unfortunately that can cost a lot of money – I'm on benefit. I can't afford it...I don't hold out much hope."* (p50,F,uc)

Factors that influenced long term change reported by those receiving usual care: positive and negative experiences

For those who received help via a referral, the advice followed a familiar pattern; to engage in lifestyle changes; to add structure to the daily routine and to use distraction to reduce the focus on the symptoms and negative feelings. However, without support the stoicism of 'forcing myself 'was a prominent default strategy among most usual care participants

For peer review only

"I force myself to do things and then I generally feel better" (p57,F,uc)
"It's forcing myself. Well, it's a survival strategy..."(p59,M,uc)

Overall, the continuity of always seeing the same general practitioner was reported to be important and beneficial. By contrast some older participants with depression and comorbid pain remained caught in a seemingly hopeless cycle of seeking diagnosis and treatment for a physical complaint and without resources to seek private health care services. Most patients who received acupuncture or counselling were also happy with the attention from their general practitioner, but had welcomed the additional treatment provided within the trial as an adjunct to their usual care. Supplement 8 presents a number of representative quotes.

DISCUSSION

Principal findings

The participants' experiences of depression were a complex interplay of internal and contextual factors. Compared to participants with depression alone, participants with depression and comorbid pain had fewer internal and external resources available to effectively manage their depression in the longer-term. Processes of change comprising three stages were identified within acupuncture and counselling, each with specific active components. For both interventions, participants reported that the establishment of a therapeutic relationship and their active engagement helped them develop coping strategies that in turn helped them be more effective in reducing their depression in the longer-term. Gender differences were apparent; the majority of women utilised a wide range of health behaviours, distraction and social contact, whilst men relied predominantly on cognitive strategies to manage unhelpful negative thought processes.

Strengths and Limitations

Qualitative analysis of participants' reports of acupuncture and counselling compared with usual care provided within a randomised controlled trial is novel. This study was nested within a 12-month randomised controlled trial of the effectiveness of acupuncture or counselling for depression compared to usual care. The 52 telephone interviews were obtained from a wide range of participants in socially diverse settings. The interviews provided rich data on the participants' experiences of depression and the treatment received in the trial. The thematic analysis was conducted using a bottom-up process to allow the themes to develop directly from the participants' own words. We have presented the positive and negative experiences of each form of treatment, whether treatment was beneficial or not, and we are able to enrich the quantitative results of effectiveness of the treatments offered with the qualitative data.

These qualitative findings are concordant with, and supplement the quantitative data [16] from the ACUDep trial which showed that participants with moderate to extreme pain at baseline had worse outcomes at three months for depression than the no-pain comparator group in all three treatment arms. Our findings extend the findings of the trial's quantitative data in two ways: firstly, they offer insight into how pain and disability may erode the internal resources available for the effective management of depression. Moreover, these limitations compromise the person's security by reducing ability to generate external resources such as financial income and social contact. Secondly, based directly on participants' accounts, our findings identify mechanisms within the processes of change that are specific to acupuncture and counselling that facilitate reduction in the symptoms of depression.

Our study has some limitations. Participants' may have attributed changes directly to treatment rather than concurrent, coincidental contextual changes. There is a possibility of recall bias as it has long been known that there is a significant, stable association between depression and memory

For peer review only

impairment [1] which may have altered what was recalled and how it was recalled. However, our aim was to learn more of the experiences of depression and treatment in the longer-term and it is likely that the participants recalled the aspects of treatment that were most salient to them. The lack of face-to face-contact during the telephone interviews prevented the interviewer gathering non-verbal contextual information such as social cues, body language, appearance, and setting to supplement the verbal answers of the interviewees. However, a recorded telephone interview was expected to be more acceptable to participants than a face-to-face interview in terms of time and anonymity.

Comparison to other studies

That depression in the presence of pain is associated with a poorer response to treatment for the depression corresponds with previous studies of depression and pain comorbidity.[3,8] Patients with depression and comorbid pain tend to exhibit a cognitive bias specific to negative aspects of health and are more likely to report less favourable outcomes of treatment.[25] Many of the pain group participants had a musculoskeletal problem alongside their depression, and complained of fatigue and sleep disturbances. This cluster of symptoms has also been identified in 36% of older people suffering from osteoarthritis of the hip and knee. [21]
The characterisation of depression as a cycle of pain, fatigue and withdrawal that impacts on daily functioning, social activities is consistent with evidence showing that these factors create an enduring cycle of depression.[7,22] The cyclical nature of pain problems are known to activate catastrophic worry and accentuate the symptoms of depression; coping strategies such as relaxation and distraction techniques are a good way of regulating emotions if the pain is not too intense.[6]
Older people with chronic pain and depression were identified in this study as the least satisfied with their primary care service, a finding which echoes earlier findings where patients with multiple physical complaints and depression posed a greater clinical burden[3,8] and were perceived as 'difficult' by general practitioners.[2]

With regards to the mechanisms of change, our findings identify three clear stages within acupuncture and counselling. The establishment of the therapeutic alliance in the early stages is an essential component from the outset of treatment. This extends the findings from within a pragmatic trial of acupuncture for back pain[23,24] and supports an earlier model of the process and mechanisms that contribute to ongoing change in counselling developed from the user perspective.[25] Historically, the therapeutic alliance has been regarded pejoratively as a placebo 'feel good factor' based on the grounds that most individuals seek positive feedback to reinforce their own behaviour.[26] However, where this argument focuses on 'visiting' a therapist for advice and help, it misses the point that the intervention-specific advice and positive reinforcement used in conjunction with the participant's active engagement in their rehabilitation will activate beneficial behavioural change.[27] An earlier study found that some participants had difficulty putting self-care advice into practice, even when they were intellectually committed to and suggests that practitioners may need to follow up more carefully on the advice they have given.[28]

Implications for practice and future research

Previous work has advocated that the management of depression and comorbid pain should involve the treatment of both physical and psychological components together, and the treatments should be customised and directed to addressing comorbidities.[29] Psychiatrists and general practitioners often feel ill-equipped to adequately manage the complex presentation of symptoms associated with depression and comorbid pain. A shift in care is required from the current focus on the medical aspects of physical health to an all-encompassing approach that takes into account the bio-psychosocial effects of depression and comorbid pain.[30] Future research should investigate the effectiveness of using a sequential strategy of acupuncture for early relief of symptoms, especially

For peer review only

where there are physical symptoms, followed by counselling to address deeper psychological issues and develop cognitive coping strategies to break out of the cycle of depression.

CONCLUSION

Differences in the way depression is experienced by people with depression and comorbid pain impact on the participant's engagement with treatment and on the response to treatment for depression. The processes of acupuncture and counselling had specific identifiable effects that were beneficial to the majority of participants. The therapeutic relationship and participants' active engagement in recovery may play distinct roles in driving long-term management of depression and comorbid pain. This study has implications for policy makers and providers of care for primary care patients with depression and comorbid pain.

Legend Supplement 3.

Diagram showing the participants' experiences of depression and treatment.

Numbers indicate participants reporting; Bold = participants with chronic pain; ^{D} = disabled

Acknowledgements

We acknowledge the contribution of the patients, counsellors, acupuncturists and general medical practitioners; the ACUDep trial management team; and Anne Burton for transcription services.

Contributors

Ann Hopton (MSc) is a female research fellow from a nursing background whose research focuses on the non-pharmacological management of chronic pain and depression. Ann Hopton conducted the interviews and analysis, interpreted the data, drafted and revised the article.

Dr Janet Eldred is a female qualitative researcher and research administrator whose interests are based on feminist theology and older peoples' research. Janet Eldred assisted with coding, advised on analysis and gave final approval for the paper publication.

Dr Hugh MacPherson is a practising acupuncturist and senior research fellow specialising in the effectiveness, cost-effectiveness, mechanisms and safety in the evaluation of complementary medicine. Hugh MacPherson revised critically important intellectual content and gave final approval for publication.

Ethics

Ethical approval was obtained from the York NHS Research Ethics Committee (09/H1311/75).

Funding

Funding: This article presents independent research funded by the National Institute for Health Research (NIHR) under its Programme Grants for Applied Research Programme (RP-PG-0707-10186). The views expressed are those of the author(s) and not necessarily those of the National Health Service, the NIHR or the Department of Health. The funders, and the University of York in its role as sponsor for the study, had no role in study design, data collection and analysis, decision to publish, or preparation of the manuscript.

For peer review only - http://bmjopen.bmj.com/site/about/guidelines.xhtml

REFERENCES

1 Burt DB, Zembar MJ, Niederehe G. Depression and memory impairment: A meta-analysis of the association, its pattern, and specificity. *Psychological Bulletin* 1995;**117**:285–305. doi:10.1037/0033-2909.117.2.285

2 Katona C, Peveler R, Dowrick C, *et al.* Pain symptoms in depression: definition and clinical significance. *Clinical Medicine* 2005;**5**:390–5. doi:10.7861/clinmedicine.5-4-390

3 Arnow BA. Comorbid Depression, Chronic Pain, and Disability in Primary Care. *Psychosomatic Medicine* 2006;**68**:262–8. doi:10.1097/01.psy.0000204851.15499.fc

4 Bair M, Robinson R, Katon W, *et al.* Depression and pain comorbidity. *ArchInternMed* 2003;**163**:2433–45.

5 Kroenke K, Wu J, Bair MJ, *et al.* Reciprocal Relationship Between Pain and Depression: A 12-Month Longitudinal Analysis in Primary Care. *The Journal of Pain* 2011;**12**:964–73. doi:10.1016/j.jpain.2011.03.003

6 Linton SJ, Nicholas MK, MacDonald S, *et al.* The role of depression and catastrophizing in musculoskeletal pain. *European Journal of Pain* 2011;**15**:416–22.

7 Tylee A, Gandhi P. The Importance of Somatic Symptoms in Depression in Primary Care. *Prim Care Companion J Clin Psychiatry* 2005;**7**:167–76.

8 Kroenke K, Shen J, Oxman TE, *et al.* Impact of pain on the outcomes of depression treatment: Results from the RESPECT trial. *Pain* 2008;**134**:209–15. doi:10.1016/j.pain.2007.09.021

9 Vickers AJ, Cronin AM, Maschino AC, *et al.* Acupuncture for Chronic Pain: Individual Patient Data Meta-analysis. *ArchInternMed* 2012;:1–10.

10 Lam M, Curry P. Effectiveness of Acupuncture for Nonspecific Chronic Low Back Pain: A Systematic Review and Meta-analysis. *Spine* 2013;**38**:2124–38. doi:10.1097/01.brs.0000435025.65564.b7

11 Hopton AK, Curnoe S, Kanaan M, *et al.* Acupuncture in practice: mapping the providers, the patients and the settings in a national cross-sectional survey. *BMJ Open* 2012;**2**.http://bmjopen.bmj.com/content/2/1/e000456.abstract

12 Smith CA, Hay PP. Acupuncture for depression. *CochraneDatabaseSystRev* 2005; CD004046.

13 Bower P, Knowles S, Coventry PA, *et al.* Counselling for mental health and psychosocial problems in primary care. *Cochrane Database Syst Rev* 2011; CD001025. doi:10.1002/14651858.CD001025.pub3

14 Barnes D, Hall J, Evans R. Survey of the Current Provision of Psychological Therapy Services in Primary Care in the UK. Artemis Trust 2008.

For peer review only

http://www.bacp.co.uk/admin/structure/files/pdf/2440_pl_438_artemis_report_web_small.pdf (accessed 10 Feb2014).

15 MacPherson H, Richmond S, Bland M, *et al.* Acupuncture and Counselling for Depression in Primary Care: A Randomised Controlled Trial. *PLoS Medicine* 2013;**10**:e1001518. doi:10.1371/journal.pmed.1001518

16 Hopton AK, MacPherson H, Keding A, *et al.* Acupuncture, counselling or usual care for depression and comorbid pain: secondary analysis of a randomised controlled trial. 2014.

17 MacPherson H, Schroer S. Acupuncture as a complex intervention for depression: a consensus method to develop a standardised treatment protocol for a randomised controlled trial. *Complement TherMed* 2007;**15**:92–100.

18 MacPherson H, Richmond S, Bland MJ, *et al.* Acupuncture, Counseling, and Usual care for Depression (ACUDep): study protocol for a randomized controlled trial. *Trials* 2012;**13**:209.

19 Braun V, Clarke V. Using thematic analysis in psychology. *Qualitative Research in Psychology* 2006;**3**:77–101.

20 Charmaz K. *Constructing Grounded Theory: A practical guide through qualitative analysis.* London: Sage 2006.

21 Murphy SL, Lyden AK, Phillips K, *et al.* Subgroups of older adults with osteoarthritis based upon differing comorbid symptom presentations and potential underlying pain mechanisms. *Arthritis Research & Therapy* 2011;**13**:R135. doi:10.1186/ar3449

22 Alderson SL, Foy R, Glidewell L, *et al.* How patients understand depression associated with chronic physical disease – a systematic review. *BMC Family Practice* 2012;**13**:41. doi:10.1186/1471-2296-13-41

23 MacPherson H, Thomas K. Self-help advice as a process integral to traditional acupuncture care: implications for trial design. *Complement TherMed* 2008;**16**:101–6.

24 Hopton A, Thomas K, MacPherson H. The Acceptability of Acupuncture for Low Back Pain: A Qualitative Study of Patient's Experiences Nested within a Randomised Controlled Trial. *PLoS ONE* 2013;**8**:e56806. doi:10.1371/journal.pone.0056806

25 Perren S, Godfrey M, Rowland N. The long-term effects of counselling: The process and mechanisms that contribute to ongoing change from a user perspective. *Counselling and Psychotherapy Research* 2009;**9**:241–9. doi:10.1080/14733140903150745

26 Wall M, Wheeler S. Benefits of the placebo effect in the therapeutic relationship. *Complementary Therapies in Nursing and Midwifery* 1996;**2**:160–3. doi:10.1016/S1353-6117(96)80050-3

For peer review only

27 Bair MJ, Matthias MS, Nyland KA, *et al.* Barriers and Facilitators to Chronic Pain Self-Management: A Qualitative Study of Primary Care Patients with Comorbid Musculoskeletal Pain and Depression. *Pain Medicine* 2009;**10**:1280–90. doi:10.1111/j.1526-4637.2009.00707.x

28 Evans M, Paterson C, Wye L, *et al.* Lifestyle and Self-Care Advice Within Traditional Acupuncture Consultations: A Qualitative Observational Study Nested in a Co-Operative Inquiry. *The Journal of Alternative and Complementary Medicine* 2011;**17**:519–29. doi:10.1089/acm.2010.0749

29 Lavigne GJ, Nashed A, Manzini C, *et al.* Does Sleep Differ Among Patients with Common Musculoskeletal Pain Disorders? *Current Rheumatology Reports* 2011;**13**:535–42. doi:10.1007/s11926-011-0209-3

30 National Collaborating Centre for Mental Health. CG91: Depression in adults with a chronic physical health problem: Treatment and management. British Psychological Society & the Royal College of Physicians 2010. http://www.nice.org.uk/CG91

For peer review only

Supplement 1.

Sampling frame of the participants interviewed:

NB. Declined =4; No response=3

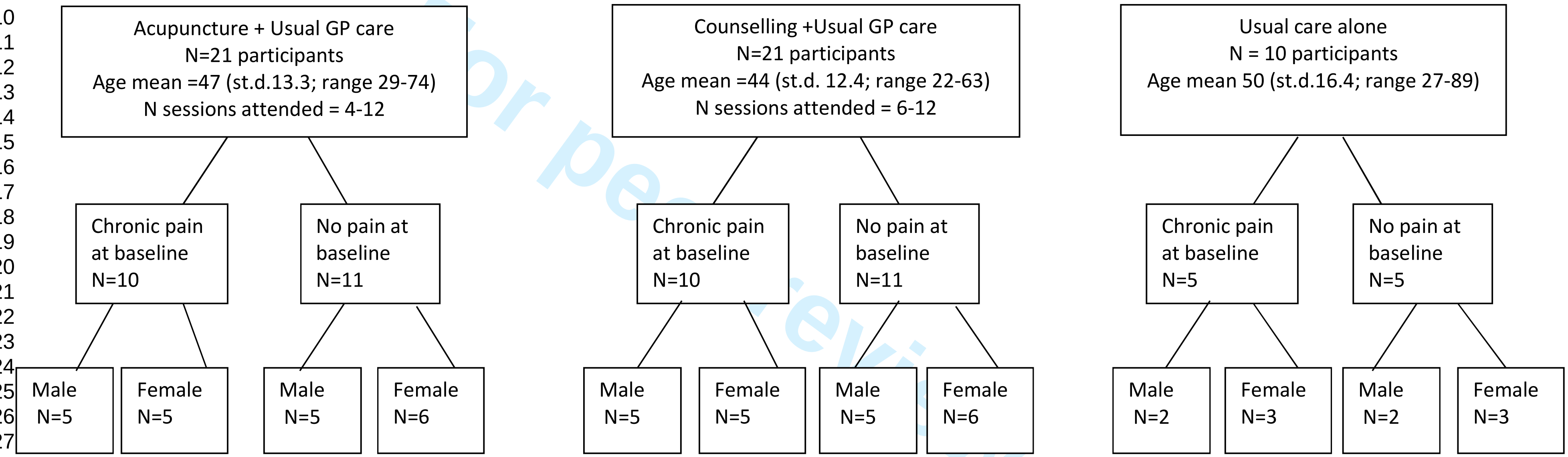

For peer review only

Supplement 2: Topic Guide for in-depth interviews

1. Background information:
    a) sociological profile: age gender
    b) social support: family, friends, colleagues, carers
    c) activities: physical exercise - outdoors-indoors, leisure activities, activities that expressly involve aesthetics, resting
    d) interests: hobbies, dreams, imagination, future plans
    e) food: diet, cooking, eating out
2. Personal experience of depression, co-morbidity
3. Previous experience with health services and treatments for depression
4. Experience and perceptions of the actual treatment (acupuncture, counselling and usual care) received within the trial
5. Perceived outcomes of treatment
6. Personal beliefs about the effectiveness of different types of treatments
7. Personal preferences for different types of treatments

Research Question 1)

To what extent is pain also present with depression, and how has the depression (and pain if present), changed over time as a response to receiving a course of acupuncture or counselling?

1) Could you tell me a little bit about yourself and about things you like to do, or hope to do.
   Prompt
- How has your depression affected your life over the past few months/years?

2) Could you tell me about your experience of the treatment you received for depression during the trial?
   Prompt
- It's been 12 months since your treatment started, what changes have you noticed during that time

For peer review only

Research Question 2)

What aspects of the treatment influenced long term change?

1) Has the treatment helped you understand/learn more about your depression?
   Prompt
   - Are there things you can do that will help to manage it?

2) What was it about the treatment that helped you understand or learn more about depression? And your pain?
   Prompts
   - Have you learned anything about how to manage your depression or pain? (If so, what?)
   - Have you adopted any different ways of coping with your depression or pain?

3) Has your understanding of yourself changed in any way during the trial?
   Prompt
   - How do you feel about yourself now compared to before?

Research Question 3)

How satisfied are you with the treatment received?
Prompts

- How did the treatment you received on the trial compare to treatment you have had before? e.g. GP, mental health service, private, other…?
- What aspect of the treatment did you like/dislike /gave you concern and why?
- Do you think the other treatments would be more or less effective

For peer review only

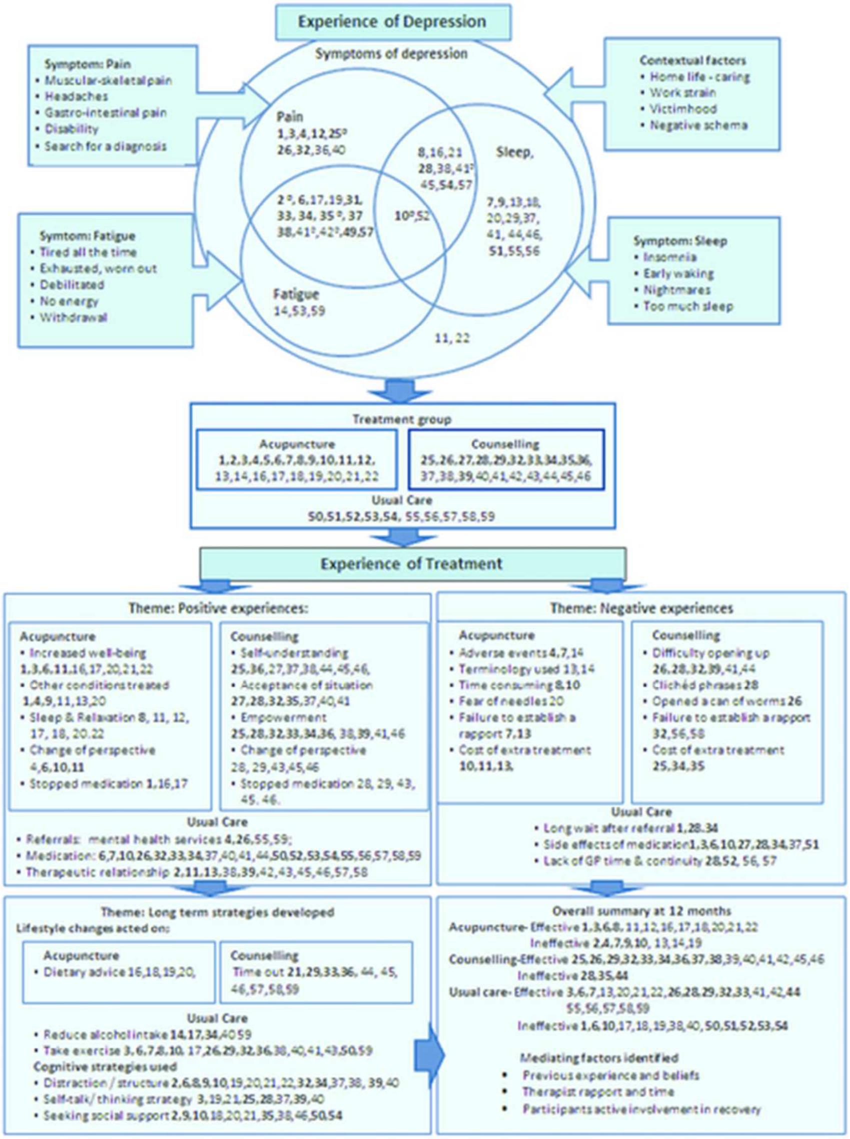

Legend Supplement 3.
Diagram showing the participants' experiences of depression and treatment.
Numbers indicate participants reporting; Bold = participants with chronic pain; D = disabled

20x27mm (600 x 600 DPI)

Supplement 4. Symptoms of depression
“When I'm feeling really, really quite down, I find it difficult to actually get on with people. I'm very, very on the - I don’t know whether the offensive, would be the right word, in the sense that I tend to read into things too much. Or take things far too literally…And obviously the defences go out there as well, so yeah. I do things without actually realising that in the sense that I might actually snap at people. So my behaviour does change.” (p1,M,acu)
“I don t want to do things, you know. I almost, I struggle to sort of do things to motivate myself to get work done, or you know, go out and do something. And I tend to sort of stop at home and make excuses to go out really, and that’s generally how it happens…” (p3,M,acu)
“I do get an awful lot of headaches, which I've attributed to the side issues… I have suffered from headaches all my life... Since I had cancer… my migraines have become 10, 20 , 30 times worse. And my headaches are 100 times worse... It's entirely possible...that it's linked to that (depression). But I couldn't just say for certain. I get terrible tension headaches, and I get bucket loads of back pain. It's a constant feature in my life, you know. Sadly it's probably what I would just term the norm these days.” (p12,M,acu)
“Very fitful. Sometimes - it seems to be on a cycle and I keep saying I'm going to write this cycle down. Where I can't get to sleep. It's once every so often.... Sometimes it's terrible getting to sleep. And then I start thinking, which isn't helpful.” (p8,F,acu)
“Some days I just think I don’t want to go out that door. I don’t want to talk to anybody.... I get a lot of fatigue anyway, so I’m tired and I haven’t got the energy to think some days.” (p10,F,acu)
“Everything around me was just black and negative... I would never allow myself go near that again.” (p13,M,acu)
“ I feel as if I’ve got a real heavy head, as if – not a headache as in a really painful headache, but it’s just like I could just go and sit in a dark room and shut away for a time, to clear my head. It’s just like it’s a heavy feeling” (p21,F,acu)
“I also had a bowel disease called diverticulitis, and I had an operation and part of my bowel removed 2 years ago and that’s caused me problems obviously before then and since then, because they've now found that I've still got it but in another part of the bowel... I've had IBS for many years, about 15 years. And obviously when I get down and depressed, that really kicks in and the diverticular disease I've had pain all the time from that and that tends to be managed by sort of diet and medication... the likelihood is I'd end up with a colostomy, which I don't want. I'm trying to sort of manage the disease without having another operation... My IBS is certainly related to depression yeah.... When I was looking after the old dears, as I call them, I was offered an operation twice and I turned it down because I didn't want to be, you know, incapacitated and not able to look after them. Once they passed on, I was then able to address my own health problems.” (p26,M,coun)
I find that depends on my state of mind. How the day has been. If I’m feeling kind of happy and content with everything, then usually I get a full night’s sleep. And if not, then it’ll be up every couple of hours, just waking up for no apparent reason whatsoever...” (p28,M,coun)
“Everything’s such an effort... At one point I didn’t even get out of bed. I didn’t even want to get out of bed. I’d sort the children out and then I’d just go back to bed because it just hurt so much.... before I received treatment and before I was diagnosed with post-traumatic stress, you know, you’d get them off to school and I’d just left work at that point, and I would go back to bed and do nothing...” (p32,F,coun)
“at the time when things were bad I would get a lot of stomach, sort of indigestion type pain. But that was at the time when we were really sort of dealing with the sort of intensity of it…”(p38,M,coun)
“the times when I don’t feel good, my usual pattern would be to wake up like 4.00 am, something like that... well some nights having trouble getting to sleep, but more waking up and then not being able to get back to sleep” (p44,F,coun)
“I’d lost so much weight as well. I’ve never been an overweight person, but I’ve always kind of fluctuated between a 10 and a 12. But the amount of weight that I had lost, just in the sort of like 2 or 3 months, was really drastic, you know, the clothes were hanging off and I looked very gaunt in my face.”(p46,F,coun)
“I didn’t want – there were days when I just didn’t want to go out of the house.... I was just constantly tired... there were days where I would get my little girl off to school and then come home and go to bed, and do very little before I had to go and pick her up.” (p50,F,uc)
“If things aren't going too well generally here, then the arthritis seems worse. But whether that is psychosomatic or not I don’t know I really don’t know. ... one of the things I did say to the psychologist, and as bonkers as that sounds is, it’s almost like the arthritis is punishing me for something I’ve done wrong.” (p52,F,uc)
“I have got a lot of nightmares actually, that tend to wake me up... about three o’ clock, something like that. It’s a bit better now, but it did take me a long while to get back off to sleep. It wasn’t as if I woke up and nodded back off again. I could be awake for a good hour... just wanted to turn my head off.”(p57,F,uc)
“it’s stopped me doing things I used to enjoy doing. I’m not – it’s made me tired a lot of the time. Agitated, grumpy, short-tempered... I used to do a lot of reading, which I hardly do any reading now. I used to go out on bike rides and I don’t do that anymore. I used to go swimming; I stopped doing that... I get tired a lot.” (p58,M,uc)

For peer review only

Supplement 5. Contextual factors
"I can't cope with bullying at all. I seemed to always get picked on. And I'd just end up getting depressed... Well it always seemed to be a friend, they'd pick on you. You know, they'd always make you feel a fool and – it wasn't actually bullying like thumping bullying. Just make you feel out of it… And it always seemed to have happened. So I sort of won't get close to anybody anymore." (p6,F,acu)
"My first husband died. My father died. And then everything's my fault then, you see. And I'm the one in the family that, if anything goes wrong, everybody comes to me."(p8,Facu)
"I've always had a tendency to go into waves of depression throughout my life.... It's a periodic thing. It comes and it's very difficult to know why it comes. But when it comes you really hit the trough and you go down and it's very hard to pick yourself up... It makes me extremely moody. It makes me extremely irritable. It makes me not enjoy anything about me. She (mother) was in and out of many mental institutions. And it created a great deal of difficulty within my life… It did affect me and made me very introverted."(p12,M,acu)
"I'd got myself tied up into...over-valuing myself in relation to work.. I just started increasingly shutting people out that would try to get close to me. And I think that's probably because deep down I was fundamentally insecure and my kind of work outlet was my bit of success that was under my control... I'd just lost sight of things that were important." (p13,M,acu)
"I felt I aimed too high and then I didn't sort of reach what I aimed to do. So I beat myself up a bit about it."(p20,F,acu)
"It's predominantly an anxiety disorder and I think that then triggered the depression. It varies for no apparent reason whatsoever. It can be quite kind of crippling social anxiety sometimes and at other times it can be a kind of general waiting for something to go wrong; the kind of belief that there's always going to be something that's going to go wrong. And it just gets worse and worse and worse and I think it went on for such a long time that it just became how things are. That was just how my life was. Just waiting for something else to go wrong and being petrified of everything." (p28,M coun)
"I've been unemployed since November. I was employed. I left my work to look after my brother, who was poorly. And he died in November. Well I was hoping I was going to be a full-time carer for a lot longer than what it was, but it was only a matter of a few weeks, really."(p29,M,coun)
"I'm too sensitive. I believe what people say. I'm very affected by what people think about me... whether it's where we live, or the close knit community that there is here, I still feel that we're outsiders... Whether it's we give off this aura that we don't belong or because we don't do things the way they should be done around here, I don't know. But we're still not part of anything. And we've been here 8 years. It's strange... I don't know why, apart from the fact I wasn't born here."(p35,F,coun)
"I'm doing very well... But I struggle sometimes with the workload and controlling my emotions and things... Generally when I'm working, I'm a lot better. Because I like to be on the go, I like to be doing things, I like to be learning things. But I struggle though, because I'm dyslexic, and academic stuff really stresses me out. I think that's probably why I've been getting worse over the last month, because my deadline for my portfolio is tomorrow. And I know I'm worked up about that. And I've got an exam in May that I'm worked up about... at the moment I'm actually just purely overloaded."(p43,F,coun)
"I have had a job that's been pretty full on since December which has just finished. So all my energies went into that really. Which isn't healthy, I know, but I wanted to make a good job of the job, if you know what I mean."(p44,F,coun)
"I've lost a lot of confidence since I had the stroke and I'm a bit wary about going out in case in happens again and no-one's around, you know. I've lost all my confidence, really." (p51,F,uc)
"I've needed help for a long time really, but... I'm finding it really hard to deal with, you know. Letters arrive and I daren't answer letters and a lot of the times when the phone rings, I don't really want to answer the phone because, you know, there's somebody chasing me for money or threatening me with legal proceedings, or...."(p54,M,uc)
"I didn't sleep. I was very stressed and then I'd crash out as well. So I didn't sleep, I'd work really hard. I'd study really hard and then I'd crash.... I do set myself a high standard. But then I work to that standard but I don't push myself excessively to the point of collapse." (p55,F,uc)
"The way that it – the most debilitating manifestation for me I found myself spending the last three months living in one room in the house. And not going out. Escaping the world really...I had been depressed before and I kind of knew what I was getting myself into. And really just wanted to escape from there, but by the time I'd fully got myself round to wanting to tackle – it just slipped too far too quick and I couldn't actually cope on my own anymore."(p56,F,uc)
"About caring for ill spouse) it's stopped me doing things I used to enjoy doing. I'm not – it's made me tired a lot of the time. Agitated, grumpy, short-tempered. I used to do a lot of reading, which I hardly do any reading now. I used to go out on bike rides and I don't do that anymore. I used to go swimming; I stopped doing that. I get tired a lot.... I go to bed at night but a lot of the time I'm not asleep before 12 and I'm usually awake about six…I've been known to sit down in an afternoon, and I've been asleep for an hour." (p58,M,uc)

For peer review only

**Supplement 6. Experience of acupuncture treatment**
"Well, you're relaxing anyway, in as much as you're just lying there. ... You're talking to him and you're passing the time of day and you're relaxing while he's doing it… I didn't feel any different at all....You're lying down and my ankles, elbows, and things like that and I didn't see what he was actually doing. And I'm just lying there passing the time of day, if you like. And at the end of it, I must admit, I didn't feel any different whatsoever.... There was nothing there that was to dislike. As I say, it was just really, well, it was a morning out." (p2,M,acu)
"100 times better. Not that I dislike my GP, I think he's fantastic, but you know, I've had counselling over the years and a lot of the problem was I did child protection work for most of my working life and eventually I became so depressed with it and I think this, you know, it's not talking it's treatment you go and have done like a physical treatment. ... I think that's really positive. And a lot more positive than talking to your GP. I think you get to the stage where you've just had enough discussion with people. You just want to feel better.... It made me feel better almost straight away. Whereas talking is a lot more emotional. It's taking a lot more emotion from you and it's also making you think and do things. Sometimes you don't want to do that, you just want to feel better."(p3,M,acu)
"I'm crippled with arthritis. I walk with a walking aid... It was the acupuncture, which was really good. And I really felt it helped. You know, and it also seemed to be doing my back pain good."(p6,F,acu)
"The very first session I had, I came home and I was utterly exhausted, for days... I just couldn't move one foot in front of another. It was really quite bizarre and I said to my husband, if this is what's going to happen every week, I really don't want to do this... I went back and I said, and she was lovely, because I think at first she thought I was not going to come any more. And I said, it made me so tired, please is there something you can do. And she said, oh yes, you could adjust – I don't know, whatever it was she could adjust – and after that I never felt that wretched again. I was absolutely fine... But as soon as I told her and she adjusted things it's never happened again." (p7,F,acu)
"I found that quite from the outset, you know, I soon got to think, oh, I think I'm feeling better. Quite quickly... with some people it works and I was getting a lot from it, while I was having it.... It was absolutely fine for maybe a couple of days, I felt, oh I'm getting better. But then as I stopped it, I thought, I don't feel -- for me, I think it would have to be an ongoing thing."(p8,F,acu)
"It has helped me realise now when I'm getting depressed. It's just something trivial that's set me off and it's not such a big deal after all. I can work around that. And I think without the trial, I don't think I'd be where I am now." (p10,F,acu)
"I found I was coming out of the...building -- I don't know what you'd call it, in a more upbeat and elated manner. So for me personally, I have to say that I found the acupuncture to be a very positive thing in my life. It was something that I found made me feel an awful lot better in myself.... You have to ask yourself the question, does acupuncture work as a form of medicine. Or does some people fussing about your body give you a feel good factor?... I can't answer that... But I would say that I found acupuncture to be very positive in my life."(p12,M,acu)
"The actual process itself wasn't at all painful. Occasionally she'd hit a sort of - a stinging pain – which she always said was something to do with the meridian or some acupuncturic explanation... Walking home from it a couple of times I did feel a bit, I won't say light headed, but slightly different." (p14,Macu)
"It was quite relaxing when I was having the treatment, to be honest, which I didn't think it would be. But once I got used to it, and once the needles went in and she left me, that was my time to … that was my time to relax... I used to look forward to going." (p17,F,acu)
"It wasn't an explanation that made much sense to me in that it was about things like holding grief in your lungs or something -- what on earth does that mean? …..It just sounded like new-age nonsense, to be honest. It sounded unscientific, which I find very off-putting. You know, we need to respect the scientific method, it's how knowledge is acquired and it seemed to me that it wasn't expressed in terms that I could relate to in that kind of way.... on a kind of broader level it did seem to verge on the snake oil." (p19,F,acu)
"as the treatments went on, she sort of tried to give me more energy with different treatments. But then I was having problems with skin rashes and anxiety and things like that, so on other weeks she would treat me differently, depending on what happened that week, whether I'd been feeling more anxious or less anxious or more lethargic.... and she linked it to my menstrual cycle as well. Because when I was coming back in saying that I'd had a particularly bad week, she noticed before I did that that was probably PMS, which sounded really basic. I felt like a right idiot at the time, but obviously that kind of helped me at least find a reason for why..." (p20,F,acu)
"I think that made a big difference because I knew I could feel the treatment instantly. Once the needles were in I could feel it, so I think that really sort of got me feeling positive because I felt as if it was doing me something good within my body. It was really acting for me and working for me... I did feel different and I did feel then as if I wanted to go – you know, the more I'd been the more I wanted to go and have another session!" (p21,F,acu)

For peer review only

**Supplement 7. Experiences of counselling.**
"He was talking about what triggers my major depressive episodes, and it was things like, he was talking about how to recognize the small things that then build into the big things, and how to deal with them and how to recognize them and how to put in place a system of recognizing what I can control and recognizing what I can't. So it was useful in that respect." (p25,M,coun)
"I suppose that I would have liked her to have been a bit tougher. And sort of ask deeper questions, probably. It's always quite awkward I think, because you go there and you know, it's -- and you've got someone sitting opposite you smiling and thinking, well go on, say something, say something. And they're waiting for you to say something. But you don't know what to say. So it felt a bit strange." (p26,M,coun)
"I felt as if I could talk to her openly and maybe try to get to the bottom of things. Just to see if there is something that could get me on the right road sort of thing... I feel as if it did help the first few months. And then things just steadily over the weeks got back to how it was before…when I had my counselling, I'm sure for two or three months afterwards I felt a lot more positive. I was living in my other house then. I was living in H where the town's quite busy. And I'm sure I was doing more and forcing myself to do more... I remember her saying that you've got to try and be more positive and try to take every day as it comes like and try to do different things. And I think it. I know in my mind what I should be doing. But it's just doing it... I sometimes listen to the radio. But it's usually the television if I'm not sleeping. And the television's just on. I'm not particularly watching anything – it's just on." (p29,M,coun)
"Now I'm happy to put my proper clothes on that match -- as opposed to track suit bottoms and a really grotty t shirt, and not do my makeup and not do my hair. Whereas now I wouldn't dream of going out without having done my hair or my makeup. You know, I've always had my hair short, but I was letting it grow long, because I couldn't even face going to the hairdressers -- that would have been such a challenge." (p32,F,coun)
"She said I was a giver.... which means you give to everybody, but then you don't expect anything back. But she said everybody needs to have something back. You can't keep on giving and giving, because she said, you'll burn yourself out and that's not fair on you, she said, or on your family... [She] would say to me...it's all fallen back on you, hasn't it?... I only live down the road from my parents, and I can't see them struggling... They know that at the end of the day, it's me who's the reliable one, if you like.... She said, why should you have to be the one who's always doing that. Or who's expected to do it."(p33,F,coun)
"Difficult to say… external changes made things a lot easier… a second job... a bit more money... the holiday abroad booked... it was difficult to tell whether it was her counselling that had helped me or the changes in our circumstances that had improved."(p35,F,coun)
"I thought it was helpful. At the time I was on a bit of an even keel, so I didn't really need it that much. But, you know, I still had problems and everything, so I found it really helpful just getting to know my own traits a bit more so that when I do have bad episodes... I don't think I really have changed. I think I feel a bit more insightful into things and maybe a bit more wise... Talking about your life and you know, it's not like he told me what to do and how to feel about things, but just asked the right questions and get you to talk things out in your own head. It's almost like you're having a conversation with yourself, it gives you the right...to think about the right things." (p36,F,coun)
"And I thought well what, you know, what is this all about. I'm not getting anywhere. And then either she said something or I said something and it twigged... And I said to her, I said, it's like defragging a computer. And putting that stuff into little boxes, or into – and chucking out the stuff that the mind or the computer doesn't want. And as soon as I said that, things started to click into place…I don't know whether it was through trust probably, and seeing the right person, I was able to download, or offload all those things to L and she put it into perspective." (p37,M,coun)
"Before, I'd given up on life and I couldn't see a way forward. What was the point? Because I was physically unable to do anything I wanted to do. But now I look at it more positive." (p42,F,coun)
"I think I'm doing all right. I know I'm hard on myself. I'm very hard on myself... but I'm not doing it consciously. I'm doing it unconsciously. So that is harder to control. Because I'm making decisions on something and not really thinking, I'm just doing it. And thinking, yeah, I can do this. And maybe I don't even know what I'm doing to set the bar... I'm setting the bar and not really thinking about it." (p43,F,coun)
"I think my understanding of myself has changed beyond recognition. Yeah. I would say I'm a lot more self-aware and a lot more – you know, I do understand myself a lot better than I did a year ago. Not only that, I actually accept myself a lot more than I did a year ago. I can actually say, you know, this is me." (p45,F,coun)
"I don't know, she sort of – you know when somebody says something and it just actually resonates. And it's a much more real experience. Things click into place, somehow... You know, just putting it into a visual – just in a way, I don't know, something just clicked. Something that I understood. I think they sort of tapped more into me as a person, and I wasn't just being talked at or... Because sometimes you can talk and you know that you're not connecting on any level and they don't understand you and they're not actually really listening to what you're saying." (p44,F,coun)
"the counselling that I received and my domestic violence support, helped me to understand why I was feeling the way that I was and obviously to, you know, talk about the things that I was experiencing. And get it all off my chest. I'm in a much better place now... They listened. I didn't realize that I needed somebody to talk to.... So having somebody outside of that bubble.... if they needed to give me advice, then a professional, experienced opinion was easier and it would come across differently as well. And they could listen without getting upset... I think it was just having that outside person to possibly shed a tear with, who was not emotionally involved." (p46,F,coun)

For peer review only

**Supplement 8. Experiences of usual care.**
"I'm on antidepressants and I sort of go through good periods and bad periods and when I'm in a good period I try to come off the tablets, because I don't want to be dependent on them. But I realise that by coming off the tablets, I needed them. And I had to go back onto them. And because I'm so worried about being dependent on them, you know.... there doesn't seem to be any help out there, other than some tablets that will just keep me on an even keel. And sometimes the tablets even when I'm happy don't even allow me to be as happy as I should be... Yeah, it's like you're just sort of on one level all the time. Yeah, you're not depressed and you're not anxious, but you just don't get any excitement or, you know, at times when you should really. And it's really weird. A weird thing really. It's really hard to describe. You just can't seem to muster up any enthusiasm... I was more concerned about my anxiety. I asked if there was any kind of drug that I could have just to control my anxiousness, you know. Something instantly, you know, when I could feel myself getting anxious. But there was nothing that they could prescribe for me... at the moment I just feel like I've reached a dead end. There's nowhere else for me to go and nobody's going to make me any better, other than continually giving me these drugs. There's no treatment for me. There's no treatment for me unless I pay for it." (p50,F,uc)
"It's kind of got to a stalemate really. I think that's the way of it. I was trying to think about this, really. I think one of the things for me that kind of was a problem, if you'd want to call it that, was now everything's on the repeat prescription thing, normally with my former doctor, you'd go once a month or whatever, just to get a repeat prescription. And that 10 minutes was kind of just enough to almost keep you going. And my old doctor I'd known for a long time. The new one, maybe only six years or so, but now because everything's on repeat, I don't think I saw her at all last year, because you just don't need to... I normally do try and see her because obviously, you know, it's easier in a way if you do see the same person, for them as well... the GPs just don't have time, I mean, you're there 10 minutes; there's nothing much you can say, really. But I think that certainly for me, that not going regularly hasn't helped... But unfortunately now, whatever care there is out there, it's rationed – not rationed, it's just that the time and the cost and you know – I think that's just one of those things, isn't it, really."(p52,F,uc)
"I've got fluoxetine tablets and they seem to have helped a bit, because I mean before I started taking them I was suicidal – I used to think about it quite a lot... supposed to take one a day, but I take one every other day. And it seems to have taken the edge off the panic. I don't pace around the house as much as I used to do.... I worry about side effects. I'm on loads of other medication as well for... various other bits and pieces... It's not that I'm worried about getting addicted to them. I just don't want to be – I don't want to take the edge off too much, you know what I mean... I'm still really depressed and I still think about suicide a lot. The mess I'm in at the moment is just unbelievable. It would take me all day to explain the mess I'm in... it's just the people that it would upset if I did, you know. Like my son. And my dad. My dad's not well and I don't want to upset him either." (p54,M,uc)
"What he wanted to do was refer me to this, I think it was a link worker was what he said, and I went, and he said it will take a few months and you'll get your appointment... I have to be honest, the doctor that I saw... he's amazing. He is a particularly good doctor. He was actually the one that helped me with my fibre intolerance. It was always brushed off by other doctors and he realized that it was a real struggle for me and I was in a lot of pain. And with this as well, he was – he actually sat and listened and understood. And then he said, look I'll recommend you, but there is a long wait. And I'll put you on these tablets to tide you over, but I don't think it's for you. And I said, well neither do I, but I can't – and I don't know if it would have happened with any other doctor. How long I would have to have waited." (p55,F,uc)
"I have seen a counsellor but I didn't really find it beneficial... but I decided there and then that I didn't want to pursue it. You know, I find sometimes that they talk in like, clichés. Do you know what I mean?... I think she was fairly experienced. She had like lots of candles and she had lots of alternative things, you know. Yeah, but I just felt, oh, I think we're talking in clichés here. And it's not really helping."(p56,F,uc)
"I was referred to a counsellor last August, and it took six months for me to get the appointment through... And I had two sessions with this counsellor (link worker) and I was starting to feel a lot better and she said that I didn't need to see her any-more and that I was managing to cope on my own, so it was really quick, but I did feel really good actually at the time... I had a couple of sessions with her. And I did feel really good. I felt really in control and everything... It just took an awfully long time to come through. And I could really have done with it at the time, rather than having to wait six months... You really need somebody to help you, well more or less straight away. A couple of weeks really. That was the problem with it."(p57,F,uc)
"The GP was very, very good. Recognised quite early on what was happening and basically put me on some antidepressants which were nominal. They didn't really stop a downward slide but as she progressed – I mean she couldn't actually refer me to anything straight away – she just wanted to make sure she was right... But she eventually said, you know exactly what you're doing so you can regulate your own medication – it's there, whatever you need at the time. Obviously if I need a review I go every six months or whatever for a complete review. And I can't change medication myself, I can't kind of prescribe myself something new, but the medication that I do take is available. It's there on the... I can just go, I need some of that, and if it's there I can have it. She trusts me to actually look after myself as it were. To draw up my own strategies for survival, if you like."(p59,M,uc)

For peer review only

Consolidated criteria for reporting qualitative studies (COREQ): 32-item checklist

Developed from:
Tong A, Sainsbury P, Craig J. Consolidated criteria for reporting qualitative research (COREQ): a 32-item checklist for interviews and focus groups. *International Journal for Quality in Health Care*. 2007. Volume 19, Number 6: pp. 349 – 357

YOU MUST PROVIDE A RESPONSE FOR ALL ITEMS. ENTER N/A IF NOT APPLICABLE

No. Item	Guide questions/description	Reported on Page #
**Domain 1: Research team and reflexivity**		
Personal Characteristics		
1. Inter viewer/facilitator	Which author/s conducted the inter view or focus group?	Methods
2. Credentials	What were the researcher's credentials? E.g. PhD, MD	Contributors section
3. Occupation	What was their occupation at the time of the study?	Contributors section
4. Gender	Was the researcher male or female?	Contributors section
5. Experience and training	What experience or training did the researcher have?	Contributors section
Relationship with participants		
6. Relationship established	Was a relationship established prior to study commencement?	Methods
7. Participant knowledge of the interviewer	What did the participants know about the researcher? e.g. personal goals, reasons for doing the research	Methods
8. Interviewer characteristics	What characteristics were reported about the inter viewer/facilitator? e.g. Bias, assumptions, reasons and interests in the research topic	Contributors section
**Domain 2: study design**		
Theoretical framework		
9. Methodological orientation and Theory	What methodological orientation was stated to underpin the study? e.g. grounded theory, discourse analysis, ethnography, phenomenology, content analysis	Methods
Participant selection		
10. Sampling	How were participants selected? e.g. purposive, convenience, consecutive, snowball	Methods
11. Method of approach	How were participants approached? e.g. face-to-face, telephone, mail, email	Methods

For peer review only

12. Sample size	How many participants were in the study?	Results
13. Non-participation	How many people refused to participate or dropped out? Reasons?	Results
Setting		
14. Setting of data collection	Where was the data collected? e.g. home, clinic, workplace	Methods
15. Presence of non-participants	Was anyone else present besides the participants and researchers?	Methods
16. Description of sample	What are the important characteristics of the sample? e.g. demographic data, date	Results
Data collection		
17. Interview guide	Were questions, prompts, guides provided by the authors? Was it pilot tested?	Methods
18. Repeat interviews	Were repeat inter views carried out? If yes, how many?	N/A Methods
19. Audio/visual recording	Did the research use audio or visual recording to collect the data?	Methods
20. Field notes	Were field notes made during and/or after the inter view or focus group?	Methods
21. Duration	What was the duration of the inter views or focus group?	Methods
22. Data saturation	Was data saturation discussed?	Methods
23. Transcripts returned	Were transcripts returned to participants for comment and/or correction?	N/A Methods
**Domain 3: analysis and findings**		
Data analysis		
24. Number of data coders	How many data coders coded the data?	Methods
25. Description of the coding tree	Did authors provide a description of the coding tree?	Methods
26. Derivation of themes	Were themes identified in advance or derived from the data?	Methods
27. Software	What software, if applicable, was used to manage the data?	N/A Methods
28. Participant checking	Did participants provide feedback on the findings?	Methods
Reporting		
29. Quotations presented	Were participant quotations presented to illustrate the themes/findings? Was each quotation identified? e.g. participant number	Results & Supplements 4-8
30. Data and findings consistent	Was there consistency between the data presented and the findings?	Relationship to existing knowledge
31. Clarity of major themes	Were major themes clearly presented in the findings?	Results
32. Clarity of minor themes	Is there a description of diverse cases or discussion of minor themes?	Results Negative views presented

For peer review only

BMJ Open

Patients' experiences of acupuncture and counselling for depression and comorbid pain: a qualitative study nested within a randomised controlled trial.

Journal:	*BMJ Open*
Manuscript ID:	bmjopen-2014-005144.R1
Article Type:	Research
Date Submitted by the Author:	19-May-2014
Complete List of Authors:	Hopton, Ann; Universiy of York, Health Sciences<br>Eldred, Janet; University of York, Health Sciences<br>MacPherson, Hugh; University of York, Health Sciences
**Primary Subject Heading**:	Complementary medicine
Secondary Subject Heading:	Qualitative research, Patient-centred medicine, Mental health
Keywords:	Depression, Pain management , PRIMARY CARE, MENTAL HEALTH

SCHOLARONE™
Manuscripts

For peer review only

TITLE: **Patients' experiences of acupuncture and counselling for depression and comorbid pain: a qualitative study nested within a randomised controlled trial.**

Ann Hopton[1], Janet Eldred[1,] Hugh MacPherson[1]

[1] *Department of Health Sciences, University of York, UK*

Keywords; depression, comorbid pain, primary care, acupuncture, humanistic counselling

Address for correspondence:

Ann Hopton
Research Fellow
Department of Health Sciences
University of York
Heslington
York YO10 5DD
United Kingdom
Tel: 01904 321384
E-mail: ann.hopton@york.ac.uk

For peer review only

ABSTRACT

Introduction

Depression and pain frequently occur together and impact on outcomes of existing treatment for depression. Additional treatment options are required.. This study aimed to explore patients' experiences of depression, the processes of change within acupuncture and counselling, and the elements that contributed to longer-term change.

Methods

In a sub-study nested within a randomised controlled trial of acupuncture or counselling compared to usual care alone for depression, semi-structured interviews of 52 purposively sampled participants were conducted and analysed using thematic analysis.

Results

Differences were reported by participants regarding their experience of depression with comorbid pain compared to depression alone. Along with physical symptoms often related to fatigue and sleep, participants with depression and comorbid pain generally had fewer internal and external resources available to manage their depression effectively. Those who had physical symptoms and were receiving acupuncture commonly reported that these were addressed as part of the treatment. For those receiving counselling, there was less emphasis on physical symptoms and more on help with gaining an understanding of themselves and their situation. Over the course of treatment, most participants in both groups reported receiving support to cope with depression and pain independently of treatment, with a focus on relevant lifestyle and behaviour changes. The establishment of a therapeutic relationship and their active engagement as participants were identified as important components of treatment.

Conclusion

Participants with and without comorbid pain received acupuncture or counselling for depression, and reported specific identifiable treatment effects. The therapeutic relationship and participants' active engagement in recovery may play distinct roles in driving long-term change. For patients who present with both depression and physical symptoms of care may wish to consider a short course of acupuncture to relieve symptoms prior to a referral for counselling if needed.

ISRCTN63787732

Strengths

- The 52 telephone interviews were obtained from a wide range of participants in socially diverse settings and provide rich data on the participants' experiences of depression and the treatment received in the ACUDep trial.
- The thematic analysis was conducted using a bottom-up process to allow the themes to develop directly from the participants' own words, and we present the positive and negative experiences of each form of treatment, whether treatment was beneficial or not.
- Our findings identify mechanisms within the processes of change that are specific to acupuncture and counselling that facilitate reduction in the symptoms of depression.

Limitations

- Participants may have attributed changes directly to treatment rather than concurrent, coincidental contextual changes.
- There is a possibility of recall bias; however, it is likely that the participants recalled the aspects of treatment that were most salient to them.
- Telephone interviews prevented the interviewer gathering non-verbal contextual information, although this form of interview was more acceptable to participants than a face-to-face interview.

For peer review only

INTRODUCTION

Chronic pain is commonplace in half to two-thirds of participants with major depressive disorder.[1][2] The association between pain and depression becomes stronger as the severity of either increases,[3][4] and the impact of both problems on each other plays an important role in the development and maintenance of chronicity in health problems.[5] Participants with depression and comorbid pain are difficult to diagnose, feel an increased burden of disease, tend to rely heavily on health care services and are more difficult to treat.[6] Identifying and managing the pain symptoms that commonly occur alongside symptoms of depression may be important in improving depression response and remission rates.[7] Previous qualitative research reports that patients with comorbid pain and depression identify the ineffectiveness of existing pain relief strategies, a lack of tailoring strategies to meet personal needs and difficulties with patient –physician interaction as barriers to the effective self-management of their symptoms.[8] Patients appreciate a health-care approach that is individualised [9]and are open to the value of complementary-health-care.[10] Two health-care options that offer these attributes are acupuncture and counselling.

There is a growing evidence base in support of the effectiveness of acupuncture for a range of musculoskeletal conditions;[11][12] however, despite its widespread use by participants[13] there has been limited evidence for acupuncture as an effective treatment option for depression.[14] Patients with strong preferences for psychotherapy or counselling for depression are not likely to engage in antidepressant treatment,[15] yet the evidence for counselling as a treatment for depression is limited[16] despite widespread utilisation in primary care in the UK, with around 90% of general practices providing on site counselling services.[17] To address this evidence gap, a randomised controlled trial (ACUDep) compared acupuncture or counselling to usual care as treatments for primary care patients with ongoing depression.[18] The results showed that both acupuncture and counselling were clinically effective in reducing depression in the short to medium term.

In a quantitative sub-study nested within this trial, which focused on the effect of comorbid pain on the outcome of treatment for depression,[19] it was found that approximately 50% of the ACUDep participants had co-morbid pain with depression and that the participants' pain scores at baseline predicted the outcome of treatment for depression at the three-month follow up point. A comparison of the treatments showed that patients with depression and comorbid pain had a better outcome in the short to medium-term with acupuncture.
The ACUDEP trial[18] was novel in its' investigation of acupuncture or counselling compared to usual care; yet in focusing on the clinical effectiveness of the treatments offered, the published results did not set out how the process of change occurred as a results of receiving the three treatments of acupuncture, counselling and usual care . To meet this evidence gap the aim of this qualitative study nested within the ACUDep trial, is to explore patients' reports of their experiences and identify from their reports how each intervention influences long-term change.

METHODS

Design

This research comprised a qualitative sub-study nested within a three-arm randomised controlled trial conducted between November 2009 and June 2012 of acupuncture or counselling provided as an adjunct to usual care compared to usual care alone alone.[18] In this nested qualitative sub-study, a purposive sampling frame was prepared to recruit interviewees in the same 2:2:1 proportion of the main trial, to include 50% males and 50% females in each arm and to balance whether in pain at baseline or not. The design used a constructivist approach to grounded theory[20] to ensure that the theories were constructed by the researchers as a result of their interaction with the area of the research and the participants.

For peer review only

Participants

Within the ACUDep trial,[18] 755 participants aged 18 and over with a history of on-going depression and score of 20 or more on the Beck Depression Inventory (BDI-II) were recruited by general practitioners at 27 primary care practices across Yorkshire, County Durham and Northumberland. Participants were allocated remotely by the York Trials Unit, with the allocation code concealed from the recruiting researcher, to three groups in the proportions of 2:2:1 to acupuncture, counselling and usual care alone respectively. Within the acupuncture arm, 23 acupuncturists registered with the British Acupuncture Council for a minimum of three years delivered up to 12 acupuncture treatments per patient, usually weekly. Treatments were tailored to individual participants' needs within a trial protocol,[21] which included treating symptoms according to traditional Chinese medicine theory and the integration of relevant life-style support into the treatment strategy based on acupuncture-specific advice if considered appropriate by the acupuncturists. Within the counselling arm, 37 counsellors registered with the British Association of Counselling and Psychotherapy provided up to 12 sessions of humanistic counselling delivered according to a trial protocol[22] which stated: "Counsellors will use empathy and advanced listening skills to help clients express feelings, clarify thoughts, and reframe difficulties, but they will not give advice or set homework." Deviations from the trial protocol were permissible if considered necessary and recorded in the participant's log book. Usual care comprised the treatment that patients typically receive in primary care along with over-the-counter medication. Commonly prescribed medication included anti-depressants and analgesics. Participants were followed up over a period of 12 months after randomisation by postal questionnaire at 3, 6, 9 and 12 months. On receipt of their final 12-month postal questionnaire and based on a sampling frame (see Supplement 1), a sample of participants who had previously consented to be contacted for an interview were invited to engage in a telephone interview of approximately 30 minutes duration. Altogether 52 people consented to a one-to-one, audio-recorded, semi-structured telephone interview for this study.

Interviews

A researcher (AH) interviewed all 52 participants. The researcher was unknown to the participants prior to the interview, therefore the interview opened with an introduction designed to set the participant at ease, to reveal the context for their depression, and to draw out the participant's account of treatment received as part of the trial. Prompts from a prepared topic guide were used to elicit the participants' experiences of depression and treatment. (See Supplement 2) The topic guide had been piloted previously and granted ethical approval by the University of York ethics committee.

All the interviews were conducted between February and May 2012. On average the interviews lasted approximately 25 minutes (range 11-46 minutes). Three participants were ill and felt fatigued, but were eager to participate; therefore exploration of the questions for these people was limited in time to prevent over-burdening them. To encourage participants to relax, each participant was asked to introduce themselves by speaking about things they like to do or hoped to do and then how depression had entered their lives, before moving on to the research-related questions within the topic guide. Interviews were audio-taped, transcribed verbatim and checked for accuracy. All recordings were of sufficient clarity and content that no repeat interviews were necessary. Each transcription was checked to remove any names and assigned a participant identification number.

Analytical methods

An inductive thematic analysis[23] was used to search across the dataset of 52 transcribed interviews. Using a constructivist approach to grounded theory,[20] data was analysed initially by AH by reading each transcript several times, then annotating ideas to generate a list of potential inductive codes developed from the dataset to capture and summarise the participants' experiences. Coding was performed sequentially on each transcript, initially without software, working

For peer review only

systematically throughout the entire dataset. As codes were identified, they were recorded and organised on an Excel spread-sheet; sections of text that demonstrated that code were then added and collated moving back and forth across the data set making comparisons with previous data in an iterative process.

Coding and extractions were checked by JE to verify that the participants' experiences were reflected and summarised accurately. As coding progressed, comparisons were made between codes and phrases and those with similar context or concepts were grouped together. This process was conducted within each interview and across interviews resulting in a codebook of 33 codes, seven subthemes and two themes associated with the experience of depression, (Table 1) and a codebook of 35 codes and four themes associated with the experience of the treatment; (Table2). Saturation for the main themes occurred within each treatment option within 5-7 interviews. The coding and identification of themes were discussed and developed throughout between AH, HM and JE.

For peer review only

Table 1. Coding tree; A summary of the relationships between codes, subthemes and themes for the experience of depression

CODES	SUBTHEMES	THEMES
Muscular pain		
Headaches		
Gastro-intestinal pain	Pain	
Disability		
Search for diagnosis		
Tired all the time		
Exhausted- worn out		
Debilitated	Fatigue	
No energy		Reported symptoms of depression
Withdrawal		
Insomnia		
Early wakening		
Nightmares	Sleep disorders	
Too much sleep		
Low self esteem		
What other people think		
Guilt and self-blame	Negative schema	
Black and white thinking		
Family life		
Motherhood		
Caring	Home life	
Bereavement		
Isolation		
Shift pattern		
Work overload	Work strain	Contextual factors
Drive, motivation		
High bar, aiming high		
Crime		
Bullied/ threat/ physical abuse	Victimhood	
Self-harm		
Childhood		

To understand the participants' individual experiences of depression and the treatments received, the codes and themes were developed into a diagram (see Supplement 3) and populated with participants' identity numbers. This enabled the researcher (AH) to trace each participant throughout the process and to make distinctions between those with depression and comorbid pain, and those with depression alone.

Throughout this paper the themes are illustrated with quotes that capture and embody the participants' experiences embedded within the analytical narrative as suggested by Braun and Clarke.[23] Additional illustrative quotes are set out in Supplements 4-8.

Table 2. Coding tree; A summary of the relationships between codes and themes for the positive and negative experience of treatment; factors that influence long term change and mediating factors

CODES		THEME
Beliefs and attitudes	Pre-treatment factors	
Previous experience of treatment		Mediating factors
Participant engagement	In treatment factors	
Therapeutic relationship		
Change of perspective	(common to all arms)	
Reduced medication		
Relaxation	(acupuncture)	
Complaints & symptoms treated		
Self-understanding	(counselling)	Positive experiences
Acceptance of situation		
Empowerment		
Effective medication	(usual care)	
Referral to NHS mental health services		
Adverse events	(acupuncture)	
Terminology		
Time consuming		
Fear of needles		
Difficulty opening up	(counselling)	
Clichéd phrases		Negative experiences
Opened a can of worms		
Expense		
Side effects of medication		
Long wait after referral	(usual care)	
Lack of general practitioner time and continuity		
Ageism		
Exercise advice		
Dietary advice		
Reduce alcohol intake	Lifestyle strategies	
Take time out		
Day to day structure		Long term behavioural change
Distraction		
Thinking strategies learned		
Self- talk & Stoicism	Cognitive strategies	
Social contact		
Seeking support		

For peer review only

RESULTS

Participants recruited

Of the 755 participants randomised in the ACUDep trial, 674 completed their 12-month follow up; 518 had consented to be interviewed and of these 518, 464 (89%) were potentially eligible to join the study. Of the 61 participants invited, four declined participation and three did not respond. A total of 52 participants, comprising 24 men, and 28 women with an age range of 22 to 89 years (mean 46 years, SD 13.8) were interviewed. At baseline, 26 of these participants had reported having moderate or extreme pain or discomfort on the EQ5D questionnaire; these people formed the pain group, the remainder formed the no-pain comparator group. As part of the ACUDep trial, 22 of the 52 had been randomised to receive acupuncture, 20 to counselling and 10 received usual-care alone. A summary is presented in the sampling frame in Supplement 1. On average, those allocated to acupuncture attended 11 sessions (range 4-12) and those allocated to counselling attended 10 sessions (range 6-12).

Symptoms experienced with depression and contextual factors

Participants reported a range of symptoms they experienced concurrently with depression: predominantly pain, fatigue and sleep disorders (see Supplements 3-4). Most participants with depression and comorbid pain suffered from either persistent headaches or moderate to extreme muscular-skeletal pain that predated the onset of depression and compromised their mobility, to the extent that four men had considered suicide, and one man had attempted suicide. Almost half of those in pain experienced sleep disturbances or an overwhelming loss of energy to the point where much of their time was spent in bed, withdrawing from social and day-to-day activities. (See Supplement 3)

> *"There are times when things like headaches and neck aches prevent sleep... I sort of drift 48 hours without sleeping... I have in the past been, if you like, down for a prolonged period of time...just sleep and sleep and sleep and sleep and wake up and do something for half an hour and then go back to sleep again. I can just about function with pain killers... The only time I did try to top myself that led to even more depression because I couldn't even do that right! I'd taken a boat load of the diazepam I was on, to try and calm me down. I'd sort of stock piled some of that…washed it all down with Glenfiddich, and instead of, you know, just shuffling off quietly, all I did was end up waking up feeling absolutely dreadful in a puddle of my own vomit, and it was one of those things where, you know, it took me weeks afterwards, thinking well, I can't even kill myself properly."*(p25,M,coun)

With regards to contextual factors, for the majority of the pain group, the pain they experienced had compromised their ability to work. Very few had social support, and for some, their being at home meant that they were the family member available to take on a caring role for a relative, which incurred further stress, ill health and isolation. (See supplement 5)

> *"Because I was off and not working I was able to have the time to look after my elderly mother and aunt who were both in their 80s. So I was their carer for 4 years and unfortunately I lost both of them and my father 4 years ago, within 6 months of each other...My IBS is certainly related to depression yeah...When I was looking after the old dears, as I call them, I was offered an operation twice and I turned it down because I didn't want to be, you know, incapacitated and not able to look after them. Once they passed on, I was then able to address my own health problems."* (p26,M,coun)

In summary, for the participants with depression and comorbid pain, the symptoms experienced impacted on the most basic level of physiological needs, and reduced their ability to engage in social

For peer review only

activity, whilst the contextual factors compromised their security through reduced income. Together these factors suggest that this group of people have few internal and external resources remaining to effectively manage their depression.

Of the participants who were pain-free at baseline, several people complained of tension headaches or gastro-intestinal symptoms they experienced at times of heightened stress and anxiety. Several others identified distinct patterns of disordered sleep: either difficulty in settling to sleep or a pattern of early wakening. Similar to the pain group, a few tended to withdraw at times when they felt particularly low in mood.

> *"I get a lot of stomach problems actually when I feel depressed...And of course, really tired as well – very, very tired when I'm feeling down…Sometimes I find it really hard to cope with people. I can possibly be a bit grumpy sometimes, or really quiet. Because I can't really face talking to anybody on certain days...I just can't bear it."* (p57,F,uc)

Regarding contextual factors, the majority in the no-pain comparator group were in full or part-time employment or were relatively affluent retired professional people. For many, their experience of depression concerned feelings of low self-esteem brought about by high expectations of themselves within their working life, or hectic social schedules. Others experienced low self-esteem and threats to their security from bullying at work or as a victim from domestic violence.

> *"We're also dance teachers, which is supposed to be a hobby but has somehow involved taking over our lives... we're teaching three nights a week, so it's a bit of a commitment for a hobby... Because when we started teaching last May time... that did give me more of an impetus to actually make the effort, because once you've got teaching you've got to go, because you're going to let people down. And I'm always glad that I do. If I'm having low days now, I'm always glad that I've been, because I'm concentrating on stuff that's completely outside of me or completely outside of my normal life... One of the things that's come through is that I hate letting people down. I'm very hard on myself."* (p45,F,coun)

> *"I tend to keep my things bottled up... And it really gets me down. My work suffers. My home life suffers. And everything suffers if I'm really bogged down with something….following a particular incident, or a series of incidents... I chose to or had to be the brunt for a lot of the aggression and violent behaviour that this man displayed at the time. So if you like, there was a particular traumatic... there was a starting point for it."* (p38,M,coun)

In general, the no-pain comparator group experienced fewer demands physiologically. Most had their basic security and social needs met; this group had larger reserves of both internal and external resources available to them to cope with their depression.

Processes of change reported by those receiving acupuncture: positive and negative experiences
The processes of change identified within the data formed three stages: primarily, developing a therapeutic relationship; secondly, the individual diagnosis and treatment of symptoms; and finally, engendering changes in health behaviours. Within the pain group and the no-pain comparator group, the positive experiences tended to facilitate the process of change at each stage, whilst negative experiences contributed to the barriers to change. Most participants welcomed the opportunity to try something different for their depression. The acupuncturists' understanding of their symptoms and explanations of how acupuncture might help their particular problems initiated the development of a therapeutic relationship. In contrast, two people described their acupuncturist as brisk, efficient and professional, yet lacking in bedside manner.

For peer review only

*"She was very positive about things...I think you have more of an intimate relationship with the person doing that rather than just a person in an office somewhere. You're physically involved." (*p11,M,acu)

"They could do with a course in empathy." (p22,F,acu).

Within the second stage of treatment, for most participants, the therapeutic relationship was further fostered through the acupuncturist listening to the participants' concerns, and treating the symptoms of depression depending on what was diagnosed to be of most importance to the participant at that point. Within the pain group, several people experienced relief from musculoskeletal pain which tended to last for a few hours or days after the session and improvement commonly built up over several sessions. Several also reported feeling deeply relaxed during the sessions and an uplifting sense of well-being afterwards.

"I thought it was quite a strange sort of feeling, but I sort of felt better quite quickly. And then I went to the second and the third, it was… it completely lifted my mood and it made me feel more motivated…It was almost as if a weight had been lifted off my head and all of a sudden I felt like some energy had come back." (p3,M,acu)

With regards to negative experiences, one woman reported extreme tiredness after the acupuncture session, a problem that was addressed by the acupuncturist by adjusting treatment during the next session, and one man with extreme back pain attributed needling pain to his damaged nerves from a previous injury. Both of these participants concluded that the treatment went well; however they also reported, *"It was not for me."* (p4,M, acu; p7,F,acu)

"she'd actually hit at the root for a problem that I have with my pain, because I think at one time she put a needle in me and I kicked her. Without wanting to I involuntarily kicked her. And she'd obviously hit upon -- I think there was one time when I kicked out and more than a few occasions where she'd twitched a nerve that obviously." (p4,M,acu)

In contrast, within the no-pain group two participants who were worried about the potential pain of needling prior to starting the treatment later attributed the needling sensations to the healing process. Three men thought they would have been equally relaxed if they had just rested or gone for a massage, and two other participants found the sessions too time-consuming.

"A little bit sceptical as to whether the treatments (acupuncture) work anyway. So it was for me like, if I go and get a sport massage, it was like the equivalent of that..." (p13,M,acu)

Factors that influenced long-term change reported by those receiving acupuncture.
As treatment progressed, many participants reported that their acupuncturist began guiding them to make changes to their lifestyle in order to engender beneficial long-term outcomes. For most people with pain, fear of pain and potential injury posed a barrier to engaging in physical activity. The majority of the pain group reported being encouraged to take up gentle exercise for their overall health and they also distracted themselves during periods of low mood.

"The things, they seem so small, but they are important. Things like, getting out and going for a walk and getting some fresh air. And just opening your eyes in the mornings and trying to cope with life." (p7,F,acu)

"He started about exercise, you know, how that can make you feel more up…" (p8,F, acu)

For peer review only

"I read a lot and try to keep my mind off it. Really." (p9,F,acu)

One man developed his own technique based on how he felt during the acupuncture to help him manage low moods, whilst another relied on monthly acupuncture treatments alone to stay well; another considered further treatments but found the cost prohibitive.

"I sort of developed this technique and I don't know, it was like… The way I was feeling during the acupuncture... I sort of clung on to this feeling that, or this technique of gaining that feeling, so I remember on a couple of occasions where I was out and about walking, and thinking about things that would normally would start leading me to start feeling a bit down, but it was like I'd been given this tool in my head and I just sort of – it just sort of went onto auto-pilot. It was like pulling those feelings away and just sort of throwing them away....Well, it lasted for a while but it started subsiding." (p1,M,acu)

"I go for acupuncture now once a month and I find that any more than a month and I can feel myself sort of slipping and feeling really, you know, starting to get worse again. And then I go and I feel much, much better…" (p3,M,acu)

The advice given to the no-pain comparator group was qualitatively different. Acupuncturists advised on dietary change, the reduction of caffeine and alcohol, and relaxation, which varied with the presenting symptoms and by gender. Those participants with least rapport also tended to be those who were less willing to make behavioural changes.

"She also helped with giving me other things that I can do. Suggesting different foods for me to eat to make me feel more energetic…. I was cold all the time and that made me feel more lethargic as well, because all I wanted to do was stay in and go to bed and stay warm. So she was suggesting that I literally ate warmer foods, and she gave me a list of sort of Chinese medicine sort of foods that they had." (p20,F,acu)

In general, the process of change evolved in three stages. The therapeutic relationship and active engagement in recovery acted as mediators of the outcome throughout each stage of the process. In the short term, acupuncture often relieved physiological symptoms of depression and of comorbid pain. Longer-term improvement in depression was developed through the participants' active engagement in health promoting behaviours, supported by a positive therapeutic relationship. Several participants with comorbid pain had less physical ability to engage in lifestyle changes and tended to be the passive recipients of care. These participants often had fewer external resources in the form of finance and social contact to manage their depression and comorbid symptoms in the longer-term. Additional quotes are presented in Supplement 6.

Processes of change reported by those receiving counselling: positive and negative experiences
Based on their previous experiences of counselling the majority of participants spoke of their low expectations of counselling. However when engaged with the counselling process within the trial, most reported being relieved to have someone to talk to in confidence. For both the pain and the no-pain groups, the process of change followed a common pathway: beginning with the participants' disclosure of personal information and being listened to.

"I found that process to be very valuable…I found X was very much listening and empathising, but maybe offered interpretation a bit more than the National Health person." (p37,M,coun)

For peer review only

For most participants this two-way active engagement appeared to nurture a therapeutic relationship between the participant and counsellor. Four male participants welcomed the opportunity to speak to a male counsellor, a choice which put them at their ease, and facilitated the process. Three others found difficulty engaging with their counsellor and attributed this problem to a personality clash. This presented an early barrier to the process of change.

> *"A lot of it does depend on who the counsellor is…I'm saying probably same sex works better. They probably have a clearer understanding of the male mind... I found him particularly sympathetic and, you know, very constructive. I think that was...I was very pleased with the way it went..."* (p40,M,coun)

> *"Every single counselling cliché that you have about, oh it's parenting issues – she kind of wheeled them all out one after the other and they were already things that I'd thought about, considered and looked at and examined to the nth degree and then thought, no that's not the problem... It felt like she was reading a script almost – like a guidebook to deal with this kind of disorder...it was almost the complete opposite of what I felt like I needed."* (p28,M,coun)

A second stage of the process of change was often identified as occurring around midway within the course of treatment. The iterative process of participants' disclosure continued, with deeper exploration of their past, which helped to clarify the participant's understanding of themselves and their situation.

> *"At the end of it, it actually for me opened up a can of worms really, and I think it did me more harm than good. A lot of my problems, especially with low self-esteem, come from the way I was brought up by my parents and my father especially. And its stuff that I'd never addressed and it brought it all out, actually. And I actually felt worse at the end of it... I don't feel so bad about it now, because I recognise why I am like I am and some of the problems I have, where they come from... Although I say they'd opened up a can of worms, and brought some upsetting experiences back... I think it was good to do that. Because those sort of things had been bottled up for many, many years... it's actually made me address them."* (p26,M,coun)

> *"It made me realize that I just held everything in. From being a little girl, everything that had ever bothered me it was never talked about. You know, I'm quite lucky that I've never had any real abuse or anything like that. It's just that I've got memories of being a child and things were said and it hurt. And I just locked it away. And I did that for years."* (p41,F,coun)

Several participants realized what factors triggered and perpetuated negative thoughts, some of which were unfounded. For one man with chronic pain, this meant going through a grieving process for the loss of his former way of life before setting in place new ways of thinking and coping.

> *"You know, I think that was the big thing that I got from it, you know – that I could see myself more positively after having the time with him. And understand that some of the negative thoughts that were coming to my mind were not reality, if you like. To let them sail past and focus on the good things that I've done in the past."* (p38,M,coun)

> *"Everything that defined what I was has now gone. And it took an awful lot of grief, if you like, to come round to the fact that it was worth trying again".* (p25,M,coun)

For peer review only

The use of metaphors was particularly useful for de-cluttering unnecessary thoughts about their past, regaining perspective, setting their problems into context, and focussing on what was important.

> *" the discussions were much more free than I'd kind of anticipated they might have been, was using metaphors and analogies and stuff like that, to be able to describe things and move through things. And the pictures were just coming to me in my head, like. I had one which was sort of like a circuit board and it felt like some of the wires were not quite wired up properly and they weren't working and stuff like that. And I can kind of track the metaphors throughout the whole process and it feels like it was much more of a – like it all opened up."* (p38,M,coun)

Factors that influenced long term change reported by those receiving counselling

The final stage in the process of change was directed towards enabling the participants to maintain progress independently. Gender differences became apparent in the coping strategies adopted; the majority of women took up health and well-being strategies. Compared to the women in the pain group, the women in the no-pain comparator group were able to use a wider range of resources to cope with, and engage in social activities more easily. One woman recalls being given cognitive behavioural homework to overcome a particular anxiety.

> *"She gave me sort of little exercises. I found it very difficult to walk down to a friend of mine. She lives in quite a built up area... People were sitting out in their gardens and I found it very intimidating. I didn't like it. I'd become really sweaty, short of breath walking down through her estate to go and see hear... basically she just taught me to get a grip on myself really, by pointing out, you know, that everything was going to be all right... short sharp steps really. And that I'd got the coping mechanisms and I could do it."* (p32,F,coun)

Many male participants appear to have continued to practise the cognitive strategies learned within the earlier sessions, and applied them to their life outside the sessions. However, male participants with depression and comorbid pain found greater difficulty sustaining these strategies and returned to their general practitioner for further help.

In summary, the majority of participants had had previous experience of counselling; however, their initial low expectations of success receded as the course of treatment progressed. A few counsellors practiced a more directive intervention than humanistic counselling, according to the need of the participant. The process of change comprised three stages, each mediated by the quality of the relationship and the participant's active engagement. Additional quotes are presented in Supplement 7.

Processes of change reported by those receiving usual care

Participants in all three arms received usual care throughout the trial. The process of change within usual care was less evident. Differences in the appraisal of general practitioner care were apparent: three older participants with depression and comorbid pain who were allocated to usual care alone complained of a lack of understanding and continuity of general practitioner and they felt abandoned without hope. One 89-year-old lady reported:

> *"I would never go to a doctor again. I am, because I suffer a lot of pain that I needn't have done if he'd been different…if he'd have listened to me instead of just pooh-poohing it off and saying, oh no, it's not that. If he'd have really listened to what I was saying, he could have done more for me...he's ignored what I've told him. Well, in fact he's very often just ignored it all together. Pretended I hadn't said it... You see, at my age you can't really change doctors. There's not many doctors want to take somebody on that's 90 years old, do they?*

For peer review only

When I'm having a really bad day... you know, and I feel I can't turn even to the doctors, you know, then yeah, I do get depressed." (p51,F,uc)

In contrast, the majority of participants who were pain free pointed to a relationship based on trust as being a component of their steady improvement over time. For most people, a regular monthly 10-minute consultation was helpful and constructive. A few felt that their general practitioner had made additional time for them when they were most in need.

"He sees me every month. I have monthly meetings with him just to have a general chat about how things are... I get on fine with him. As I say, I can talk about just about anything with him, so I suppose in a way, he's sort of, if anything he's been maybe a counsellor for me, because, you know, I can sit and talk to him about stuff..." (p58,M,uc)

All participants at some time had been prescribed antidepressant medication. The majority of participants on long-term antidepressant medication raised concerns about the side effects; participants in the pain group were particularly concerned about the potential effects of mixing medication for their other medical problems with their antidepressants. A few acknowledged that they needed antidepressants to maintain long-term stability.

"Staying on medication, it has transformed my life and made everybody else's life around me better as well. And I wish I'd have done it sooner... It must be four or five years now and yes, it's been life transforming... When I started on my medication and I could realize the difference – the two people I was, almost." (p7,F,acu)

In addition to medication, during the period of the trial general practitioners referred participants to a range of secondary services: two young women received three sessions with a mental health link worker, an intervention which had enabled them regain control of their lives; three others had been advised to try online cognitive behavioural therapy which two found to be easily accessible and effective; one man at risk of suicide was referred for urgent psychiatric help.

"I was referred rapidly to A&E and was assessed by X the psychiatrist. And I was put on to intensive home treatment. Which was invaluable. As a condition of not being sectioned..." (p59,M,uc)

Referrals for younger patients had been beneficial although the waiting times were long and not always found to be acceptable, leaving most patients without adequate support in a time of crisis, and without sufficient money to pay for private care.

"I go to the doctors and I have to wait a matter of I don't know how many months before I can get, you know, onto the counselling and you know, it's just like the moment's gone, sort of thing... Unfortunately that can cost a lot of money – I'm on benefit. I can't afford it...I don't hold out much hope." (p50,F,uc)

Factors that influenced long term change reported by those receiving usual care: positive and negative experiences

For those who received help via a referral, the advice followed a familiar pattern: to engage in lifestyle changes, to add structure to the daily routine, and to use distraction to reduce the focus on the symptoms and negative feelings. However, without support the stoicism of 'forcing myself' was a prominent default strategy among most usual care participants.

"I force myself to do things and then I generally feel better" (p57,F,uc)

For peer review only - http://bmjopen.bmj.com/site/about/guidelines.xhtml

"It's forcing myself. Well, it's a survival strategy..."(p59,M,uc)

Overall, the continuity of always seeing the same general practitioner was reported to be important and beneficial. By contrast, some older participants with depression and comorbid pain remained caught in a seemingly hopeless cycle of seeking diagnosis and treatment for a physical complaint and without resources to seek private health care services. Most patients who received acupuncture or counselling were also happy with the attention from their general practitioner, but had welcomed the additional treatment provided within the trial as an adjunct to their usual care. Supplement 8 presents a number of representative quotes.

DISCUSSION

Principal findings

The participants' experiences of depression were a complex interplay of internal and contextual factors. Compared to participants with depression alone, participants with depression and comorbid pain had fewer internal and external resources available to effectively manage their depression in the longer-term. Acupuncture and counselling treatments were individualised interventions that operated from different perspectives. Acupuncturists appeared to work from a more physical perspective to directly relieve the symptoms of depression as they presented and then helped the patient engage in health behaviours that had a positive influence on long-term change. In contrast, counsellors helped guide the patient to identify and confront underlying causes of depression and then find their own way forward. Usual care relied primarily on pharmacological interventions. Processes of change comprising three stages were identified within acupuncture and counselling, each with specific active components. For both interventions, participants reported that the establishment of a therapeutic relationship and their active engagement helped them develop coping strategies that in turn helped them be more effective in reducing their depression in the longer-term. Gender differences were apparent; the majority of women utilised a wide range of health behaviours, distraction and social contact, whilst men relied predominantly on cognitive strategies to manage unhelpful negative thought processes.

Strengths and Limitations

Qualitative analysis of participants' reports of acupuncture and counselling compared with usual care provided within a randomised controlled trial is novel. This study was nested within a 12-month randomised controlled trial of the effectiveness of acupuncture or counselling for depression compared to usual care. The 52 telephone interviews were obtained from a wide range of participants in socially diverse settings. The interviews provided rich data on the participants' experiences of depression and the treatment received in the trial. The thematic analysis was conducted using a bottom-up process to allow the themes to develop directly from the participants' own words. We have presented the positive and negative experiences of each form of treatment, whether treatment was beneficial or not, and we are able to enrich the quantitative results of effectiveness of the treatments offered with the qualitative data.

These qualitative findings are concordant with, and supplement the quantitative data[19] from the ACUDep trial which showed that participants with moderate to extreme pain at baseline had worse outcomes at three months for depression than the no-pain comparator group in all three treatment arms. Our findings extend the findings of the trial's quantitative data in two ways: firstly, they offer insight into how pain and disability may erode the internal resources available for the effective management of depression. Moreover, these limitations compromise the person's security by reducing ability to generate external resources such as financial income and social contact. Secondly, based directly on participants' accounts, our findings identify mechanisms within the processes of

For peer review only

change that are specific to acupuncture and counselling that facilitate reduction in the symptoms of depression.

Our study has some limitations. Participants may have attributed changes directly to treatment rather than concurrent, coincidental contextual changes. To capture the participants' experience in the longer-term the interviews were conducted after the participants completed their twelve-month follow up questionnaire. We accept there is a possibility of recall bias as it has long been known that there is a significant, stable association between depression and memory impairment[24] which may have altered what was recalled and how it was recalled. This lack of recall is reflected in the brevity of some of the interviews. Although all the research questions in the topic guide were covered in the interviews, the poor recall of some experiences did not permit a lengthy exploration. However, our aim was to learn more of the experiences of depression and treatment in the longer-term and it is likely that the participants recalled the aspects of treatment that were most salient to them. The lack of face-to face-contact during the telephone interviews prevented the interviewer gathering non-verbal contextual information such as social cues, body language, appearance, and setting to supplement the verbal answers of the interviewees. A face-to-face interview may have resulted in slightly longer interview; nevertheless, a recorded telephone interview was convenient and might be considered to be more acceptable to participants in terms of time and anonymity.

Comparison to other studies

That depression in the presence of pain is associated with a poorer response to treatment for the depression corresponds with previous studies of depression and pain comorbidity.[2][7] Patients with depression and comorbid pain tend to exhibit a cognitive bias specific to negative aspects of health and are more likely to report less favourable outcomes of treatment.[25][26] Many of the pain group participants had a musculoskeletal problem alongside their depression, and complained of fatigue and sleep disturbances. This cluster of symptoms has also been identified in 36% of older people suffering from osteoarthritis of the hip and knee.[27]

The characterisation of depression as a cycle of pain, fatigue and withdrawal that impacts on daily functioning and social activities is consistent with evidence showing that these factors create an enduring cycle of depression.[6][28] The cyclical nature of pain problems are known to activate catastrophic worry and accentuate the symptoms of depression; coping strategies such as relaxation and distraction techniques are a good way of regulating emotions if the pain is not too intense.[5] Older people with chronic pain and depression were identified in this study as the least satisfied with their primary care service, a finding which echoes earlier findings where patients with multiple physical complaints and depression posed a greater clinical burden[2][7] and were perceived as 'difficult' by general practitioners.[1]

With regards to the mechanisms of change, our findings identify three clear stages within acupuncture and counselling. The establishment of the therapeutic alliance in the early stages is an essential component from the outset of treatment. This extends the findings from within a pragmatic trial of acupuncture for back pain[29][30] and supports an earlier model of the process and mechanisms that contribute to ongoing change in counselling developed from the user perspective.[31] Historically, the therapeutic alliance has been regarded pejoratively as a placebo 'feel good factor' based on the grounds that most individuals seek positive feedback to reinforce their own behaviour.[32] However, where this argument focuses on 'visiting' a therapist for advice and help, it misses the point that the intervention-specific advice and positive reinforcement used in conjunction with the participant's active engagement in their rehabilitation will activate beneficial behavioural change.[8] An earlier study found that some participants had difficulty putting self-care advice into practice, even when they were intellectually committed and suggests that practitioners may need to follow up more carefully on the advice they have given.[33]

For peer review only

Implications for practice and future research

Previous work has advocated that the management of depression and comorbid pain should involve the treatment of both physical and psychological components together, and the treatments should be customised and directed to addressing comorbidities.[34] Psychiatrists and general practitioners often feel ill-equipped to adequately manage the complex presentation of symptoms associated with depression and comorbid pain. A shift in care is required from the current focus on the medical aspects of physical health to an all-encompassing approach that takes into account the bio-psychosocial effects of depression and comorbid pain.[35] Future research should investigate the effectiveness of using a sequential strategy of acupuncture for early relief of symptoms, especially where there are physical symptoms, followed by counselling to address deeper psychological issues and develop cognitive coping strategies to break out of the cycle of depression. In the meantime, for those who have both depression and physical symptoms, our evidence suggests that acupuncture could be a useful initial referral option.

CONCLUSION

Differences in the way depression is experienced by people with depression and comorbid pain impact on the participant's engagement with treatment and on the response to treatment for depression. The processes of acupuncture and counselling had specific identifiable effects that were beneficial to the majority of participants. The therapeutic relationship and participants' active engagement in recovery may play distinct roles in driving long-term management of depression and comorbid pain. This study has implications for policy makers and providers of care for primary care patients with depression and comorbid pain. Providers of care may wish to consider a short course of acupuncture to relieve symptoms of depression in patients who present with depression and comorbid pain, prior to a referral for counselling if needed.

Legend Supplement 3.
Diagram showing the participants' experiences of depression and treatment.
Numbers indicate participants reporting; Bold = participants with chronic pain; ^{D} = disabled

Acknowledgements

We acknowledge the contribution of the patients, counsellors, acupuncturists and general medical practitioners; the ACUDep trial management team; and Anne Burton for transcription services.

Contributors

Ann Hopton (MSc) is a research fellow from a nursing background whose research focuses on the non-pharmacological management of chronic pain and depression. Ann Hopton conducted the interviews and analysis, interpreted the data, drafted and revised the article.
Dr Janet Eldred is a qualitative researcher and research administrator whose interests are based on feminist theology and older peoples' lives. Janet Eldred assisted with coding, advised on analysis and gave final approval for publication.
Dr Hugh MacPherson is a practising acupuncturist and senior research fellow specialising in the effectiveness, cost-effectiveness, mechanisms and safety in the evaluation of complementary medicine. Hugh MacPherson revised critically important intellectual content and gave final approval for publication.

Ethics

Ethical approval was obtained from the York NHS Research Ethics Committee (09/H1311/75).

For peer review only

Funding

Funding: This article presents independent research funded by the National Institute for Health Research (NIHR) under its Programme Grants for Applied Research Programme (RP-PG-0707-10186). The views expressed are those of the author(s) and not necessarily those of the National Health Service, the NIHR or the Department of Health. The funders, and the University of York in its role as sponsor for the study, had no role in study design, data collection and analysis, decision to publish, or preparation of the manuscript.

Data sharing

There are no additional unpublished data pertaining to this study

REFERENCES

1 Katona C, Peveler R, Dowrick C, *et al.* Pain symptoms in depression: definition and clinical significance. *Clinical Medicine* 2005;**5**:390–5. doi:10.7861/clinmedicine.5-4-390

2 Arnow BA. Comorbid Depression, Chronic Pain, and Disability in Primary Care. *Psychosomatic Medicine* 2006;**68**:262–8. doi:10.1097/01.psy.0000204851.15499.fc

3 Bair MJ RR. Depression and pain comorbidity: A literature review. *ArchInternMed* 2003;**163**:2433–45.

4 Kroenke K, Wu J, Bair MJ, *et al.* Reciprocal Relationship Between Pain and Depression: A 12-Month Longitudinal Analysis in Primary Care. *The Journal of Pain* 2011;**12**:964–73. doi:10.1016/j.jpain.2011.03.003

5 Linton SJ, Nicholas MK, MacDonald S, *et al.* The role of depression and catastrophizing in musculoskeletal pain. *European Journal of Pain* 2011;**15**:416–22.

6 Tylee A, Gandhi P. The Importance of Somatic Symptoms in Depression in Primary Care. *Prim Care Companion J Clin Psychiatry* 2005;**7**:167–76.

7 Kroenke K, Shen J, Oxman TE, *et al.* Impact of pain on the outcomes of depression treatment: Results from the RESPECT trial. *Pain* 2008;**134**:209–15. doi:10.1016/j.pain.2007.09.021

8 Bair MJ, Matthias MS, Nyland KA, *et al.* Barriers and Facilitators to Chronic Pain Self-Management: A Qualitative Study of Primary Care Patients with Comorbid Musculoskeletal Pain and Depression. *Pain Medicine* 2009;**10**:1280–90.

9 Gensichen J, Guethlin C, Sarmand N, *et al.* Patients' perspectives on depression case management in general practice – A qualitative study. *Patient Education and Counseling* 2012;**86**:114–9. doi:10.1016/j.pec.2011.02.020

10 Kadam UT, Croft P, McLeod J, *et al.* A qualitative study of patients' views on anxiety and depression. *Br J Gen Pract* 2001;**51**:375–80.

11 Vickers AJ, Cronin AM, Maschino AC, *et al.* Acupuncture for Chronic Pain: Individual Patient Data Meta-analysis. *ArchInternMed* 2012;:1–10.

12 Lam M, Curry P. Effectiveness of Acupuncture for Nonspecific Chronic Low Back Pain: A Systematic Review and Meta-analysis. *Spine* 2013;**38**:2124–38. doi:10.1097/01.brs.0000435025.65564.b7

For peer review only

13 Hopton AK, Curnoe S, Kanaan M, *et al.* Acupuncture in practice: mapping the providers, the patients and the settings in a national cross-sectional survey. *BMJ Open* 2012;**2**.http://bmjopen.bmj.com/content/2/1/e000456.abstract

14 Smith CA, Hay PP. Acupuncture for depression. *CochraneDatabaseSystRev* 2005:CD004046.

15 Van Schaik DJF, Klijn AFJ, Van Hout HPJ, *et al.* Patients' preferences in the treatment of depressive disorder in primary care. *General Hospital Psychiatry* 2004;**26**:184–9. doi:10.1016/j.genhosppsych.2003.12.001

16 Bower P, Knowles S, Coventry PA, *et al.* Counselling for mental health and psychosocial problems in primary care. *CochraneDatabaseSystRev* 2011:CD001025.

17 Barnes D, Hall J, Evans R. Survey of the Current Provision of Psychological Therapy Services in Primary Care in the UK. Artemis Trust 2008. http://www.bacp.co.uk/admin/structure/files/pdf/2440_pl_438_artemis_report_web_small.pdf (accessed 10 Feb2014).

18 MacPherson H, Richmond S, Bland M, *et al.* Acupuncture and Counselling for Depression in Primary Care: A Randomised Controlled Trial. *PLoS Medicine* 2013;**10**:e1001518. doi:10.1371/journal.pmed.1001518

19 Hopton A, MacPherson H, Keding A, *et al.* Acupuncture, counselling or usual care for depression and comorbid pain: secondary analysis of a randomised controlled trial. *BMJ Open* 2014;**4**:e004964–e004964. doi:10.1136/bmjopen-2014-004964

20 Charmaz K. *Constructing Grounded Theory: A practical guide through qualitative analysis.* London: : Sage 2006.

21 MacPherson H, Schroer S. Acupuncture as a complex intervention for depression: a consensus method to develop a standardised treatment protocol for a randomised controlled trial. *Complement TherMed* 2007;**15**:92–100.

22 MacPherson H, Richmond S, Bland MJ, *et al.* Acupuncture, Counseling, and Usual care for Depression (ACUDep): study protocol for a randomized controlled trial. *Trials* 2012;**13**:209.

23 Braun V, Clarke V. Using thematic analysis in psychology. *Qualitative Research in Psychology* 2006;**3**:77–101.

24 Burt DB, Zembar MJ, Niederehe G. Depression and memory impairment: A meta-analysis of the association, its pattern, and specificity. *Psychological Bulletin* 1995;**117**:285–305. doi:10.1037/0033-2909.117.2.285

25 Pincus T, Santos R, Morley S. Depressed cognitions in chronic pain patients are focused on health: Evidence from a sentence completion task. *Pain* 2007;**130**:84–92. doi:10.1016/j.pain.2006.10.031

26 Rusu AC, Pincus T, Morley S. Depressed pain patients differ from other depressed groups: Examination of cognitive content in a sentence completion task. *PAIN* 2012;**153**:1898–904. doi:10.1016/j.pain.2012.05.034

For peer review only

27 Murphy SL, Lyden AK, Phillips K, *et al.* Subgroups of older adults with osteoarthritis based upon differing comorbid symptom presentations and potential underlying pain mechanisms. *Arthritis Research & Therapy* 2011;**13**:R135. doi:10.1186/ar3449

28 Alderson SL, Foy R, Glidewell L, *et al.* How patients understand depression associated with chronic physical disease – a systematic review. *BMC Family Practice* 2012;**13**:41. doi:10.1186/1471-2296-13-41

29 MacPherson H, Thomas K. Self-help advice as a process integral to traditional acupuncture care: implications for trial design. *Complement TherMed* 2008;**16**:101–6.

30 Hopton A, Thomas K, MacPherson H. The Acceptability of Acupuncture for Low Back Pain: A Qualitative Study of Patient's Experiences Nested within a Randomised Controlled Trial. *PLoS ONE* 2013;**8**:e56806. doi:10.1371/journal.pone.0056806

31 Perren S, Godfrey M, Rowland N. The long-term effects of counselling: The process and mechanisms that contribute to ongoing change from a user perspective. *Counselling and Psychotherapy Research* 2009;**9**:241–9. doi:10.1080/14733140903150745

32 Wall M, Wheeler S. Benefits of the placebo effect in the therapeutic relationship. *Complementary Therapies in Nursing and Midwifery* 1996;**2**:160–3. doi:10.1016/S1353-6117(96)80050-3

33 Evans M, Paterson C, Wye L, *et al.* Lifestyle and Self-Care Advice Within Traditional Acupuncture Consultations: A Qualitative Observational Study Nested in a Co-Operative Inquiry. *The Journal of Alternative and Complementary Medicine* 2011;**17**:519–29. doi:10.1089/acm.2010.0749

34 Lavigne GJ, Nashed A, Manzini C, *et al.* Does Sleep Differ Among Patients with Common Musculoskeletal Pain Disorders? *Current Rheumatology Reports* 2011;**13**:535–42. doi:10.1007/s11926-011-0209-3

35 National Collaborating Centre for Mental Health. CG91: Depression in adults with a chronic physical health problem: Treatment and management. British Psychological Society & the Royal College of Physicians 2010. http://www.nice.org.uk/CG91

For peer review only

Supplement 1.
Sampling frame of the participants interviewed:
NB. Declined =4; No response=3

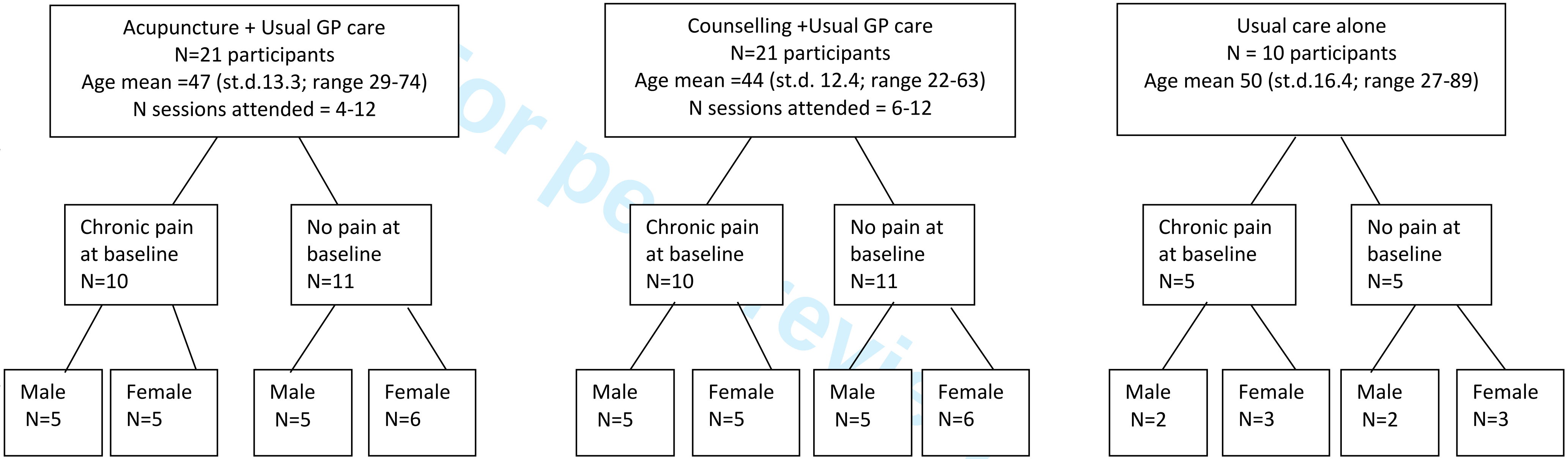

Supplement 2: Topic Guide for in-depth interviews

1. Background information:
   a) sociological profile: age gender
   b) social support: family, friends, colleagues, carers
   c) activities: physical exercise - outdoors-indoors, leisure activities, activities that expressly involve aesthetics, resting
   d) interests: hobbies, dreams, imagination, future plans
   e) food: diet, cooking, eating out
2. Personal experience of depression, co-morbidity
3. Previous experience with health services and treatments for depression
4. Experience and perceptions of the actual treatment (acupuncture, counselling and usual care) received within the trial
5. Perceived outcomes of treatment
6. Personal beliefs about the effectiveness of different types of treatments
7. Personal preferences for different types of treatments

Research Question 1)

To what extent is pain also present with depression, and how has the depression (and pain if present), changed over time as a response to receiving a course of acupuncture or counselling?

1) Could you tell me a little bit about yourself and about things you like to do, or hope to do.
   Prompt
- How has your depression affected your life over the past few months/years?

2) Could you tell me about your experience of the treatment you received for depression during the trial?
   Prompt
- It's been 12 months since your treatment started, what changes have you noticed during that time

For peer review only

Research Question 2)

What aspects of the treatment influenced long term change?

1) Has the treatment helped you understand/learn more about your depression?
   Prompt
   - Are there things you can do that will help to manage it?

2) What was it about the treatment that helped you understand or learn more about depression? And your pain?
   Prompts
   - Have you learned anything about how to manage your depression or pain? (If so, what?)
   - Have you adopted any different ways of coping with your depression or pain?

3) Has your understanding of yourself changed in any way during the trial?
   Prompt
   - How do you feel about yourself now compared to before?

Research Question 3)

How satisfied are you with the treatment received?
Prompts
- How did the treatment you received on the trial compare to treatment you have had before? e.g. GP, mental health service, private, other…?
- What aspect of the treatment did you like/dislike /gave you concern and why?
- Do you think the other treatments would be more or less effective

For peer review only

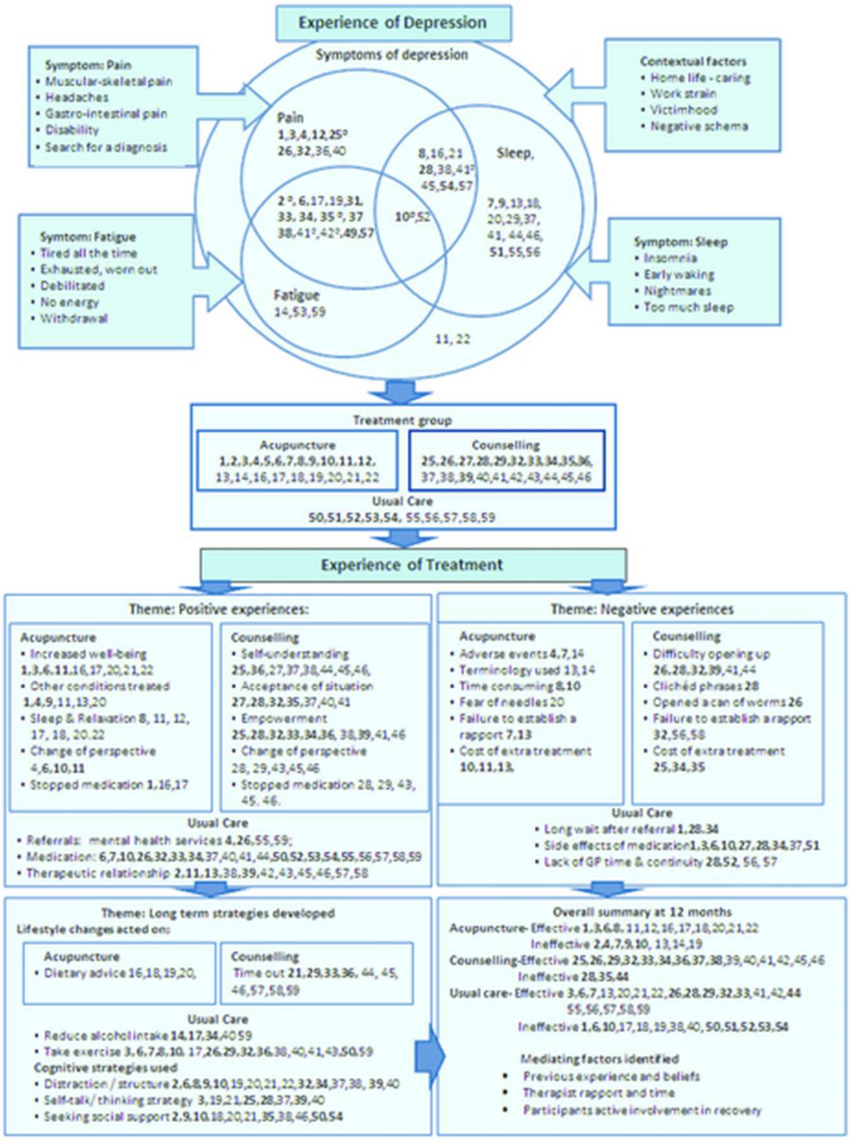

Legend Supplement 3.
Diagram showing the participants’ experiences of depression and treatment.
Numbers indicate participants reporting; Bold = participants with chronic pain; D = disabled

20x27mm (600 x 600 DPI)

Supplement 4. Symptoms of depression
“When I'm feeling really, really quite down, I find it difficult to actually get on with people. I'm very, very on the - I don’t know whether the offensive, would be the right word, in the sense that I tend to read into things too much. Or take things far too literally…And obviously the defences go out there as well, so yeah. I do things without actually realising that in the sense that I might actually snap at people. So my behaviour does change.” (p1,M,acu)
“I don t want to do things, you know. I almost, I struggle to sort of do things to motivate myself to get work done, or you know, go out and do something. And I tend to sort of stop at home and make excuses to go out really, and that’s generally how it happens…” (p3,M,acu)
“I do get an awful lot of headaches, which I've attributed to the side issues… I have suffered from headaches all my life... Since I had cancer… my migraines have become 10, 20 , 30 times worse. And my headaches are 100 times worse... It's entirely possible...that it's linked to that (depression). But I couldn't just say for certain. I get terrible tension headaches, and I get bucket loads of back pain. It's a constant feature in my life, you know. Sadly it's probably what I would just term the norm these days.” (p12,M,acu)
“Very fitful. Sometimes - it seems to be on a cycle and I keep saying I'm going to write this cycle down. Where I can't get to sleep. It's once every so often.... Sometimes it's terrible getting to sleep. And then I start thinking, which isn't helpful.” (p8,F,acu)
“Some days I just think I don’t want to go out that door. I don’t want to talk to anybody.... I get a lot of fatigue anyway, so I’m tired and I haven’t got the energy to think some days.” (p10,F,acu)
“Everything around me was just black and negative... I would never allow myself go near that again.” (p13,M,acu)
“ I feel as if I’ve got a real heavy head, as if – not a headache as in a really painful headache, but it’s just like I could just go and sit in a dark room and shut away for a time, to clear my head. It’s just like it’s a heavy feeling” (p21,F,acu)
“I also had a bowel disease called diverticulitis, and I had an operation and part of my bowel removed 2 years ago and that’s caused me problems obviously before then and since then, because they've now found that I've still got it but in another part of the bowel... I've had IBS for many years, about 15 years. And obviously when I get down and depressed, that really kicks in and the diverticular disease I've had pain all the time from that and that tends to be managed by sort of diet and medication... the likelihood is I'd end up with a colostomy, which I don't want. I'm trying to sort of manage the disease without having another operation... My IBS is certainly related to depression yeah.... When I was looking after the old dears, as I call them, I was offered an operation twice and I turned it down because I didn't want to be, you know, incapacitated and not able to look after them. Once they passed on, I was then able to address my own health problems.” (p26,M,coun)
I find that depends on my state of mind. How the day has been. If I’m feeling kind of happy and content with everything, then usually I get a full night’s sleep. And if not, then it’ll be up every couple of hours, just waking up for no apparent reason whatsoever...” (p28,M,coun)
“Everything’s such an effort... At one point I didn’t even get out of bed. I didn’t even want to get out of bed. I’d sort the children out and then I’d just go back to bed because it just hurt so much.... before I received treatment and before I was diagnosed with post-traumatic stress, you know, you’d get them off to school and I’d just left work at that point, and I would go back to bed and do nothing...” (p32,F,coun)
“at the time when things were bad I would get a lot of stomach, sort of indigestion type pain. But that was at the time when we were really sort of dealing with the sort of intensity of it…”(p38,M,coun)
“the times when I don’t feel good, my usual pattern would be to wake up like 4.00 am, something like that... well some nights having trouble getting to sleep, but more waking up and then not being able to get back to sleep” (p44,F,coun)
“I’d lost so much weight as well. I’ve never been an overweight person, but I’ve always kind of fluctuated between a 10 and a 12. But the amount of weight that I had lost, just in the sort of like 2 or 3 months, was really drastic, you know, the clothes were hanging off and I looked very gaunt in my face.”(p46,F,coun)
“I didn’t want – there were days when I just didn’t want to go out of the house.... I was just constantly tired... there were days where I would get my little girl off to school and then come home and go to bed, and do very little before I had to go and pick her up.” (p50,F,uc)
“If things aren't going too well generally here, then the arthritis seems worse. But whether that is psychosomatic or not I don’t know I really don’t know. ... one of the things I did say to the psychologist, and as bonkers as that sounds is, it’s almost like the arthritis is punishing me for something I’ve done wrong.” (p52,F,uc)
“I have got a lot of nightmares actually, that tend to wake me up... about three o’ clock, something like that. It’s a bit better now, but it did take me a long while to get back off to sleep. It wasn’t as if I woke up and nodded back off again. I could be awake for a good hour... just wanted to turn my head off.”(p57,F,uc)
“it’s stopped me doing things I used to enjoy doing. I’m not – it’s made me tired a lot of the time. Agitated, grumpy, short-tempered... I used to do a lot of reading, which I hardly do any reading now. I used to go out on bike rides and I don’t do that anymore. I used to go swimming; I stopped doing that... I get tired a lot.” (p58,M,uc)

For peer review only

Supplement 5. Contextual factors
"I can't cope with bullying at all. I seemed to always get picked on. And I'd just end up getting depressed... Well it always seemed to be a friend, they'd pick on you. You know, they'd always make you feel a fool and – it wasn't actually bullying like thumping bullying. Just make you feel out of it… And it always seemed to have happened. So I sort of won't get close to anybody anymore." (p6,F,acu)
"My first husband died. My father died. And then everything's my fault then, you see. And I'm the one in the family that, if anything goes wrong, everybody comes to me."(p8,Facu)
"I've always had a tendency to go into waves of depression throughout my life.... It's a periodic thing. It comes and it's very difficult to know why it comes. But when it comes you really hit the trough and you go down and it's very hard to pick yourself up... It makes me extremely moody. It makes me extremely irritable. It makes me not enjoy anything about me. She (mother) was in and out of many mental institutions. And it created a great deal of difficulty within my life… It did affect me and made me very introverted."(p12,M,acu)
"I'd got myself tied up into...over-valuing myself in relation to work.. I just started increasingly shutting people out that would try to get close to me. And I think that's probably because deep down I was fundamentally insecure and my kind of work outlet was my bit of success that was under my control... I'd just lost sight of things that were important." (p13,M,acu)
"I felt I aimed too high and then I didn't sort of reach what I aimed to do. So I beat myself up a bit about it."(p20,F,acu)
"It's predominantly an anxiety disorder and I think that then triggered the depression. It varies for no apparent reason whatsoever. It can be quite kind of crippling social anxiety sometimes and at other times it can be a kind of general waiting for something to go wrong; the kind of belief that there's always going to be something that's going to go wrong. And it just gets worse and worse and worse and I think it went on for such a long time that it just became how things are. That was just how my life was. Just waiting for something else to go wrong and being petrified of everything." (p28,M coun)
"I've been unemployed since November. I was employed. I left my work to look after my brother, who was poorly. And he died in November. Well I was hoping I was going to be a full-time carer for a lot longer than what it was, but it was only a matter of a few weeks, really."(p29,M,coun)
"I'm too sensitive. I believe what people say. I'm very affected by what people think about me... whether it's where we live, or the close knit community that there is here, I still feel that we're outsiders... Whether it's we give off this aura that we don't belong or because we don't do things the way they should be done around here, I don't know. But we're still not part of anything. And we've been here 8 years. It's strange... I don't know why, apart from the fact I wasn't born here."(p35,F,coun)
"I'm doing very well... But I struggle sometimes with the workload and controlling my emotions and things... Generally when I'm working, I'm a lot better. Because I like to be on the go, I like to be doing things, I like to be learning things. But I struggle though, because I'm dyslexic, and academic stuff really stresses me out. I think that's probably why I've been getting worse over the last month, because my deadline for my portfolio is tomorrow. And I know I'm worked up about that. And I've got an exam in May that I'm worked up about... at the moment I'm actually just purely overloaded."(p43,F,coun)
"I have had a job that's been pretty full on since December which has just finished. So all my energies went into that really. Which isn't healthy, I know, but I wanted to make a good job of the job, if you know what I mean."(p44,F,coun)
"I've lost a lot of confidence since I had the stroke and I'm a bit wary about going out in case in happens again and no-one's around, you know. I've lost all my confidence, really." (p51,F,uc)
"I've needed help for a long time really, but... I'm finding it really hard to deal with, you know. Letters arrive and I daren't answer letters and a lot of the times when the phone rings, I don't really want to answer the phone because, you know, there's somebody chasing me for money or threatening me with legal proceedings, or...."(p54,M,uc)
"I didn't sleep. I was very stressed and then I'd crash out as well. So I didn't sleep, I'd work really hard. I'd study really hard and then I'd crash.... I do set myself a high standard. But then I work to that standard but I don't push myself excessively to the point of collapse." (p55,F,uc)
"The way that it – the most debilitating manifestation for me I found myself spending the last three months living in one room in the house. And not going out. Escaping the world really...I had been depressed before and I kind of knew what I was getting myself into. And really just wanted to escape from there, but by the time I'd fully got myself round to wanting to tackle – it just slipped too far too quick and I couldn't actually cope on my own anymore."(p56,F,uc)
"About caring for ill spouse) it's stopped me doing things I used to enjoy doing. I'm not – it's made me tired a lot of the time. Agitated, grumpy, short-tempered. I used to do a lot of reading, which I hardly do any reading now. I used to go out on bike rides and I don't do that anymore. I used to go swimming; I stopped doing that. I get tired a lot.... I go to bed at night but a lot of the time I'm not asleep before 12 and I'm usually awake about six…I've been known to sit down in an afternoon, and I've been asleep for an hour." (p58,M,uc)

For peer review only

Supplement 6. Experience of acupuncture treatment
"Well, you're relaxing anyway, in as much as you're just lying there. ... You're talking to him and you're passing the time of day and you're relaxing while he's doing it… I didn't feel any different at all....You're lying down and my ankles, elbows, and things like that and I didn't see what he was actually doing. And I'm just lying there passing the time of day, if you like. And at the end of it, I must admit, I didn't feel any different whatsoever.... There was nothing there that was to dislike. As I say, it was just really, well, it was a morning out." (p2,M,acu)
"100 times better. Not that I dislike my GP, I think he's fantastic, but you know, I've had counselling over the years and a lot of the problem was I did child protection work for most of my working life and eventually I became so depressed with it and I think this, you know, it's not talking it's treatment you go and have done like a physical treatment. ... I think that's really positive. And a lot more positive than talking to your GP. I think you get to the stage where you've just had enough discussion with people. You just want to feel better.... It made me feel better almost straight away. Whereas talking is a lot more emotional. It's taking a lot more emotion from you and it's also making you think and do things. Sometimes you don't want to do that, you just want to feel better."(p3,M,acu)
"I'm crippled with arthritis. I walk with a walking aid... It was the acupuncture, which was really good. And I really felt it helped. You know, and it also seemed to be doing my back pain good."(p6,F,acu)
"The very first session I had, I came home and I was utterly exhausted, for days... I just couldn't move one foot in front of another. It was really quite bizarre and I said to my husband, if this is what's going to happen every week, I really don't want to do this... I went back and I said, and she was lovely, because I think at first she thought I was not going to come any more. And I said, it made me so tired, please is there something you can do. And she said, oh yes, you could adjust – I don't know, whatever it was she could adjust – and after that I never felt that wretched again. I was absolutely fine... But as soon as I told her and she adjusted things it's never happened again." (p7,F,acu)
"I found that quite from the outset, you know, I soon got to think, oh, I think I'm feeling better. Quite quickly... with some people it works and I was getting a lot from it, while I was having it.... It was absolutely fine for maybe a couple of days, I felt, oh I'm getting better. But then as I stopped it, I thought, I don't feel -- for me, I think it would have to be an ongoing thing."(p8,F,acu)
"It has helped me realise now when I'm getting depressed. It's just something trivial that's set me off and it's not such a big deal after all. I can work around that. And I think without the trial, I don't think I'd be where I am now." (p10,F,acu)
"I found I was coming out of the...building -- I don't know what you'd call it, in a more upbeat and elated manner. So for me personally, I have to say that I found the acupuncture to be a very positive thing in my life. It was something that I found made me feel an awful lot better in myself.... You have to ask yourself the question, does acupuncture work as a form of medicine. Or does some people fussing about your body give you a feel good factor?... I can't answer that... But I would say that I found acupuncture to be very positive in my life."(p12,M,acu)
"The actual process itself wasn't at all painful. Occasionally she'd hit a sort of - a stinging pain – which she always said was something to do with the meridian or some acupuncturic explanation... Walking home from it a couple of times I did feel a bit, I won't say light headed, but slightly different." (p14,Macu)
"It was quite relaxing when I was having the treatment, to be honest, which I didn't think it would be. But once I got used to it, and once the needles went in and she left me, that was my time to … that was my time to relax... I used to look forward to going." (p17,F,acu)
"It wasn't an explanation that made much sense to me in that it was about things like holding grief in your lungs or something -- what on earth does that mean? …..It just sounded like new-age nonsense, to be honest. It sounded unscientific, which I find very off-putting. You know, we need to respect the scientific method, it's how knowledge is acquired and it seemed to me that it wasn't expressed in terms that I could relate to in that kind of way.... on a kind of broader level it did seem to verge on the snake oil." (p19,F,acu)
"as the treatments went on, she sort of tried to give me more energy with different treatments. But then I was having problems with skin rashes and anxiety and things like that, so on other weeks she would treat me differently, depending on what happened that week, whether I'd been feeling more anxious or less anxious or more lethargic.... and she linked it to my menstrual cycle as well. Because when I was coming back in saying that I'd had a particularly bad week, she noticed before I did that that was probably PMS, which sounded really basic. I felt like a right idiot at the time, but obviously that kind of helped me at least find a reason for why..." (p20,F,acu)
"I think that made a big difference because I knew I could feel the treatment instantly. Once the needles were in I could feel it, so I think that really sort of got me feeling positive because I felt as if it was doing me something good within my body. It was really acting for me and working for me... I did feel different and I did feel then as if I wanted to go – you know, the more I'd been the more I wanted to go and have another session!" (p21,F,acu)

**Supplement 7. Experiences of counselling.**
"He was talking about what triggers my major depressive episodes, and it was things like, he was talking about how to recognize the small things that then build into the big things, and how to deal with them and how to recognize them and how to put in place a system of recognizing what I can control and recognizing what I can't. So it was useful in that respect." (p25,M,coun)
"I suppose that I would have liked her to have been a bit tougher. And sort of ask deeper questions, probably. It's always quite awkward I think, because you go there and you know, it's -- and you've got someone sitting opposite you smiling and thinking, well go on, say something, say something. And they're waiting for you to say something. But you don't know what to say. So it felt a bit strange." (p26,M,coun)
"I felt as if I could talk to her openly and maybe try to get to the bottom of things. Just to see if there is something that could get me on the right road sort of thing... I feel as if it did help the first few months. And then things just steadily over the weeks got back to how it was before…when I had my counselling, I'm sure for two or three months afterwards I felt a lot more positive. I was living in my other house then. I was living in H where the town's quite busy. And I'm sure I was doing more and forcing myself to do more... I remember her saying that you've got to try and be more positive and try to take every day as it comes like and try to do different things. And I think it. I know in my mind what I should be doing. But it's just doing it... I sometimes listen to the radio. But it's usually the television if I'm not sleeping. And the television's just on. I'm not particularly watching anything – it's just on." (p29,M,coun)
"Now I'm happy to put my proper clothes on that match -- as opposed to track suit bottoms and a really grotty t shirt, and not do my makeup and not do my hair. Whereas now I wouldn't dream of going out without having done my hair or my makeup. You know, I've always had my hair short, but I was letting it grow long, because I couldn't even face going to the hairdressers -- that would have been such a challenge." (p32,F,coun)
"She said I was a giver.... which means you give to everybody, but then you don't expect anything back. But she said everybody needs to have something back. You can't keep on giving and giving, because she said, you'll burn yourself out and that's not fair on you, she said, or on your family... [She] would say to me...it's all fallen back on you, hasn't it?... I only live down the road from my parents, and I can't see them struggling... They know that at the end of the day, it's me who's the reliable one, if you like.... She said, why should you have to be the one who's always doing that. Or who's expected to do it."(p33,F,coun)
"Difficult to say… external changes made things a lot easier… a second job... a bit more money... the holiday abroad booked... it was difficult to tell whether it was her counselling that had helped me or the changes in our circumstances that had improved."(p35,F,coun)
"I thought it was helpful. At the time I was on a bit of an even keel, so I didn't really need it that much. But, you know, I still had problems and everything, so I found it really helpful just getting to know my own traits a bit more so that when I do have bad episodes... I don't think I really have changed. I think I feel a bit more insightful into things and maybe a bit more wise... Talking about your life and you know, it's not like he told me what to do and how to feel about things, but just asked the right questions and get you to talk things out in your own head. It's almost like you're having a conversation with yourself, it gives you the right...to think about the right things." (p36,F,coun)
"And I thought well what, you know, what is this all about. I'm not getting anywhere. And then either she said something or I said something and it twigged... And I said to her, I said, it's like defragging a computer. And putting that stuff into little boxes, or into – and chucking out the stuff that the mind or the computer doesn't want. And as soon as I said that, things started to click into place…I don't know whether it was through trust probably, and seeing the right person, I was able to download, or offload all those things to L and she put it into perspective." (p37,M,coun)
"Before, I'd given up on life and I couldn't see a way forward. What was the point? Because I was physically unable to do anything I wanted to do. But now I look at it more positive." (p42,F,coun)
"I think I'm doing all right. I know I'm hard on myself. I'm very hard on myself... but I'm not doing it consciously. I'm doing it unconsciously. So that is harder to control. Because I'm making decisions on something and not really thinking, I'm just doing it. And thinking, yeah, I can do this. And maybe I don't even know what I'm doing to set the bar... I'm setting the bar and not really thinking about it." (p43,F,coun)
"I think my understanding of myself has changed beyond recognition. Yeah. I would say I'm a lot more self-aware and a lot more – you know, I do understand myself a lot better than I did a year ago. Not only that, I actually accept myself a lot more than I did a year ago. I can actually say, you know, this is me." (p45,F,coun)
"I don't know, she sort of – you know when somebody says something and it just actually resonates. And it's a much more real experience. Things click into place, somehow... You know, just putting it into a visual – just in a way, I don't know, something just clicked. Something that I understood. I think they sort of tapped more into me as a person, and I wasn't just being talked at or... Because sometimes you can talk and you know that you're not connecting on any level and they don't understand you and they're not actually really listening to what you're saying." (p44,F,coun)
"the counselling that I received and my domestic violence support, helped me to understand why I was feeling the way that I was and obviously to, you know, talk about the things that I was experiencing. And get it all off my chest. I'm in a much better place now... They listened. I didn't realize that I needed somebody to talk to.... So having somebody outside of that bubble.... if they needed to give me advice, then a professional, experienced opinion was easier and it would come across differently as well. And they could listen without getting upset... I think it was just having that outside person to possibly shed a tear with, who was not emotionally involved." (p46,F,coun)

Supplement 8. Experiences of usual care.
"I'm on antidepressants and I sort of go through good periods and bad periods and when I'm in a good period I try to come off the tablets, because I don't want to be dependent on them. But I realise that by coming off the tablets, I needed them. And I had to go back onto them. And because I'm so worried about being dependent on them, you know.... there doesn't seem to be any help out there, other than some tablets that will just keep me on an even keel. And sometimes the tablets even when I'm happy don't even allow me to be as happy as I should be... Yeah, it's like you're just sort of on one level all the time. Yeah, you're not depressed and you're not anxious, but you just don't get any excitement or, you know, at times when you should really. And it's really weird. A weird thing really. It's really hard to describe. You just can't seem to muster up any enthusiasm... I was more concerned about my anxiety. I asked if there was any kind of drug that I could have just to control my anxiousness, you know. Something instantly, you know, when I could feel myself getting anxious. But there was nothing that they could prescribe for me... at the moment I just feel like I've reached a dead end. There's nowhere else for me to go and nobody's going to make me any better, other than continually giving me these drugs. There's no treatment for me. There's no treatment for me unless I pay for it." (p50,F,uc)
"It's kind of got to a stalemate really. I think that's the way of it. I was trying to think about this, really. I think one of the things for me that kind of was a problem, if you'd want to call it that, was now everything's on the repeat prescription thing, normally with my former doctor, you'd go once a month or whatever, just to get a repeat prescription. And that 10 minutes was kind of just enough to almost keep you going. And my old doctor I'd known for a long time. The new one, maybe only six years or so, but now because everything's on repeat, I don't think I saw her at all last year, because you just don't need to... I normally do try and see her because obviously, you know, it's easier in a way if you do see the same person, for them as well... the GPs just don't have time, I mean, you're there 10 minutes; there's nothing much you can say, really. But I think that certainly for me, that not going regularly hasn't helped... But unfortunately now, whatever care there is out there, it's rationed – not rationed, it's just that the time and the cost and you know – I think that's just one of those things, isn't it, really."(p52,F,uc)
"I've got fluoxetine tablets and they seem to have helped a bit, because I mean before I started taking them I was suicidal – I used to think about it quite a lot... supposed to take one a day, but I take one every other day. And it seems to have taken the edge off the panic. I don't pace around the house as much as I used to do.... I worry about side effects. I'm on loads of other medication as well for... various other bits and pieces... It's not that I'm worried about getting addicted to them. I just don't want to be – I don't want to take the edge off too much, you know what I mean... I'm still really depressed and I still think about suicide a lot. The mess I'm in at the moment is just unbelievable. It would take me all day to explain the mess I'm in... it's just the people that it would upset if I did, you know. Like my son. And my dad. My dad's not well and I don't want to upset him either." (p54,M,uc)
"What he wanted to do was refer me to this, I think it was a link worker was what he said, and I went, and he said it will take a few months and you'll get your appointment... I have to be honest, the doctor that I saw... he's amazing. He is a particularly good doctor. He was actually the one that helped me with my fibre intolerance. It was always brushed off by other doctors and he realized that it was a real struggle for me and I was in a lot of pain. And with this as well, he was – he actually sat and listened and understood. And then he said, look I'll recommend you, but there is a long wait. And I'll put you on these tablets to tide you over, but I don't think it's for you. And I said, well neither do I, but I can't – and I don't know if it would have happened with any other doctor. How long I would have to have waited." (p55,F,uc)
"I have seen a counsellor but I didn't really find it beneficial... but I decided there and then that I didn't want to pursue it. You know, I find sometimes that they talk in like, clichés. Do you know what I mean?... I think she was fairly experienced. She had like lots of candles and she had lots of alternative things, you know. Yeah, but I just felt, oh, I think we're talking in clichés here. And it's not really helping."(p56,F,uc)
"I was referred to a counsellor last August, and it took six months for me to get the appointment through... And I had two sessions with this counsellor (link worker) and I was starting to feel a lot better and she said that I didn't need to see her any-more and that I was managing to cope on my own, so it was really quick, but I did feel really good actually at the time... I had a couple of sessions with her. And I did feel really good. I felt really in control and everything... It just took an awfully long time to come through. And I could really have done with it at the time, rather than having to wait six months... You really need somebody to help you, well more or less straight away. A couple of weeks really. That was the problem with it."(p57,F,uc)
"The GP was very, very good. Recognised quite early on what was happening and basically put me on some antidepressants which were nominal. They didn't really stop a downward slide but as she progressed – I mean she couldn't actually refer me to anything straight away – she just wanted to make sure she was right... But she eventually said, you know exactly what you're doing so you can regulate your own medication – it's there, whatever you need at the time. Obviously if I need a review I go every six months or whatever for a complete review. And I can't change medication myself, I can't kind of prescribe myself something new, but the medication that I do take is available. It's there on the... I can just go, I need some of that, and if it's there I can have it. She trusts me to actually look after myself as it were. To draw up my own strategies for survival, if you like."(p59,M,uc)

For peer review only

Consolidated criteria for reporting qualitative studies (COREQ): 32-item checklist

Developed from:
Tong A, Sainsbury P, Craig J. Consolidated criteria for reporting qualitative research (COREQ): a 32-item checklist for interviews and focus groups. *International Journal for Quality in Health Care*. 2007. Volume 19, Number 6: pp. 349 – 357

YOU MUST PROVIDE A RESPONSE FOR ALL ITEMS. ENTER N/A IF NOT APPLICABLE

No. Item	Guide questions/description	Reported on Page #
**Domain 1: Research team and reflexivity**		
Personal Characteristics		
1. Inter viewer/facilitator	Which author/s conducted the inter view or focus group?	Methods
2. Credentials	What were the researcher's credentials? E.g. PhD, MD	Contributors section
3. Occupation	What was their occupation at the time of the study?	Contributors section
4. Gender	Was the researcher male or female?	Contributors section
5. Experience and training	What experience or training did the researcher have?	Contributors section
Relationship with participants		
6. Relationship established	Was a relationship established prior to study commencement?	Methods
7. Participant knowledge of the interviewer	What did the participants know about the researcher? e.g. personal goals, reasons for doing the research	Methods
8. Interviewer characteristics	What characteristics were reported about the inter viewer/facilitator? e.g. Bias, assumptions, reasons and interests in the research topic	Contributors section
**Domain 2: study design**		
Theoretical framework		
9. Methodological orientation and Theory	What methodological orientation was stated to underpin the study? e.g. grounded theory, discourse analysis, ethnography, phenomenology, content analysis	Methods
Participant selection		
10. Sampling	How were participants selected? e.g. purposive, convenience, consecutive, snowball	Methods
11. Method of approach	How were participants approached? e.g. face-to-face, telephone, mail, email	Methods

For peer review only

12. Sample size	How many participants were in the study?	Results
13. Non-participation	How many people refused to participate or dropped out? Reasons?	Results
Setting		
14. Setting of data collection	Where was the data collected? e.g. home, clinic, workplace	Methods
15. Presence of non-participants	Was anyone else present besides the participants and researchers?	Methods
16. Description of sample	What are the important characteristics of the sample? e.g. demographic data, date	Results
Data collection		
17. Interview guide	Were questions, prompts, guides provided by the authors? Was it pilot tested?	Methods
18. Repeat interviews	Were repeat inter views carried out? If yes, how many?	N/A Methods
19. Audio/visual recording	Did the research use audio or visual recording to collect the data?	Methods
20. Field notes	Were field notes made during and/or after the inter view or focus group?	Methods
21. Duration	What was the duration of the inter views or focus group?	Methods
22. Data saturation	Was data saturation discussed?	Methods
23. Transcripts returned	Were transcripts returned to participants for comment and/or correction?	N/A Methods
**Domain 3: analysis and findings**		
Data analysis		
24. Number of data coders	How many data coders coded the data?	Methods
25. Description of the coding tree	Did authors provide a description of the coding tree?	Methods
26. Derivation of themes	Were themes identified in advance or derived from the data?	Methods
27. Software	What software, if applicable, was used to manage the data?	N/A Methods
28. Participant checking	Did participants provide feedback on the findings?	Methods
Reporting		
29. Quotations presented	Were participant quotations presented to illustrate the themes/findings? Was each quotation identified? e.g. participant number	Results & Supplements 4-8
30. Data and findings consistent	Was there consistency between the data presented and the findings?	Relationship to existing knowledge
31. Clarity of major themes	Were major themes clearly presented in the findings?	Results
32. Clarity of minor themes	Is there a description of diverse cases or discussion of minor themes?	Results Negative views presented

For peer review only

1 ACUDEP Qualitative Sub-study V5 19 MAY SHOWING TRACKED CHANGES bmjopen-2014-005144,

TITLE: Patients' experiences of acupuncture and counselling for depression and comorbid pain: a qualitative study nested within a randomised controlled trial.

Ann Hopton[1], Janet Eldred[1,] Hugh MacPherson[1]

[1] *Department of Health Sciences, University of York, UK*

Keywords; depression, comorbid pain, primary care, acupuncture, humanistic counselling

Address for correspondence:

Ann Hopton
Research Fellow
Department of Health Sciences
University of York
Heslington
York YO10 5DD
United Kingdom
Tel: 01904 321384
E-mail: ann.hopton@york.ac.uk

For peer review only

2 ACUDEP Qualitative Sub-study V5 19 MAY SHOWING TRACKED CHANGES bmjopen-2014-005144,

ABSTRACT

Introduction

Depression and pain frequently occur together and ~~yet we have insufficient knowledge of how this comorbidity~~ impact on outcomes of existing treatment for depression. Additional treatment options are required.~~from the patient's perspective~~. This study aimed to explore patients' experiences of depression, the processes of change within acupuncture and counselling, and the elements that contributed to longer-term change.

Methods

In a sub-study nested within a randomised controlled trial of acupuncture or counselling compared to usual care alone for depression, semi-structured interviews of 52 purposively sampled participants were conducted and analysed using thematic analysis.

Results

Differences were reported by participants regarding their experience of depression with comorbid pain compared to depression alone. Along with physical symptoms often related to fatigue and sleep, participants with depression and comorbid pain generally had fewer internal and external resources available to manage their depression effectively.– Those who had physical symptoms and were receiving acupuncture commonly reported that these were addressed as part of the treatment. For those receiving counselling,– there was less emphasis on physical symptoms and more on help with gaining an understanding of themselves and their situation. Over the course of treatment, most participants in both groups reported receiving support to cope with depression and pain independently of treatment, with a focus on relevant lifestyle and behaviour changes. The establishment of a therapeutic relationship and their active engagement as participants were identified as important components of treatment.

Conclusion

Participants with and without comorbid pain received acupuncture or counselling for depression, and reported specific identifiable treatment effects. The therapeutic relationship and participants' active engagement in recovery may play distinct roles in driving long--term change. For patients who present with both depression and physical symptoms of care may wish to consider a short course of acupuncture to relieve symptoms prior to a referral for counselling if needed. ~~This study has implications for policy makers and providers of care for primary care when considering referral of patients with depression and comorbid pain.~~

ISRCTN63787732

Strengths

- The 52 telephone interviews were obtained from a wide range of participants in socially diverse settings and provide rich data on the participants' experiences of depression and the treatment received in the ACUDep trial.
- The thematic analysis was conducted using a bottom-up process to allow the themes to develop directly from the participants' own words, and we present the positive and negative experiences of each form of treatment, whether treatment was beneficial or not.
- Our findings identify mechanisms within the processes of change that are specific to acupuncture and counselling that facilitate reduction in the symptoms of depression.

Limitations

- Participants may have attributed changes directly to treatment rather than concurrent, coincidental contextual changes.
- There is a possibility of recall bias; however, it is likely that the participants recalled the aspects of treatment that were most salient to them.
- Telephone interviews prevented the interviewer gathering non-verbal contextual information, although this form of interview was more acceptable to participants than a face-to-face interview.

For peer review only

3 ACUDEP Qualitative Sub-study V5 19 MAY SHOWING TRACKED CHANGES bmjopen-2014-005144,

INTRODUCTION

Chronic pain is commonplace in half to two-thirds of participants with major depressive disorder.[1][2] The association between pain and depression becomes stronger as the severity of either increases,[3][4] and the impact of both problems on each other plays an important role in the development and maintenance of chronicity in health problems.[5] Participants with depression and comorbid pain are difficult to diagnose, feel an increased burden of disease, tend to rely heavily on health care services and are more difficult to treat.[6] Identifying and managing the pain symptoms that commonly occur alongside symptoms of depression may be important in improving depression response and remission rates.[7] Previous qualitative research reports that patients with comorbid pain and depression identify the ineffectiveness of existing pain relief strategies, a lack of tailoring strategies to meet personal needs and difficulties with patient –physician interaction as barriers to the effective self-management of their symptoms.[8] Patients appreciate a health-care approach that is individualised [9]and are open to the value of complementary-health-care.[10] Two health-care options that offer these attributes are acupuncture and counselling.

There is a growing evidence base in support of the effectiveness of acupuncture for a range of musculoskeletal conditions;[11][12] however, despite its widespread use by participants[13] there has been limited evidence for acupuncture as an effective treatment option for depression.[14] Patients' with strong preferences for psychotherapy or counselling for depression are not likely to engage in antidepressant treatment,[15] yet the evidence for counselling as a treatment for depression is limited[16] despite widespread utilisation in primary care in the UK, with around 90% of general practices providing on site counselling services.[17] To address this evidence gap, a randomised controlled trial (ACUDep) compared acupuncture or counselling to usual care as treatments for primary care patients with ongoing depression.[18] The results showed that both acupuncture and counselling were clinically effective in reducing depression in the short to medium term.

In a quantitative sub-study nested within this trial, which focused on the effect of comorbid pain on the outcome of treatment for depression,[19] it was found that approximately 50% of the ACUDep participants had co-morbid pain with depression and that the participants' pain scores at baseline predicted the outcome of treatment for depression at the three-month follow up point. A comparison of the treatments showed that patients with depression and comorbid pain had a better outcome in the short to medium-term with acupuncture.

The ACUDEP trial[18] wwas novel in its' investigation of acupuncture or counselling compared to usual care; yet in focusing on the clinical effectiveness of the treatments offered, the published results did not set out how the process of change occurred as a results of receiving the three treatments of acupuncture, counselling and usual care . To meet this evidence gap the aim of this qualitative study nested within the ACUDep trial, is to explore patients' reports of their experiences and identify from their reports how each intervention influences long-term change.

METHODS

Design

This research comprised a qualitative sub-study nested within a three-arm randomised controlled trial conducted between November 2009 and June 2012 of acupuncture or counselling provided as an adjunct to usual care compared to usual care alone alone.[18] In this nested qualitative sub-study, a purposive sampling frame was prepared to recruit interviewees in the same 2:2:1 proportion of the main trial, to include 50% males and 50% females in each arm and to balance whether in pain at baseline or not.– The design used a- constructivist approach to grounded

theory[20] to ensure that the theories were constructed by the researchers as a result of their interaction with the area of the research and the participants.

Participants

Within the ACUDep trial,[18] 755 participants aged 18 and over with a history of on-going depression and score of 20 or more on the Beck Depression Inventory (BDI-II) were recruited by general practitioners at 27 primary care practices across Yorkshire, County Durham and Northumberland. Participants were allocated remotely by the York Trials Unit, with the allocation code concealed from the recruiting researcher, to three groups in the proportions of 2:2:1 to acupuncture, counselling and usual care alone respectively. Within the acupuncture arm, 23 acupuncturists registered with the British Acupuncture Council for a minimum of three years delivered up to 12 acupuncture treatments per patient, usually weekly. Treatments were tailored to individual participants' needs within a trial protocol,[21] which included treating symptoms according to traditional Chinese medicine theory and the integration of relevant life-style support into the treatment strategy based on acupuncture-specific advice if considered appropriate by the acupuncturists. Within the counselling arm, 37 counsellors registered with the British Association of Counselling and Psychotherapy provided up to 12 sessions of humanistic counselling delivered according to a trial protocol[22] which stated: "Counsellors will use empathy and advanced listening skills to help clients express feelings, clarify thoughts, and reframe difficulties, but they will not give advice or set homework." Deviations from the trial protocol were permissible if considered necessary and recorded in the participant's log book. Usual care comprised the treatment that patients typically receive in primary care along with over-the-counter medication. Commonly prescribed medication included anti-depressants and analgesics. Participants were followed up over a period of 12 months after randomisation by postal questionnaire at 3, 6, 9 and 12 months. On receipt of their final 12–month postal questionnaire and based on a sampling frame (see Supplement 1), a sample of participants who had previously consented to be contacted for an interview -were invited to engage in a telephone interview of approximately 30 minutes duration. Altogether 52 people consented to a one-to-one, audio-recorded, semi-structured telephone interview for this study.

~~In this nested qualitative sub-study, a purposive sampling frame was prepared to recruit interviewees in the same 2:2:1 proportion of the main trial, to include 50% males and females in each arm and to balance whether in pain at baseline or not. On receipt of their final 12 month postal questionnaire and based on a sampling frame (see Supplement 1), a sample of participants who had previously consented to be contacted for an interview were invited to engage in a telephone interview of approximately 30 minutes duration. Altogether 52 people consented to a one-to-one, audio-recorded, semi-structured telephone interview for this study.~~

Interviews

A researcher (AH) interviewed all 52 participants. The researcher was unknown to the participants prior to the interview, therefore the interview opened with an introduction designed to set the participant at ease, to reveal the context for their depression, and to draw out the participant's account of treatment received as part of the trial. Prompts from a prepared topic guide were used to elicit the participants' experiences of depression and treatment. (See Supplement 2) The topic guide had been piloted previously and granted ethical approval by the University of York ethics committee.

All the interviews were conducted between February and May 2012. On average the interviews lasted approximately 25 minutes (range 11-46 minutes). Three participants were ill and felt fatigued, but were eager to participate, therefore exploration of the questions for these people was limited in time to prevent over-burdening them. To encourage participants to relax, each participant was asked to introduce themselves by speaking about things they like to do or hoped to do and then

For peer review only

how depression had entered their lives, before moving on to the research-related questions within the topic guide. Interviews were audio-taped, transcribed verbatim and checked for accuracy. All recordings were of sufficient clarity and content that no repeat interviews were necessary. Each transcription was checked to remove any names and assigned a participant identification number.

Analytical methods
An inductive thematic analysis[23] was used to search across the dataset of 52 transcribed interviews. Using a constructivist approach to grounded theory,[20] data was analysed initially by AH by reading each transcript several times, then annotating ideas to generate a list of potential inductive codes developed from the dataset to capture and summarise the participants' experiences. Coding was performed sequentially on each transcript, initially without software, working systematically throughout the entire dataset. As codes were identified, they were recorded and organised on an Excel spread-sheet; sections of text that demonstrated that code were then added and collated moving back and forth across the data set making comparisons with previous data in an iterative process.

Coding and extractions were checked by JE to verify that the participants' experiences were reflected and summarised accurately. As coding progressed, comparisons were made between codes and phrases and those with similar context or concepts were grouped together. This process was conducted within each interview and across interviews resulting in a codebook of 33 codes, seven subthemes and two themes associated with the experience of depression, (Table 1) and a codebook of 35 codes and four themes associated with the experience of the treatment; (Table2). Saturation for the main themes occurred within each treatment option within 5-7 interviews. The coding and identification of themes were discussed and developed throughout between AH, HM and JE.

**Table 1. Coding tree; A summary of the relationships between codes, subthemes and themes for the experience of depression**		
**CODES**	**SUBTHEMES**	**THEMES**
Muscular pain		
Headaches		
Gastro-intestinal pain	Pain	
Disability		
Search for diagnosis		
Tired all the time		
Exhausted- worn out		
Debilitated	Fatigue	
No energy		Reported symptoms of depression
Withdrawal		
Insomnia		
Early wakening		
Nightmares	Sleep disorders	
Too much sleep		
Low self esteem		
What other people think		
Guilt and self-blame	Negative schema	
Black and white thinking		
Family life		
Motherhood		

For peer review only

Caring	Home life	
Bereavement		
Isolation		
Shift pattern		
Work overload	Work strain	Contextual factors
Drive, motivation		
High bar, aiming high		
Crime		
Bullied/ threat/ physical abuse	Victimhood	
Self-harm		
Childhood		

To understand the participants' individual experiences of depression and the treatments received, the codes and themes were developed into a diagram (see Supplement 3) and populated with participants' identity numbers. This enabled the researcher (AH) to trace each participant and their journey throughout the process, and to make distinctions between those with depression and comorbid pain, and those with depression alone.

Throughout this paper the themes are illustrated with quotes that capture and embody the participants' experiences embedded within the analytical narrative as suggested by Braun and Clarke.[23] Additional illustrative quotes are set out in Supplements 4-8.

Table 2. Coding tree; A summary of the relationships between codes and themes for the positive and negative experience of treatment; factors that influence long term change and mediating factors

**CODES**		**THEME**
Beliefs and attitudes	Pre-treatment factors	
Previous experience of treatment		Mediating factors
Participant engagement	In treatment factors	
Therapeutic relationship		
Change of perspective	(common to all arms)	
Reduced medication		
Relaxation	(acupuncture)	
Complaints & symptoms treated		
Self-understanding	(counselling)	Positive experiences
Acceptance of situation		
Empowerment		
Effective medication	(usual care)	
Referral to NHS mental health services		
Adverse events	(acupuncture)	
Terminology		
Time consuming		
Fear of needles		

For peer review only - http://bmjopen.bmj.com/site/about/guidelines.xhtml

Difficulty opening up	(counselling)	
Clichéd phrases		Negative experiences
Opened a can of worms		
Expense		
Side effects of medication		
Long wait after referral	(usual care)	
Lack of general practitioner time and continuity		
Ageism		
Exercise advice		
Dietary advice		
Reduce alcohol intake	Lifestyle strategies	
Take time out		
Day to day structure		Long term behavioural change
Distraction		
Thinking strategies learned		
Self- talk & Stoicism	Cognitive strategies	
Social contact		
Seeking support		

RESULTS

Participants recruited

Of the 755 participants randomised in the ACUDep trial, 674 completed their 12-month follow up; 518 had consented to be interviewed and of these 518, 464 (89%) were potentially eligible to join the study. Of the 61 participants invited, four declined participation and three did not respond. A total of 52 participants, comprising 24 men, and 28 women with an age range of 22 to 89 years (mean 46 years, SD 13.8) were interviewed. At baseline, 26 of these participants had reported having moderate or extreme pain or discomfort on the EQ5D questionnaire; these people formed the pain group, the remainder formed the no-pain comparator group. As part of the ACUDep trial, 22 of the 52 had been randomised to receive acupuncture, 20 to counselling and 10 received usual-care alone. A summary is presented in the sampling frame in Supplement 1. On average, those allocated to acupuncture attended 11 sessions (range 4-12) and those allocated to counselling attended 10 sessions (range 6-12).

Symptoms experienced with depression and contextual factors

Participants reported a range of symptoms they experienced concurrently with depression: predominantly pain, fatigue and sleep disorders (see Supplements 3-4). Most participants with depression and comorbid pain suffered from either persistent headaches or moderate to extreme muscular-skeletal pain that predated the onset of depression and compromised their mobility, to the extent that four men had considered suicide, and one man had attempted suicide. Almost half of those in pain experienced sleep disturbances or an overwhelming loss of energy to the point where much of their time was spent in bed, withdrawing from social and day-to-day activities. (See Supplement 3)

> *"There are times when things like headaches and neck aches prevent sleep... I sort of drift 48 hours without sleeping... I have in the past been, if you like, down for a prolonged period of time...just sleep and sleep and sleep and sleep and wake up and do something for half an*

For peer review only

> *hour and then go back to sleep again. I can just about function with pain killers... The only time I did try to top myself that led to even more depression because I couldn't even do that right! I'd taken a boat load of the diazepam I was on, to try and calm me down. I'd sort of stock piled some of that…washed it all down with Glenfiddich, and instead of, you know, just shuffling off quietly, all I did was end up waking up feeling absolutely dreadful in a puddle of my own vomit, and it was one of those things where, you know, it took me weeks afterwards, thinking well, I can't even kill myself properly."*(p25,M,coun)

With regards to contextual factors, for the majority of the pain group, the pain they experienced had compromised their ability to work. Very few had social support, and for some, their being at home meant that they were the family member available to take on a caring role for a relative, which incurred further stress, ill health and isolation. (See supplement 5)

> *"Because I was off and not working I was able to have the time to look after my elderly mother and aunt who were both in their 80s. So I was their carer for 4 years and unfortunately I lost both of them and my father 4 years ago, within 6 months of each other...My IBS is certainly related to depression yeah...When I was looking after the old dears, as I call them, I was offered an operation twice and I turned it down because I didn't want to be, you know, incapacitated and not able to look after them. Once they passed on, I was then able to address my own health problems."* (p26,M,coun)

In summary, for the participants with depression and comorbid pain, the symptoms experienced impacted on the most basic level of physiological needs, and reduced their ability to engage in social activity, whilst the contextual factors compromised their security through reduced income. Together these factors suggest that this group of people have few internal and external resources remaining to effectively manage their depression.

Of the participants who were pain-free at baseline, several people complained of tension headaches or gastro-intestinal symptoms they experienced at times of heightened stress and anxiety. Several others identified distinct patterns of disordered sleep: either difficulty in settling to sleep or a pattern of early wakening. Similar to the pain group, a few tended to withdraw at times when they felt particularly low in mood.

> *"I get a lot of stomach problems actually when I feel depressed...And of course, really tired as well – very, very tired when I'm feeling down…Sometimes I find it really hard to cope with people. I can possibly be a bit grumpy sometimes, or really quiet. Because I can't really face talking to anybody on certain days...I just can't bear it*." (p57,F,uc)

Regarding contextual factors, the majority in the no-pain comparator group were in full or part-time employment or were relatively affluent retired professional people. For many, their experience of depression concerned feelings of low self-esteem brought about by high expectations of themselves within their working life, or hectic social schedules. Others experienced low self-esteem and threats to their security from bullying at work or as a victim from domestic violence.

> *"We're also dance teachers, which is supposed to be a hobby but has somehow involved taking over our lives... we're teaching three nights a week, so it's a bit of a commitment for a hobby... Because when we started teaching last May time... that did give me more of an impetus to actually make the effort, because once you've got teaching you've got to go, because you're going to let people down. And I'm always glad that I do. If I'm having low days now, I'm always glad that I've been, because I'm concentrating on stuff that's*

For peer review only

> *completely outside of me or completely outside of my normal life... One of the things that's come through is that I hate letting people down. I'm very hard on myself."* (p45,F,coun)

> *"I tend to keep my things bottled up... And it really gets me down. My work suffers. My home life suffers. And everything suffers if I'm really bogged down with something….following a particular incident, or a series of incidents... I chose to or had to be the brunt for a lot of the aggression and violent behaviour that this man displayed at the time. So if you like, there was a particular traumatic... there was a starting point for it."* (p38,M,coun)

In general, the no-pain comparator group experienced fewer demands physiologically. Most had their basic security and social needs met; this group had larger reserves of both internal and external resources available to them to cope with their depression.

Processes of change reported by those receiving acupuncture: positive and negative experiences
The processes of change identified within the data formed three stages: primarily, developing a therapeutic relationship; secondly, the individual diagnosis and treatment of symptoms; and finally, engendering changes in health behaviours. Within the pain group and the no-pain comparator group, the positive experiences tended to facilitate the process of change at each stage, whilst negative experiences contributed to the barriers to change. Most participants welcomed the opportunity to try something different for their depression. The acupuncturists' understanding of their symptoms and explanations of how acupuncture might help their particular problems initiated the development of a therapeutic relationship. In contrast, two people described their acupuncturist as brisk, efficient and professional, yet lacking in bedside manner.

> *"She was very positive about things...I think you have more of an intimate relationship with the person doing that rather than just a person in an office somewhere. You're physically involved." (*p11,M,acu)

> *"They could do with a course in empathy.*" (p22,F,acu).

Within the second stage of treatment, for most participants, the therapeutic relationship was further fostered through the acupuncturist listening to the participants' concerns, and treating the symptoms of depression depending on what was diagnosed to be of most importance to the participant at that point. Within the pain group, several people experienced relief from musculoskeletal pain which tended to last for a few hours or days after the session and improvement commonly built up over several sessions. Several also reported feeling deeply relaxed during the sessions and an uplifting sense of well-being afterwards.

> *"I thought it was quite a strange sort of feeling, but I sort of felt better quite quickly. And then I went to the second and the third, it was… it completely lifted my mood and it made me feel more motivated…It was almost as if a weight had been lifted off my head and all of a sudden I felt like some energy had come back."* (p3,M,acu)

With regards to negative experiences, one woman reported extreme tiredness after the acupuncture session, a problem that was addressed by the acupuncturist by adjusting treatment during the next session, and one man with extreme back pain attributed needling pain to his damaged nerves from a previous injury. Both of these participants concluded that the treatment went well; however they also reported, *"It was not for me."* (p4,M, acu; p7,F,acu)

> *"she'd actually hit at the root for a problem that I have with my pain, because I think at one time she put a needle in me and I kicked her. Without wanting to I involuntarily kicked her.*

For peer review only

> *And she'd obviously hit upon -- I think there was one time when I kicked out and more than a few occasions where she'd twitched a nerve that obviously."* (p4,M,acu)

In contrast, within the no-pain group two participants who were worried about the potential pain of needling prior to starting the treatment later attributed the needling sensations to the healing process. Three men thought they would have been equally relaxed if they had just rested or gone for a massage, and two other participants found the sessions too time-consuming.

> *"A little bit sceptical as to whether the treatments (acupuncture) work anyway. So it was for me like, if I go and get a sport massage, it was like the equivalent of that..."* (p13,M,acu)

Factors that influenced long-term change reported by those receiving acupuncture.
As treatment progressed, many participants reported that their acupuncturist began guiding them to make changes to their lifestyle in order to engender beneficial long-term outcomes. For most people with pain, fear of pain and potential injury posed a barrier to engaging in physical activity. The majority of the pain group reported being encouraged to take up gentle exercise for their overall health and they also distracted themselves during periods of low mood.

> *"The things, they seem so small, but they are important. Things like, getting out and going for a walk and getting some fresh air. And just opening your eyes in the mornings and trying to cope with life."* (p7,F,acu)

> *"He started about exercise, you know, how that can make you feel more up…"* (p8,F, acu)

> *"I read a lot and try to keep my mind off it. Really."* (p9,F,acu)

One man developed his own technique based on how he felt during the acupuncture to help him manage low moods, whilst another relied on monthly acupuncture treatments alone to stay well; another considered further treatments but found the cost prohibitive.

> *"I sort of developed this technique and I don't know, it was like… The way I was feeling during the acupuncture... I sort of clung on to this feeling that, or this technique of gaining that feeling, so I remember on a couple of occasions where I was out and about walking, and thinking about things that would normally would start leading me to start feeling a bit down, but it was like I'd been given this tool in my head and I just sort of – it just sort of went onto auto-pilot. It was like pulling those feelings away and just sort of throwing them away....Well, it lasted for a while but it started subsiding."* (p1,M,acu)

> *"I go for acupuncture now once a month and I find that any more than a month and I can feel myself sort of slipping and feeling really, you know, starting to get worse again. And then I go and I feel much, much better…"* (p3,M,acu)

The advice given to the no-pain comparator group was qualitatively different. Acupuncturists advised on dietary change, the reduction of caffeine and alcohol, and relaxation, which varied with the presenting symptoms and by gender. Those participants with least rapport also tended to be those who were less willing to make behavioural changes.

> *"She also helped with giving me other things that I can do. Suggesting different foods for me to eat to make me feel more energetic.... I was cold all the time and that made me feel more lethargic as well, because all I wanted to do was stay in and go to bed and stay warm. So she*

For peer review only

> *was suggesting that I literally ate warmer foods, and she gave me a list of sort of Chinese medicine sort of foods that they had."* (p20,F,acu)

In general, the process of change evolved in three stages. The therapeutic relationship and active engagement in recovery acted as mediators of the outcome throughout each stage of the process. In the short term, acupuncture often relieved physiological symptoms of depression and of comorbid pain. Longer-term improvement in depression was developed through the participants' active engagement in health promoting behaviours, supported by a positive therapeutic relationship. Several participants with comorbid pain had less physical ability to engage in lifestyle changes and tended to be the passive recipients of care. These participants often had fewer external resources in the form of finance and social contact to manage their depression and comorbid symptoms in the longer-term. Additional quotes are presented in Supplement 6.

Processes of change reported by those receiving counselling: positive and negative experiences
Based on their previous experiences of counselling the majority of participants spoke of their low expectations of counselling. However when engaged with the counselling process within the trial, most reported being relieved to have someone to talk to in confidence. For both the pain and the no-pain groups, the process of change followed a common pathway: beginning with the participants' disclosure of personal information and being listened to.

> *"I found that process to be very valuable…I found X was very much listening and empathising, but maybe offered interpretation a bit more than the National Health person."* (p37,M,coun)

For most participants this two-way active engagement appeared to nurture a therapeutic relationship between the participant and counsellor. Four male participants welcomed the opportunity to speak to a male counsellor, a choice which put them at their ease, and facilitated the process. Three others found difficulty engaging with their counsellor and attributed this problem to a personality clash. This presented an early barrier to the process of change.

> *"A lot of it does depend on who the counsellor is…I'm saying probably same sex works better. They probably have a clearer understanding of the male mind... I found him particularly sympathetic and, you know, very constructive. I think that was...I was very pleased with the way it went..."* (p40,M,coun)

> *"Every single counselling cliché that you have about, oh it's parenting issues – she kind of wheeled them all out one after the other and they were already things that I'd thought about, considered and looked at and examined to the nth degree and then thought, no that's not the problem... It felt like she was reading a script almost – like a guidebook to deal with this kind of disorder...it was almost the complete opposite of what I felt like I needed."* (p28,M,coun)

A second stage of the process of change was often identified as occurring around midway within the course of treatment. The iterative process of participants' disclosure continued, with deeper exploration of their past, which helped to clarify the participant's understanding of themselves and their situation.

> *"At the end of it, it actually for me opened up a can of worms really, and I think it did me more harm than good. A lot of my problems, especially with low self-esteem, come from the way I was brought up by my parents and my father especially. And its stuff that I'd never addressed and it brought it all out, actually. And I actually felt worse at the end of it... I don't*

For peer review only

feel so bad about it now, because I recognise why I am like I am and some of the problems I have, where they come from... Although I say they'd opened up a can of worms, and brought some upsetting experiences back... I think it was good to do that. Because those sort of things had been bottled up for many, many years... it's actually made me address them." (p26,M,coun)

"It made me realize that I just held everything in. From being a little girl, everything that had ever bothered me it was never talked about. You know, I'm quite lucky that I've never had any real abuse or anything like that. It's just that I've got memories of being a child and things were said and it hurt. And I just locked it away. And I did that for years." (p41,F,coun)

Several participants realized what factors triggered and perpetuated negative thoughts, some of which were unfounded. For one man with chronic pain, this meant going through a grieving process for the loss of his former way of life before setting in place new ways of thinking and coping.

"You know, I think that was the big thing that I got from it, you know – that I could see myself more positively after having the time with him. And understand that some of the negative thoughts that were coming to my mind were not reality, if you like. To let them sail past and focus on the good things that I've done in the past." (p38,M,coun)

"Everything that defined what I was has now gone. And it took an awful lot of grief, if you like, to come round to the fact that it was worth trying again". (p25,M,coun)

The use of metaphors was particularly useful for de-cluttering unnecessary thoughts about their past, regaining perspective, setting their problems into context, and focussing on what was important.

" the discussions were much more free than I'd kind of anticipated they might have been, was using metaphors and analogies and stuff like that, to be able to describe things and move through things. And the pictures were just coming to me in my head, like. I had one which was sort of like a circuit board and it felt like some of the wires were not quite wired up properly and they weren't working and stuff like that. And I can kind of track the metaphors throughout the whole process and it feels like it was much more of a – like it all opened up." (p38,M,coun)

Factors that influenced long term change reported by those receiving counselling

The final stage in the process of change was directed towards enabling the participants to maintain progress independently. Gender differences became apparent in the coping strategies adopted; the majority of women took up health and well-being strategies. Compared to the women in the pain group, the women in the no-pain comparator group were able to use a wider range of resources to cope with, and engage in social activities more easily. One woman recalls being given cognitive behavioural homework to overcome a particular anxiety.

"She gave me sort of little exercises. I found it very difficult to walk down to a friend of mine. She lives in quite a built up area... People were sitting out in their gardens and I found it very intimidating. I didn't like it. I'd become really sweaty, short of breath walking down through her estate to go and see hear... basically she just taught me to get a grip on myself really, by pointing out, you know, that everything was going to be all right... short sharp steps really. And that I'd got the coping mechanisms and I could do it." (p32,F,coun)

Many male participants appear to have continued to practise the cognitive strategies learned within the earlier sessions, and applied them to their life outside the sessions. However, male participants

For peer review only

with depression and comorbid pain found greater difficulty sustaining these strategies and returned to their general practitioner for further help.

In summary, the majority of participants had had previous experience of counselling; however, their initial low expectations of success receded as the course of treatment progressed. A few counsellors practiced a more directive intervention than humanistic counselling, according to the need of the participant. The process of change comprised three stages, each mediated by the quality of the relationship and the participant's active engagement. Additional quotes are presented in Supplement 7.

Processes of change reported by those receiving usual care

Participants in all three arms received usual care throughout the trial. The process of change within usual care was less evident. Differences in the appraisal of general practitioner care were apparent: three older participants with depression and comorbid pain who were allocated to usual care alone complained of a lack of understanding and continuity of general practitioner and they felt abandoned without hope. One 89-year-old lady reported:

> *"I would never go to a doctor again. I am, because I suffer a lot of pain that I needn't have done if he'd been different…if he'd have listened to me instead of just pooh-poohing it off and saying, oh no, it's not that. If he'd have really listened to what I was saying, he could have done more for me...he's ignored what I've told him. Well, in fact he's very often just ignored it all together. Pretended I hadn't said it... You see, at my age you can't really change doctors. There's not many doctors want to take somebody on that's 90 years old, do they? When I'm having a really bad day... you know, and I feel I can't turn even to the doctors, you know, then yeah, I do get depressed."* (p51,F,uc)

In contrast, the majority of participants who were pain free pointed to a relationship based on trust as being a component of their steady improvement over time. For most people, a regular monthly 10-minute consultation was helpful and constructive. A few felt that their general practitioner had made additional time for them when they were most in need.

> *"He sees me every month. I have monthly meetings with him just to have a general chat about how things are... I get on fine with him. As I say, I can talk about just about anything with him, so I suppose in a way, he's sort of, if anything he's been maybe a counsellor for me, because, you know, I can sit and talk to him about stuff..."* (p58,M,uc)

All participants at some time had been prescribed antidepressant medication. The majority of participants on long-term antidepressant medication raised concerns about the side effects; participants in the pain group were particularly concerned about the potential effects of mixing medication for their other medical problems with their antidepressants. A few acknowledged that they needed antidepressants to maintain long-term stability.

> *"Staying on medication, it has transformed my life and made everybody else's life around me better as well. And I wish I'd have done it sooner... It must be four or five years now and yes, it's been life transforming... When I started on my medication and I could realize the difference – the two people I was, almost."* (p7,F,acu)

In addition to medication, during the period of the trial general practitioners referred participants to a range of secondary services: two young women received three sessions with a mental health link worker, an intervention which had enabled them regain control of their lives; three others had been

For peer review only

advised to try online cognitive behavioural therapy which two found to be easily accessible and effective; one man at risk of suicide was referred for urgent psychiatric help.

> *"I was referred rapidly to A&E and was assessed by X the psychiatrist. And I was put on to intensive home treatment. Which was invaluable. As a condition of not being sectioned..."* (p59,M,uc)

Referrals for younger patients had been beneficial although the waiting times were long and not always found to be acceptable, leaving most patients without adequate support in a time of crisis, and without sufficient money to pay for private care.

> *"I go to the doctors and I have to wait a matter of I don't know how many months before I can get, you know, onto the counselling and you know, it's just like the moment's gone, sort of thing... Unfortunately that can cost a lot of money – I'm on benefit. I can't afford it...I don't hold out much hope."* (p50,F,uc)

Factors that influenced long term change reported by those receiving usual care: positive and negative experiences

For those who received help via a referral, the advice followed a familiar pattern: to engage in lifestyle changes, to add structure to the daily routine, and to use distraction to reduce the focus on the symptoms and negative feelings. However, without support the stoicism of 'forcing myself' was a prominent default strategy among most usual care participants.

> *"I force myself to do things and then I generally feel better"* (p57,F,uc)

> *"It's forcing myself. Well, it's a survival strategy..."*(p59,M,uc)

Overall, the continuity of always seeing the same general practitioner was reported to be important and beneficial. By contrast, some older participants with depression and comorbid pain remained caught in a seemingly hopeless cycle of seeking diagnosis and treatment for a physical complaint and without resources to seek private health care services. Most patients who received acupuncture or counselling were also happy with the attention from their general practitioner, but had welcomed the additional treatment provided within the trial as an adjunct to their usual care. Supplement 8 presents a number of representative quotes.

DISCUSSION

Principal findings

The participants' experiences of depression were a complex interplay of internal and contextual factors. Compared to participants with depression alone, participants with depression and comorbid pain had fewer internal and external resources available to effectively manage their depression in the longer-term. Acupuncture and counselling treatments were individualised interventions that operated from different perspectives. Acupuncturists appeared to work from a more physical perspective to directly relieve the symptoms of depression as they presented and then helped the patient engage in health behaviours that had a positive influence on long-term change. In contrast, counsellors helped guide the patient to identify and confront underlying causes of depression and then find their own way forward. Usual care relied primarily on pharmacological interventions. Processes of change comprising three stages were identified within acupuncture and counselling, each with specific active components. For both interventions, participants reported that the establishment of a therapeutic relationship and their active engagement helped them develop coping strategies that in turn helped them be more effective in reducing their depression in the

For peer review only - http://bmjopen.bmj.com/site/about/guidelines.xhtml

longer-term. Gender differences were apparent; the majority of women utilised a wide range of health behaviours, distraction and social contact, whilst men relied predominantly on cognitive strategies to manage unhelpful negative thought processes.

Strengths and Limitations

Qualitative analysis of participants' reports of acupuncture and counselling compared with usual care provided within a randomised controlled trial is novel. This study was nested within a 12-month randomised controlled trial of the effectiveness of acupuncture or counselling for depression compared to usual care. The 52 telephone interviews were obtained from a wide range of participants in socially diverse settings. The interviews provided rich data on the participants' experiences of depression and the treatment received in the trial. The thematic analysis was conducted using a bottom-up process to allow the themes to develop directly from the participants' own words. We have presented the positive and negative experiences of each form of treatment, whether treatment was beneficial or not, and we are able to enrich the quantitative results of effectiveness of the treatments offered with the qualitative data.

These qualitative findings are concordant with, and supplement the quantitative data[19] from the ACUDep trial which showed that participants with moderate to extreme pain at baseline had worse outcomes at three months for depression than the no-pain comparator group in all three treatment arms. Our findings extend the findings of the trial's quantitative data in two ways: firstly, they offer insight into how pain and disability may erode the internal resources available for the effective management of depression. Moreover, these limitations compromise the person's security by reducing ability to generate external resources such as financial income and social contact. Secondly, based directly on participants' accounts, our findings identify mechanisms within the processes of change that are specific to acupuncture and counselling that facilitate reduction in the symptoms of depression.

Our study has some limitations. Participants' may have attributed changes directly to treatment rather than concurrent, coincidental contextual changes. – To capture the participants' experience in the longer-term the interviews were conducted after the participants completed their twelve-month follow up questionnaire. We accept there is a possibility of recall bias as it has long been known that there is a significant, stable association between depression and memory impairment[24] – which may have altered what was recalled and how it was recalled. This lack of recall is reflected in the brevity of some of the interviews. Although all the research questions in the topic guide were covered in the interviews, the poor recall of some experiences did not permit a lengthy exploration. However, our aim was to learn more of the experiences of depression and treatment in the longer-term and it is likely that the participants recalled the aspects of treatment that were most salient to them. The lack of face-to face-contact during the telephone interviews prevented the interviewer gathering non-verbal contextual information such as social cues, body language, appearance, and setting to supplement the verbal answers of the interviewees. – A face-to--face interview may have resulted in slightly longer interview;, nevertheless, a recorded telephone interview was convenient and might be considered~~expected~~ to be more acceptable to participants ~~than a face-to-face interview~~ in terms of time and anonymity.

Comparison to other studies

That depression in the presence of pain is associated with a poorer response to treatment for the depression corresponds with previous studies of depression and pain comorbidity.[2][7] Patients with depression and comorbid pain tend to exhibit a cognitive bias specific to negative aspects of health and are more likely to report less favourable outcomes of treatment.[25][26] Many of the pain group participants had a musculoskeletal problem alongside their depression, and complained

of fatigue and sleep disturbances. This cluster of symptoms has also been identified in 36% of older people suffering from osteoarthritis of the hip and knee.[27]

The characterisation of depression as a cycle of pain, fatigue and withdrawal that impacts on daily functioning and social activities is consistent with evidence showing that these factors create an enduring cycle of depression.[6][28] The cyclical nature of pain problems are known to activate catastrophic worry and accentuate the symptoms of depression; coping strategies such as relaxation and distraction techniques are a good way of regulating emotions if the pain is not too intense.[5] Older people with chronic pain and depression were identified in this study as the least satisfied with their primary care service, a finding which echoes earlier findings where patients with multiple physical complaints and depression posed a greater clinical burden[2][7] and were perceived as 'difficult' by general practitioners.[1]

With regards to the mechanisms of change, our findings identify three clear stages within acupuncture and counselling. The establishment of the therapeutic alliance in the early stages is an essential component from the outset of treatment. This extends the findings from within a pragmatic trial of acupuncture for back pain[29][30] and supports an earlier model of the process and mechanisms that contribute to ongoing change in counselling developed from the user perspective.[31] Historically, the therapeutic alliance has been regarded pejoratively as a placebo 'feel good factor' based on the grounds that most individuals seek positive feedback to reinforce their own behaviour.[32] However, where this argument focuses on 'visiting' a therapist for advice and help, it misses the point that the intervention-specific advice and positive reinforcement used in conjunction with the participant's active engagement in their rehabilitation will activate beneficial behavioural change.[8] An earlier study found that some participants had difficulty putting self-care advice into practice, even when they were intellectually committed and suggests that practitioners may need to follow up more carefully on the advice they have given.[33]

Implications for practice and future research

Previous work has advocated that the management of depression and comorbid pain should involve the treatment of both physical and psychological components together, and the treatments should be customised and directed to addressing comorbidities.[34] Psychiatrists and general practitioners often feel ill-equipped to adequately manage the complex presentation of symptoms associated with depression and comorbid pain. A shift in care is required from the current focus on the medical aspects of physical health to an all-encompassing approach that takes into account the bio-psychosocial effects of depression and comorbid pain.[35] Future research should investigate the effectiveness of using a sequential strategy of acupuncture for early relief of symptoms, especially where there are physical symptoms, followed by counselling to address deeper psychological issues and develop cognitive coping strategies to break out of the cycle of depression. In the meantime, for those who have both depression and physical symptoms, our evidence suggests that acupuncture could be a useful initial referral option.

CONCLUSION

Differences in the way depression is experienced by people with depression and comorbid pain impact on the participant's engagement with treatment and on the response to treatment for depression. The processes of acupuncture and counselling had specific identifiable effects that were beneficial to the majority of participants. The therapeutic relationship and participants' active engagement in recovery may play distinct roles in driving long-term management of depression and comorbid pain. This study has implications for policy makers and providers of care for primary care patients with depression and comorbid pain. Providers of care may wish to consider a short course of acupuncture to relieve symptoms of depression in patients who present with depression and comorbid pain, prior to a referral for counselling if needed.

For peer review only

Legend Supplement 3.
Diagram showing the participants' experiences of depression and treatment.
Numbers indicate participants reporting; Bold = participants with chronic pain; ^{D} = disabled

Acknowledgements
We acknowledge the contribution of the patients, counsellors, acupuncturists and general medical practitioners; the ACUDep trial management team; and Anne Burton for transcription services.

Contributors
Ann Hopton (MSc) is a research fellow from a nursing background whose research focuses on the non-pharmacological management of chronic pain and depression. Ann Hopton conducted the interviews and analysis, interpreted the data, drafted and revised the article.
Dr Janet Eldred is a qualitative researcher and research administrator whose interests are based on feminist theology and older peoples' lives. Janet Eldred assisted with coding, advised on analysis and gave final approval for publication.
Dr Hugh MacPherson is a practising acupuncturist and senior research fellow specialising in the effectiveness, cost-effectiveness, mechanisms and safety in the evaluation of complementary medicine. Hugh MacPherson revised critically important intellectual content and gave final approval for publication.

Ethics
Ethical approval was obtained from the York NHS Research Ethics Committee (09/H1311/75).

Funding
Funding: This article presents independent research funded by the National Institute for Health Research (NIHR) under its Programme Grants for Applied Research Programme (RP-PG-0707-10186). The views expressed are those of the author(s) and not necessarily those of the National Health Service, the NIHR or the Department of Health. The funders, and the University of York in its role as sponsor for the study, had no role in study design, data collection and analysis, decision to publish, or preparation of the manuscript.

For peer review only

REFERENCES

1 Katona C, Peveler R, Dowrick C, *et al.* Pain symptoms in depression: definition and clinical significance. *Clinical Medicine* 2005;**5**:390–5. doi:10.7861/clinmedicine.5-4-390

2 Arnow BA. Comorbid Depression, Chronic Pain, and Disability in Primary Care. *Psychosomatic Medicine* 2006;**68**:262–8. doi:10.1097/01.psy.0000204851.15499.fc

3 Bair MJ RR. Depression and pain comorbidity: A literature review. *ArchInternMed* 2003;**163**:2433–45.

4 Kroenke K, Wu J, Bair MJ, *et al.* Reciprocal Relationship Between Pain and Depression: A 12-Month Longitudinal Analysis in Primary Care. *The Journal of Pain* 2011;**12**:964–73. doi:10.1016/j.jpain.2011.03.003

5 Linton SJ, Nicholas MK, MacDonald S, *et al.* The role of depression and catastrophizing in musculoskeletal pain. *European Journal of Pain* 2011;**15**:416–22.

6 Tylee A, Gandhi P. The Importance of Somatic Symptoms in Depression in Primary Care. *Prim Care Companion J Clin Psychiatry* 2005;**7**:167–76.

7 Kroenke K, Shen J, Oxman TE, *et al.* Impact of pain on the outcomes of depression treatment: Results from the RESPECT trial. *Pain* 2008;**134**:209–15. doi:10.1016/j.pain.2007.09.021

8 Bair MJ, Matthias MS, Nyland KA, *et al.* Barriers and Facilitators to Chronic Pain Self-Management: A Qualitative Study of Primary Care Patients with Comorbid Musculoskeletal Pain and Depression. *Pain Medicine* 2009;**10**:1280–90.

9 Gensichen J, Guethlin C, Sarmand N, *et al.* Patients' perspectives on depression case management in general practice – A qualitative study. *Patient Education and Counseling* 2012;**86**:114–9. doi:10.1016/j.pec.2011.02.020

10 Kadam UT, Croft P, McLeod J, *et al.* A qualitative study of patients' views on anxiety and depression. *Br J Gen Pract* 2001;**51**:375–80.

11 Vickers AJ, Cronin AM, Maschino AC, *et al.* Acupuncture for Chronic Pain: Individual Patient Data Meta-analysis. *ArchInternMed* 2012;:1–10.

12 Lam M, Curry P. Effectiveness of Acupuncture for Nonspecific Chronic Low Back Pain: A Systematic Review and Meta-analysis. *Spine* 2013;**38**:2124–38. doi:10.1097/01.brs.0000435025.65564.b7

13 Hopton AK, Curnoe S, Kanaan M, *et al.* Acupuncture in practice: mapping the providers, the patients and the settings in a national cross-sectional survey. *BMJ Open* 2012;**2**.http://bmjopen.bmj.com/content/2/1/e000456.abstract

14 Smith CA, Hay PP. Acupuncture for depression. *CochraneDatabaseSystRev* 2005:CD004046.

15 Van Schaik DJF, Klijn AFJ, Van Hout HPJ, *et al.* Patients' preferences in the treatment of depressive disorder in primary care. *General Hospital Psychiatry* 2004;**26**:184–9. doi:10.1016/j.genhosppsych.2003.12.001

16 Bower P, Knowles S, Coventry PA, *et al.* Counselling for mental health and psychosocial problems in primary care. *CochraneDatabaseSystRev* 2011:CD001025.

For peer review only

17 Barnes D, Hall J, Evans R. Survey of the Current Provision of Psychological Therapy Services in Primary Care in the UK. Artemis Trust 2008. http://www.bacp.co.uk/admin/structure/files/pdf/2440_pl_438_artemis_report_web_small.pdf (accessed 10 Feb2014).

18 MacPherson H, Richmond S, Bland M, *et al.* Acupuncture and Counselling for Depression in Primary Care: A Randomised Controlled Trial. *PLoS Medicine* 2013;**10**:e1001518. doi:10.1371/journal.pmed.1001518

19 Hopton A, MacPherson H, Keding A, *et al.* Acupuncture, counselling or usual care for depression and comorbid pain: secondary analysis of a randomised controlled trial. *BMJ Open* 2014;**4**:e004964–e004964. doi:10.1136/bmjopen-2014-004964

20 Charmaz K. *Constructing Grounded Theory: A practical guide through qualitative analysis.* London: : Sage 2006.

21 MacPherson H, Schroer S. Acupuncture as a complex intervention for depression: a consensus method to develop a standardised treatment protocol for a randomised controlled trial. *Complement TherMed* 2007;**15**:92–100.

22 MacPherson H, Richmond S, Bland MJ, *et al.* Acupuncture, Counseling, and Usual care for Depression (ACUDep): study protocol for a randomized controlled trial. *Trials* 2012;**13**:209.

23 Braun V, Clarke V. Using thematic analysis in psychology. *Qualitative Research in Psychology* 2006;**3**:77–101.

24 Burt DB, Zembar MJ, Niederehe G. Depression and memory impairment: A meta-analysis of the association, its pattern, and specificity. *Psychological Bulletin* 1995;**117**:285–305. doi:10.1037/0033-2909.117.2.285

25 Pincus T, Santos R, Morley S. Depressed cognitions in chronic pain patients are focused on health: Evidence from a sentence completion task. *Pain* 2007;**130**:84–92. doi:10.1016/j.pain.2006.10.031

26 Rusu AC, Pincus T, Morley S. Depressed pain patients differ from other depressed groups: Examination of cognitive content in a sentence completion task. *PAIN* 2012;**153**:1898–904. doi:10.1016/j.pain.2012.05.034

27 Murphy SL, Lyden AK, Phillips K, *et al.* Subgroups of older adults with osteoarthritis based upon differing comorbid symptom presentations and potential underlying pain mechanisms. *Arthritis Research & Therapy* 2011;**13**:R135. doi:10.1186/ar3449

28 Alderson SL, Foy R, Glidewell L, *et al.* How patients understand depression associated with chronic physical disease – a systematic review. *BMC Family Practice* 2012;**13**:41. doi:10.1186/1471-2296-13-41

29 MacPherson H, Thomas K. Self-help advice as a process integral to traditional acupuncture care: implications for trial design. *Complement TherMed* 2008;**16**:101–6.

30 Hopton A, Thomas K, MacPherson H. The Acceptability of Acupuncture for Low Back Pain: A Qualitative Study of Patient's Experiences Nested within a Randomised Controlled Trial. *PLoS ONE* 2013;**8**:e56806. doi:10.1371/journal.pone.0056806

For peer review only

31 Perren S, Godfrey M, Rowland N. The long-term effects of counselling: The process and mechanisms that contribute to ongoing change from a user perspective. *Counselling and Psychotherapy Research* 2009;**9**:241–9. doi:10.1080/14733140903150745

32 Wall M, Wheeler S. Benefits of the placebo effect in the therapeutic relationship. *Complementary Therapies in Nursing and Midwifery* 1996;**2**:160–3. doi:10.1016/S1353-6117(96)80050-3

33 Evans M, Paterson C, Wye L, *et al.* Lifestyle and Self-Care Advice Within Traditional Acupuncture Consultations: A Qualitative Observational Study Nested in a Co-Operative Inquiry. *The Journal of Alternative and Complementary Medicine* 2011;**17**:519–29. doi:10.1089/acm.2010.0749

34 Lavigne GJ, Nashed A, Manzini C, *et al.* Does Sleep Differ Among Patients with Common Musculoskeletal Pain Disorders? *Current Rheumatology Reports* 2011;**13**:535–42. doi:10.1007/s11926-011-0209-3

35 National Collaborating Centre for Mental Health. CG91: Depression in adults with a chronic physical health problem: Treatment and management. British Psychological Society & the Royal College of Physicians 2010. http://www.nice.org.uk/CG91

For peer review only
